# Global stocks and capacity of mineral-associated soil organic carbon

**DOI:** 10.1038/s41467-022-31540-9

**Published:** 2022-07-01

**Authors:** Katerina Georgiou, Robert B. Jackson, Olga Vindušková, Rose Z. Abramoff, Anders Ahlström, Wenting Feng, Jennifer W. Harden, Adam F. A. Pellegrini, H. Wayne Polley, Jennifer L. Soong, William J. Riley, Margaret S. Torn

**Affiliations:** 1 Physical and Life Sciences Directorate, Lawrence Livermore National Laboratory, Livermore, CA 94551 USA; 2 Department of Earth System Science, Stanford University, Stanford, CA 94305 USA; 3 Woods Institute for the Environment, Stanford University, Stanford, CA 94305 USA; 4 Precourt Institute for Energy, Stanford University, Stanford, CA 94305 USA; 5 Department of Biology, University of Antwerp, Antwerp, 2000 Belgium; 6 Institute for Environmental Studies, Charles University, Prague, 128 01 Czech Republic; 7 Laboratoire des Sciences du Climat et de l’Environnement, Gif-sur-Yvette, F-91191 France; 8 Environmental Sciences Division, Oak Ridge National Laboratory, Oak Ridge, TN 37830 USA; 9 Deptartment of Physical Geography and Ecosystem Science, Lund University, Lund, SE-22100 Sweden; 10 Agricultural Resources and Regional Planning, Chinese Academy of Agricultural Sciences, Beijing, 10081 China; 11 U.S. Geological Survey, Menlo Park, CA 94035 USA; 12 Department of Plant Sciences, University of Cambridge, Cambridge, CB2 3EA UK; 13 Cambridge Conservation Institute, University of Cambridge, Cambridge, CB2 3EA UK; 14 Agricultural Research Service, U.S. Department of Agriculture, Temple, TX 76502 USA; 15 Department of Ecosystem Science and Sustainability, Colorado State University, Fort Collins, CO 80523 USA; 16 Granular, Inc, San Francisco, CA 94103 USA; 17 Climate and Ecosystem Sciences, Lawrence Berkeley National Laboratory, Berkeley, CA 94720 USA; 18 Energy and Resources Group, University of California, Berkeley, Berkeley, CA 94720 USA

**Keywords:** Carbon cycle, Carbon cycle, Climate-change mitigation, Climate and Earth system modelling

## Abstract

Soil is the largest terrestrial reservoir of organic carbon and is central for climate change mitigation and carbon-climate feedbacks. Chemical and physical associations of soil carbon with minerals play a critical role in carbon storage, but the amount and global capacity for storage in this form remain unquantified. Here, we produce spatially-resolved global estimates of mineral-associated organic carbon stocks and carbon-storage capacity by analyzing 1144 globally-distributed soil profiles. We show that current stocks total 899 Pg C to a depth of 1 m in non-permafrost mineral soils. Although this constitutes 66% and 70% of soil carbon in surface and deeper layers, respectively, it is only 42% and 21% of the mineralogical capacity. Regions under agricultural management and deeper soil layers show the largest undersaturation of mineral-associated carbon. Critically, the degree of undersaturation indicates sequestration efficiency over years to decades. We show that, across 103 carbon-accrual measurements spanning management interventions globally, soils furthest from their mineralogical capacity are more effective at accruing carbon; sequestration rates average 3-times higher in soils at one tenth of their capacity compared to soils at one half of their capacity. Our findings provide insights into the world’s soils, their capacity to store carbon, and priority regions and actions for soil carbon management.

## Abstract (web summary)

Mineral-organic associations play a key role in soil carbon preservation. Here, Georgiou et al. produce global estimates of mineral-associated soil carbon, providing insight into the world’s soils and their capacity to store carbon

## Introduction

Soil organic carbon (SOC) is an integral component of terrestrial ecosystems and plays an important role in ecosystem resilience and productivity. Soil organic matter contains nutrients that support plant growth and yield, retains water and reduces runoff, and resists erosion^1^. Globally, SOC contains more carbon than the atmosphere and vegetation combined^2–4^. Climate- and land-use-induced changes to soil may alter SOC cycling and drive large terrestrial carbon sinks or sources; indeed, human land-use and land-cover change have resulted in a significant net loss of soil carbon over the past two centuries^5–7^. Improved soil management practices that promote soil carbon sequestration, especially in stable carbon pools, are needed to reverse this trajectory and mitigate climate change^8,9^.

Field observations suggest that more than half of soil organic carbon is chemically or physically associated with soil minerals^10,11^ (Supplementary Fig. 1). These interactions limit microbial access to otherwise decomposable substrates^12,13^ and, consequently, mineral-associated organic carbon (MOC) can have turnover times up to 1000 times longer (reaching 100–10,000 years) than particulate organic carbon (POC) at the same depth^14,15^. Thus, increasing MOC may be a key to lasting carbon sequestration in soils^16^. However, despite its unique role, it is still unclear how much MOC presently exists, and how much could be accrued, in soils globally across depths and geographic regions. The capacity of soils to store MOC and other persistent forms of SOC will influence the long-term trajectory of the terrestrial carbon sink. Furthermore, it is unknown whether the proximity of a given soil to its carbon-storage capacity will influence the rate of C accrual, and thus the efficacy of soil C sequestration efforts. These knowledge gaps hinder climate change mitigation pathways and soil management initiatives, and limit long-term projections of Earth system models.

Soil organic matter decomposition, and its response and feedback to climate, depends on the physico-chemical form of the organic matter. To improve projections of long-term soil-climate feedbacks, it is imperative to predict the amount, distribution, and dynamics of MOC^17^. Yet, little attention has been afforded to the explicit representation and parameterization of mineral-organic associations in Earth system models^18^. Organic matter and minerals form associations via myriad mechanisms, including ligand exchange, hydrophobic interactions, and cation bridging^19^, and the appropriate—representative yet tractable—mathematical formulation of MOC dynamics in these models is still the subject of research^20,21^. Nevertheless, existing and candidate approaches require a means to constrain the maximum mineralogical capacity (MOC_max_). This MOC_max_ is a property of the soil mineralogy^22,23^. A data-driven approach to convert readily measured soil mineralogical variables to MOC_max_ is therefore needed to achieve advances in modeling and to enable robust estimates of carbon sequestration capacity.

Here we synthesized MOC observations from 1144 soil profiles spanning diverse biomes, soil types, and climates worldwide. Our synthesis included soils with a wide range of clay plus silt mineral content (CS; 1.5–100%), mean annual temperature (MAT; −2.9–29 °C), and mean annual precipitation (MAP; 79–3806 mm yr^−1^), as well as different vegetation types and land-uses (Supplementary Figs. 2 and 3). We leveraged these observations, with insights from theory and process-based models, and demonstrated that MOC_max_ can be inferred as an emergent property from readily measured soil mineralogical variables. We explored the variability of observed MOC and used a machine learning approach to elucidate the role of environmental variables, including climate and vegetation, in driving the observed departures from mineralogical saturation. We categorized sites into natural/less-managed (forest and grassland) and intensively managed (cropland) ecosystems to further investigate the effects of vegetation type and management on the degree of MOC undersaturation across soil depths. Finally, to explore how this undersaturation affects the sequestration efficacy of soils over decadal timescales, we examined 103 carbon-accrual measurements spanning management interventions globally.

## Results and discussion

### Carbon capacity of low- and high-activity mineral soils

Studies have presented conflicting results on the importance of clay plus silt content (CS) as a single linear predictor of MOC^24–26^, with many of these analyses focusing on the prediction of bulk SOC, which contains additional pools of non-mineral-associated organic carbon (e.g., POC). As we present here, theory and model insights, as well as our extensive global data analysis, all suggest that there is no universal linear relationship between MOC (or SOC) and CS (Supplementary Figs. 4–6). Rather, this relationship depends, to a first-degree, on the combined effect of the C loading on minerals (g C m^−2^ mineral) which is a function of environmental conditions and management practices^27^, and the effective mineral area on which C can bind (m^2^ g^−1^ mineral) which is a function of the type of mineral^22,28,29^. However, since MOC_max_ is driven primarily by the amount (g mineral kg^−1^ soil) and type of mineral^23,28^, we hypothesized that if soils were partitioned based on their dominant mineral type, CS would emerge as a significant predictor of MOC_max_ globally.

To test this hypothesis, we classified soils according to their mineral type and explored whether the upper quantiles of observed MOC form distinct boundaries for different mineral categories, i.e., whether a distinct MOC_max_ specific to each mineral type arises (Supplementary Figs. 6 and 7). Here, we classified soils as containing high- or low-activity minerals (HM or LM, respectively) based on their primary composition of high-activity (illite, smectite, vermiculite, chlorite) and low-activity (kaolinite, gibbsite) clays^29–31^ (see “Methods” for the robustness of results to additional categories).

**Fig. 1:**
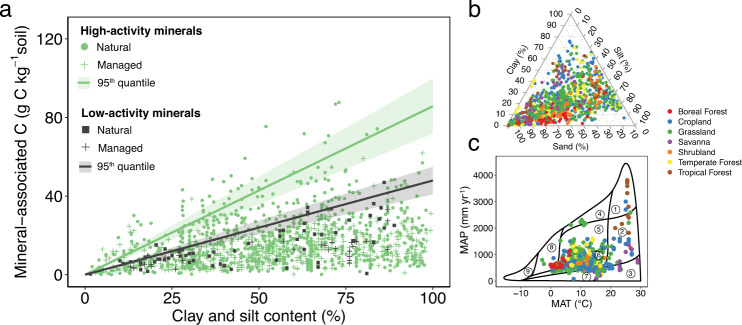
Maximum mineral-associated carbon as a function of clay and silt content for high- and low-activity mineral soils estimated from a global observational synthesis. **a** Mineral-associated organic carbon (MOC; g C kg^−1^ soil) as a function of clay and silt content (CS; %) across sites. The maximum slope (fit as the 95th quantile) for each soil type represents the intrinsic capacity of minerals to store carbon and depends on mineral composition. High-activity minerals (HM) include soils dominated by illite, smectite, vermiculite, and chlorite (*n* = 1303) and low-activity minerals (LM) include soils dominated by kaolinite and gibbsite (*n* = 93). Shading depicts 90% confidence intervals on the slope. Soils near the upper quantiles are closer to MOC saturation. Within HM and LM soils, filled markers and crosses denote natural and managed ecosystems, respectively. **b**, **c** Distribution of soil texture (**b**) and climate (**c**) across sites. Black polygons depict Whittaker’s biomes^119^ according to mean annual temperature (MAT; °C) and mean annual precipitation (MAP; mm yr^−1^) values, following: (1) tropical rainforest; (2) tropical seasonal rainforest/savanna; (3) subtropical desert; (4) temperate rainforest; (5) temperate seasonal forest; (6) woodland/shrubland; (7) temperate grassland/desert; (8) boreal forest; and (9) tundra.

Indeed, our data synthesis quantifies and supports this hypothesis, demonstrating that a robust MOC_max_ estimate can emerge as a function of only CS and the type of mineral (Fig. 1; and irrespective of vegetation type, Supplementary Fig. 8). We obtained a conservative estimate of MOC_max_ as the 95th quantile of MOC values for each mineral type, resulting in MOC_max_ estimates of 86 ± 9 and 48 ± 6 mg C g^−1^ mineral for HM and LM, respectively (Fig. 1; slope ± 90% confidence interval, following a unit conversion). These data-derived estimates of MOC_max_ provide a maximum carbon potential for a given CS and mineral type, which can be used to inform the location and management practices of soil restoration and sequestration efforts (Supplementary Discussion). These values broadly agree with regional studies that have included high-activity minerals at comparable soil depths^28,32,33^, but are significantly greater than studies that have omitted mineral activity^24,34–37^. Given the important role of mineralogy (Supplementary Fig. 4), our data-driven MOC_max_ estimates are also a notable update to regional studies that have reported a maximum carbon potential across European soils (e.g., 45–50 g C kg^−1^ soil based on model-predicted values in ref. ^11^). While it may be difficult for soils to reach MOC_max_ in practice, the proximity of a given soil to its maximum capacity is an important factor for determining its effectiveness in sequestering additional carbon (see Supplementary Fig. 9 for a conceptual schematic)^38,39^ and, consequently, underestimating MOC_max_ can have significant implications for estimates of carbon deficit and potential accrual.

### Vegetation and management controls on carbon undersaturation across depths

**Fig. 2:**
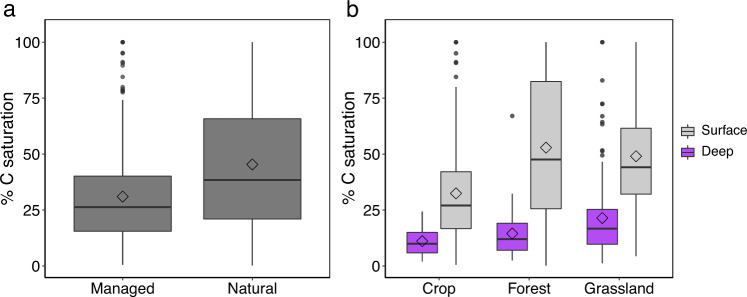
Percent mineralogical carbon saturation across ecosystems and soil depths. Percent mineralogical C saturation (%C saturation)—i.e., the proximity of a soil to its mineralogical carbon capacity (MOC_max_)—was calculated for each measurement in our observational synthesis. **a** %C saturation grouped by managed (*n* = 573) and natural (*n* = 862) ecosystems across depths (see “Methods”). **b** %C saturation by ecosystem (cropland, forest, and grassland) and depth (surface < 30 cm and deep > 30 cm). Only categories with >5 observations for both surface (cropland *n* = 425; forest *n* = 242; grassland *n* = 355) and deep (cropland *n* = 21; forest *n* = 53; grassland *n* = 136) soils are shown. Box plots indicate the medians (horizontal lines), 1st and 3rd quartiles (boxes), 1.5× interquartile range (whiskers), and means (diamonds).

Our analysis suggests that many soils are substantially below their mineralogical capacity (Fig. 1). This mineralogical undersaturation may be attributed to environmental (e.g., climate and plant C inputs) limitations on MOC storage and decomposition, management practices that result in MOC losses, or both. To explore where and why soils contain less MOC than they could based on their mineralogy alone, we calculated the mineralogical % C saturation for a given soil as the ratio of observed MOC to MOC_max_ (see “Methods”; Supplementary Fig. 10). Across all sites, soils averaged 40 ± 2% C saturation (±95% confidence interval of the mean). Land-use change and/or poor management practices that degrade soils can decrease MOC, a result evident in the shift of C saturation distributions from less-managed to more intensively managed ecosystems (Fig. 2a). The former (namely, grasslands and forests), which we refer to here as natural ecosystems for brevity, had on average higher levels of C saturation than did agricultural systems (*p* < 0.0001; Fig. 2a). Indeed, whereas soils in natural ecosystems averaged 46 ± 3% C saturation, agricultural systems averaged only 31 ± 2% C saturation. This contrast suggests that restoring degraded or intensively managed lands could push their soils towards higher C saturation levels (i.e., their highest attainable MOC given the site-specific environmental factors that limit stocks below MOC_max_) and allow them to serve as a substantial C sink.

We also observed that deeper soils were further from C saturation than were surface soils (*p* < 0.0001; Fig. 2b). On average, surface soils (0–30 cm) were 43 ± 2% C saturated, as compared to 19 ± 3% C saturation for deeper soils (30–120 cm). This difference is likely due, in part, to lower rates of C inputs with depth^40,41^. The location and type of C inputs are also important, as a higher proportion of plant C is retained in SOC from belowground root inputs than from aboveground litter inputs^42–44^. We may then expect a difference in C saturation levels between grasslands and forests, which have different root depth distributions^45^. Indeed, although there was no significant difference in the level of saturation between grasslands and forests in surface soils (0–30 cm), grasslands were closer to C saturation in deeper horizons (30–120 cm) than forests were (*p* < 0.005; Fig. 2b). This pattern confirms that root profiles and rates of C inputs influence the achieved MOC levels. This is an additional reason to expect that deeper-rooted vegetation may be more effective in sequestering C, and should thus be examined for restoring degraded lands and selecting cover crops^41,46^.

### Climate controls on mineral-associated organic carbon

**Fig. 3:**
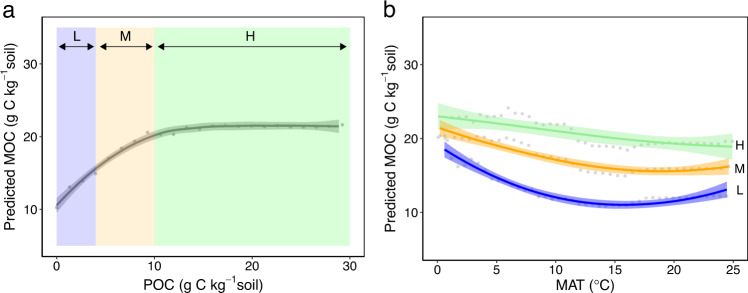
Data-driven predictive relationships of mineral-associated organic carbon as a function of individual controlling factors and interactions. A random forest model was used to disentangle the significance and emergent relationships of individual covariates, using partial dependence relationships that describe the marginal effect of each variable on the predicted mineral-associated carbon (MOC) response (see “Methods”). **a** Predicted MOC as a function of particulate organic C (POC). For each plot, the points show random forest model predictions; best fit lines and shading show 99% confidence intervals on each trend. **b** Predicted MOC as a function of mean annual temperature (MAT), conditional on three distinct subsets of POC—namely, low (L), medium (M), and high (H) POC corresponding to unsaturated to saturated regimes. The breakpoints were selected as the median and third quartile of the POC distribution, which also correspond to approximate saturation thresholds in the partial dependence plot of POC alone.

Climate also affects the achieved MOC in soil and its departure from saturation. However, MOC does not vary linearly with extrinsic factors such as temperature, but rather its response is governed by ‘pedogenic thresholds’ and nonlinearities^47^. We thus used a machine learning approach to determine the significance of key covariates and reveal emergent relationships of each variable, as well as variable interactions, on MOC (i.e., partial dependence plots; see “Methods”). This approach illuminates not only the overall sensitivity of MOC to individual variables but also the conditional relationship and rate of change with respect to a given variable (i.e., the partial derivative), without imposing a particular (e.g., linear) relationship. Specifically, we optimized a random forest (RF) model using key environmental covariates (see “Methods”; *R*^2^ = 0.60, Supplementary Fig. 11). We then calculated partial dependence relationships of MOC as a function of each variable alone (Supplementary Fig. 12), as well as conditional on interactions between variables (Fig. 3).

Clear predictive relationships emerged for MOC as a function of each variable. We found a linear partial dependence relationship between MOC and CS (Supplementary Fig. 12a), which can be attributed to an underlying increase in total mineral surface area and pore space^22,48^. This result supports studies that find correlations between MOC and CS, but illustrates the importance of controlling for the variability introduced by environmental covariates. With POC, we observed a saturating relationship in MOC (Fig. 3a), corresponding to an increase in C loading up to an effective saturation of mineral surfaces^48,49^. Although not all of this POC may be able to sorb onto mineral surfaces, it emerged as a significant variable in our analysis (Supplementary Figs. 11 and 12), likely as an indicator of rapidly decomposing material and the production of microbial necromass and dissolved organic carbon. In contrast, machine learning predictions of the conditional relationship between MOC and aboveground litter inputs showed a weaker dependence, consistent with other findings that aboveground productivity and litterfall are often not good proxies for carbon inputs to mineral soils^42^.

We observed a decline in MOC with MAT (Fig. 3b and Supplementary Fig. 12c) which is consistent with increased relative desorption with increasing temperatures^50,51^, and suggests a potential global vulnerability of MOC to warming. This decline with MAT also emerged across different ranges of POC, but interestingly, with significant differences in magnitude (Fig. 3b); low POC regimes (which are furthest from MOC saturation; Fig. 3a) exhibited greater temperature sensitivities compared to high POC regimes (soils closer to saturation; Fig. 3a). Distinct temperature-dependence regimes of saturating sorption curves are corroborated by theory (Supplementary Fig. 6) and experiments^52^. Specifically, when available carbon concentrations are low, MOC depends on the equilibrium constant ${K}_{{{{{{\rm{eq}}}}}}}=f(T)$ (i.e., the ratio of adsorption to desorption rate constants; Supplementary Fig. 6) which is a strong function of temperature, whereas when concentrations are high, MOC approaches ${{{{{\rm{MOC}}}}}}_{{{\max}}}{\,}\ne {\,}f(T)$ and hence exhibits a weaker temperature dependence. Thus, our findings suggest that restoring degraded soils towards their mineralogical capacity through improved land management may not only contribute to carbon sequestration efforts^53^ but also impart a greater resiliency of soils to future warming.

**Fig. 4:**
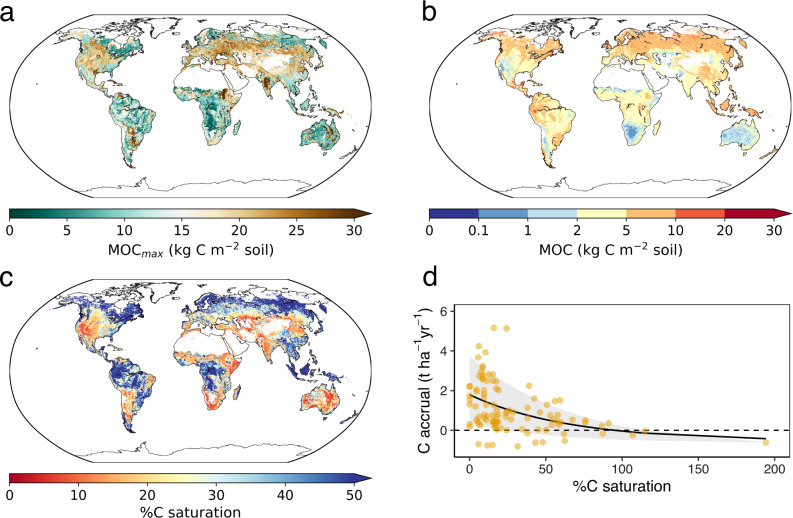
Global soil mineralogical carbon capacity, mineral-associated carbon stocks, and potential for carbon accrual. **a** Mineralogical carbon capacity (MOC_max_; kg C m^−2^ soil), **b** mineral-associated organic carbon (MOC; kg C m^−2^ soil), and **c** percent mineralogical carbon saturation (%C saturation) in topsoil (0–30 cm) at 0.5° resolution, excluding tundra, peatlands, and deserts. MOC_max_ was estimated using our data-derived relationships with the amount and type of mineral. MOC was predicted using a random forest model trained on our observational dataset. %C saturation was calculated as MOC/MOC_max_. Low %C saturation corresponds to a greater C deficit, and therefore highlights regions where targeted soil management could lead to higher C sequestration. **d** Carbon accrual in topsoil (t ha^−1^ yr^−1^; mean depth = 29 cm) as a function of %C saturation across sites, based on our global synthesis of studies that measured C accrual following management interventions (*n* = 103). Nonlinear fit depicts asymptotic regression model with shaded areas representing 10th and 90th quantiles.

Finally, we leveraged a machine learning approach to predict current MOC globally, excluding tundra, peatlands, and deserts (see “Methods”; *R*^2^ = 0.79, Supplementary Figs. 13 and 14). We estimated that MOC stocks total 899 Pg C (5–95% range: 668, 1074 Pg C) to a depth of 1 m, with 448 Pg C (296, 536) in topsoils (0–30 cm) and 451 Pg C (372, 538) in subsoils (30–100 cm) (Fig. 4 and Supplementary Fig. 15; Supplementary Tables 1 and 2). Globally, MOC made up a smaller proportion of total SOC in topsoils (0.66 ± 0.13; mean ± s.d.) compared to in subsoils (0.70 ± 0.17) (0.69 ± 0.15 to a depth of 1 m; Supplementary Fig. 16). Soils had a smaller proportion of MOC to SOC in boreal regions than in tropical and temperate regions, suggesting a larger proportion of non-protected carbon (i.e., POC) at high latitudes that may be vulnerable to warming^54^ (Supplementary Discussion). Our global estimates of MOC stocks, and as a fraction of total SOC, are to our knowledge the first such spatially- and depth-resolved data products, providing a crucial link for understanding soil carbon vulnerability, benchmarking soil carbon models^17^, and assessing the mineralogical potential for carbon sequestration globally.

### Global soil mineralogical carbon capacity

We used the same classification of mineral types from our observational synthesis to map high- and low-activity minerals (HM and LM, respectively; see “Methods”, Supplementary Fig. 14). Together with a global map of CS and our derived relationships for MOC_max_ in HM and LM soils, we calculated the corresponding MOC_max_ globally (Fig. 4 and Supplementary Fig. 17). This spatially explicit and depth-resolved global product provides insights into the capacity of the world’s soils to store MOC. Globally, we estimate that the soil mineralogical C capacity is 1443 ± 141 Pg C and 3153 ± 312 Pg C in topsoils and subsoils, respectively, totaling 4596 Pg C ± 453 Pg C to a depth of 1 m (excluding tundra, peatlands, and deserts; Supplementary Table 1). Many sites in temperate and subequatorial zones show the greatest mineralogical C deficit (MOC_max_ minus MOC), and thus potential for additional MOC storage, in part because of the prevalence of agricultural soils there (Fig. 4c and Supplementary Figs. 18–20). Indeed, we estimate that the mineralogical C deficit of croplands is 184 Pg C (5–95% range: 148, 225) and 509 Pg C (439, 569) in topsoils and subsoils, respectively (Supplementary Table 1). While reaching MOC_max_ is difficult and strongly limited by climate (Fig. 3), if these soils achieved the average %C saturation levels of natural lands (Fig. 2), this potential sequestration would total nearly 104 Pg C to a depth of 1 m (62 and 42 Pg C in topsoils and subsoils, respectively) (Supplementary Table 1). Grazing lands also show a large mineralogical C deficit covering a large expanse globally, but will require tailored strategies for soil C restoration, especially in arid and semi-arid regions that are additionally limited by climate (e.g., the Southwestern United States and Ustyurt Plateau). Our maps reveal hotspots of mineralogical C deficit, often associated with long-standing cropping and grazing lands (Fig. 4 and Supplementary Figs. 20 and 21), and highlight priority regions for soil C restoration efforts.

Current MOC stocks correspond to global average C saturation levels of 42% (5–95% range: 24%, 61%) and 21% (14%, 30%) in topsoils and subsoils, respectively (Fig. 4c and Supplementary Figs. 18 and 19; Supplementary Table 2). However, as explained earlier, reaching C saturation is not necessarily feasible (given climate limitations, for example) or a recommended target. Rather, the potential lies initially in restoring the natural %C saturation in managed areas (Supplementary Figs. 18–22; Supplementary Table 1) and, especially, those furthest from %C saturation (Supplementary Fig. 9; Supplementary Discussion). We identify geographic locations with the most promise (namely, temperate and subequatorial zones in the northern hemisphere; Fig. 4c) and highlight the potential benefit of practices that sequester C deeper in the soil profile, where soil minerals are further from %C saturation (Supplementary Figs. 18–20). In addition to practices that leverage deeper-rooted vegetation^41^, recent studies have indicated that new approaches to deep ploughing^55^ or other practices resulting in topsoil-subsoil flipping^56^ may lead to considerable overall C sequestration, although the cost and full impact of such practices on other soil functions needs to be better understood.

In addition to informing locations for targeted restoration and carbon sequestration, our data and results are also an essential input for Earth system models (ESMs) that seek to represent soil mineral-organic associations explicitly—directly informing the mineralogical C capacity and constraining the proportion of total SOC that is mineral-associated (Supplementary Figs. 16 and 17)^17^. Our global estimate of MOC_max_ constitutes an effective potential for sorption on minerals, unlike that derived from laboratory DOC sorption experiments alone^57,58^. Laboratory assays tend to underestimate maximum sorption potential, especially if they use native soil which already has some carbon loading on mineral surfaces^57,58^. Moreover, microbial necromass and residues contribute substantially to MOC measured in the field^59^, and thus, our estimates have the potential to uniquely improve model parameterizations beyond those obtained from laboratory measurements.

### Carbon sequestration implications and future perspectives

Geographic regions with higher C deficit can be used to inform soil C sequestration efforts, such as the “4 per 1000” initiative^60,61^ and other nature-based climate solutions^62,63^. Soils with higher deficit (Supplementary Fig. 20), and thus lower %C saturation (Fig. 4c, Supplementary Fig. 18), may provide greater C sequestration efficacy (Supplementary Fig. 9), through C accumulation rates that are larger and can be sustained for years to decades until a new steady-state is reached^39^. We synthesized studies that measured C accrual rates following management interventions intended to promote C storage (*n* = 103, ranging from 2 to 65 years; Supplementary Figs. 23 and 24) and found that soils furthest from their mineralogical capacity (i.e., lowest %C saturation) achieve the highest rates of C accrual (Fig. 4d). In fact, carbon accrual rates were on average three times higher in soils from sites with <10% C saturation compared to sites with 50% C saturation (Fig. 4d). This result is critical for identifying global regions that, in addition to a significant capacity for C storage, may also provide greater C sequestration efficacy (Supplementary Fig. 9; Supplementary Discussion). Indeed, many of these potential sequestration hotspots are located in extensive cropping regions (e.g., Midwestern United States and India; Fig. 4c and Supplementary Figs. 21 and 22) that could be incentivized to sequester C, with additional co-benefits for soil productivity and food security^64,65^.

Estimates of global potential soil C sequestration rates vary considerably, but recent reviews suggest the annual technical potential of individual practices (e.g., no-till agriculture and agro-forestry) applied globally is on the order of 2–5 Pg CO_2_ yr^−1^ (0.5–1.4 Pg C yr^−1^)^62,66,67^; implementation potentials given economic considerations fall at the lower end of this range^68^, while nutrient limitations may further limit achievable sequestration^69,70^. While the implementation of soil management interventions across the world’s >500 million active farms poses formidable socioeconomic challenges^71,72^, actionable valuation of soil C accumulation may incentivize restorative soil management in targeted areas^73,74^, with enduring co-benefits for soil productivity^64,65^. Despite the many challenges of such interventions, if the average C accrual rates from our synthesis (Fig. 4d) were achieved globally over all croplands (Supplementary Fig. 22), we estimate that soil C sequestration efforts could store over 1 Pg C yr^−1^ given current stocks, on par with the bottom-up estimates of soil C sequestration potential for agricultural lands^62,66,67^. We differ, however, in the duration that such carbon uptake rates could be maintained. Effective C sequestration would push soils towards higher %C saturation (Fig. 4d), thereby lowering their C sequestration efficiency over time.

While many soil C sequestration initiatives are centered on improved management of current croplands^60,61^ (which often show more modest C accrual rates and %C saturation; Supplementary Figs. 20–22 and 23b), the potential for soil C sequestration is relevant across nature-based climate solutions more broadly^62,63^. For example, there are growing efforts in quantifying the potential for C sequestration on abandoned pastures and croplands^75–79^, as well as restoring degraded rangelands^80–84^. Though agricultural lands are still expanding in some parts of the world, nearly 90 million hectares of agricultural lands were abandoned between 1985-2005 in North America, Europe, Oceania, and South Asia^85^ (and up to 472 million hectares have been abandoned globally over the last few centuries^86^). Many of these lands are in regions with low %C saturation, where our synthesis suggests that revegetation and restoration interventions have the potential to achieve high rates of C accrual (Fig. 4d; Supplementary Figs. 23–25, Supplementary Table 4). Finally, we note that given the considerable departure of most current and abandoned agricultural lands from their respective mineralogical capacities (Figs. 1, 2, Supplementary Figs. 20–22) (following losses in soil C content^5^), incentives that encourage widespread soil C sequestration efforts are crucial for scaling and achieving mitigation targets.

Our study provides a spatially explicit global estimate of the mineralogical capacity of the world’s soils to store carbon and an improved understanding of the factors—natural and managed—that drive soils below their capacity. While MOC constitutes the majority (69%) of total SOC in the top meter of non-permafrost mineral soils (totaling 899 Pg C), we find that this represents less than half of the mineralogical carbon-storage capacity. The understanding of how climate and vegetation affect MOC stocks, and the pronounced undersaturation in managed lands, in particular, suggests that there is significant potential for restoring or enhancing MOC storage. Our estimate of MOC_max_ can be refined in future studies as scientists collect more data on additional mineral types, but already provides a data framework to inform ESMs, paleo reconstructions of soils, and policies for soil carbon sequestration. Long-term changes in C inputs may have lasting effects on MOC that are particularly pertinent for land management decisions that seek to sequester carbon^87^. Other soil modification options—e.g., adding more clay to existing sandy soils^88^—to sequester more MOC may also be plausible, but require substantial research to investigate the gains, co-benefits, and potential consequences. Our findings shed light on potential responses and vulnerabilities of MOC to novel natural or managed conditions and emphasize that it is critical to consider the whole system when comparing policies and practices.

## Methods

### Observational synthesis of mineral-associated carbon

In the present study, we aimed to quantify the maximum capacity of soils to stabilize mineral-associated organic carbon as a function of soil mineralogy, and to resolve the underlying variability due to climate, vegetation, and management. We hypothesized that the maximum mineralogical capacity (MOC_max_) of soils is a function of the amount (% clay + silt; CS) and type (high- or low-activity; HM or LM, respectively) of mineral, while the achieved mineral-associated carbon (MOC) is additionally a function of climate, vegetation, and management:

1 $${{{{{\rm{MOC}}}}}}_{{{{{\mathrm{max }}}}}}={f}_{1}\left({{{{{\rm{mineral}}}}}}\;{{{{{\rm{ amount}}}}}},{{{{{\rm{mineral}}}}}}\; {{{{{\rm{type}}}}}}\right)$$

2 $${{{{{\rm{MOC}}}}}}={f}_{2}\left({{{{{\rm{MOC}}}}}}_{{{{{\rm{max }}}}}},{{{{{\rm{climate}}}}}},{{{{{\rm{vegetation}}}}}},{{{{{\rm{management}}}}}},\ldots \right)$$

Therefore, we performed an observational synthesis from studies that included MOC (as measured by size and density fractionation, retaining information about the size and density cutoffs), independent particulate organic carbon (POC) and soil organic carbon (SOC) measurements, as well as auxiliary data that included mean annual temperature (MAT), mean annual precipitation (MAP), clay and silt (CS), mineral type and soil order, vegetation type and carbon inputs, total, particulate, and mineral-associated nitrogen, and a variety of mineralogical soil properties (e.g., cation exchange capacity, iron, and aluminum content, among others), where available. To locate studies, we searched Google Scholar for combinations of the keywords “soil carbon”, “mineral-associated”, “clay”, “silt”, “size fractionation”, “density fractionation”, and “carbon saturation”. We found that studies that measured MOC by both size and density fractionation (e.g., refs. ^89–91^) were consistent and within the limit of MOC_max_ for each mineral type, and that both methods concluded similar ranges of MOC to SOC ratios^10^ (Supplementary Fig. 1). These findings support the use of either fractionation method for the goals of our particular study.

The observational synthesis totalled 1144 mineral soil profiles from 78 studies that reported fractionation and bulk measurements of carbon and nitrogen across depths. Specifically, the dataset included: 359 (<2 μm; clay fraction only) and 1451 (<63 μm; clay and silt fraction) MOC measurements, 1107 POC measurements, and 1432 SOC measurements, as well as corresponding MAT, MAP, CS, soil order, mineral type, and vegetation type (see section “Defining and evaluating ecosystem categories” for classification details). We also collected other auxiliary soil properties, including dithionite- and oxalate-extractable iron (Fe_d_ and Fe_o_, respectively), oxalate-extractable aluminum (Al_ox_), cation exchange capacity (CEC), specific surface area (SSA), and total reserve bases (TRB), though these measurements were sparse across the collected studies. A complete list of studies and their location, climate, and vegetation is provided in Supplementary Table 3.

Studies in our synthesis spanned intensively managed (e.g., crop) to natural/less-managed (e.g., grassland, forest) ecosystems across diverse climates and soil types (Fig. 1 and Supplementary Fig. 2). We obtained a representative mix of MOC measurements from managed (*n* = 559 profiles) and natural (*n* = 585 profiles) lands. We focused on the current management status herein, but note that future studies may consider information on historical management, where available. Within natural lands, there was a greater representation of temperate ecosystems, as opposed to tropical and boreal ecosystems (Supplementary Figs. 2, 3, and 14)—especially when including the European LUCAS database^11,92^ (Supplementary Fig. 2b, d), but our model and results were robust to its exclusion. Furthermore, measurements in tundra and deserts were limited, highlighting the need for studies in these regions (Fig. 1c, Supplementary Figs. 2 and 3). Nevertheless, our observational synthesis spanned diverse climates and soil types: MAT ranged from −2.9 to 29 °C, MAP from 79 to 3806 mm yr^−1^, and CS from 1.5 to 100%, across different mineral and vegetation types (Fig. 1b, c and Supplementary Fig. 2). Indeed, our synthesis spanned the majority of the climate-edaphic covariate space encompassed by the world’s soils in WoSIS (World Soil Information Service)^93^ (Supplementary Fig. 3a, b). We further illustrated the global representativeness of our synthesis within a multi-dimensional covariate space using a principal component analysis (PCA) and comparing to randomly sampled WoSIS profiles (Supplementary Fig. 3c). We note that even WoSIS soil profiles showed a greater representation in temperate ecosystems compared to tropical and boreal ecosystems^94^ (Supplementary Fig. 3b), further highlighting the need for additional measurements in these regions.

### Defining and evaluating ecosystem categories

Here we classified soils into two broad categories of mineral type and activity; namely, low-activity minerals (LM) and high-activity minerals (HM). We used soil order from each individual study and, when provided, details on clay composition to categorize the soil of each site. Phyllosilicate clay minerals—composed of tetrahedral silicate sheets and octahedral hydroxide sheets—are composed of either 1:1 (one tetrahedral and one octahedral sheet) or 2:1 (one octahedral sheet sandwiched between two tetrahedral sheets) clays. 1:1 clays are prevalent in kaolinitic soils and generally have a lower SSA^14,28,29,95^; therefore, such soils generally exhibit a lower capacity to stabilize carbon and can be characterized as LM. In contrast, 2:1 clays are present in smectitic (e.g., montmorillonitic) and illitic soils and can be characterized as HM, on account of their high SSA^10,14,28,29^. Finally, soils with amorphous, poorly crystalline minerals and mineraloids (e.g., allophane) also depicted high stabilization (described in greater detail below) and were grouped with HM.

Soil orders (USDA taxonomy) represented in this study include Alfisols, Andisols, Aridisols, Entisols, Gelisols, Inceptisols, Mollisols, Oxisols, Spodosols, Ultisols, and Vertisols; we excluded organic soils in wetlands and peat, and, thus, Histosols were not considered. When detailed information on mineral composition (e.g., the dominant presence of 2:1 vs. 1:1 clays) was not explicitly stated in the original studies, the following categorization was used based on the primary mineral composition by soil order^31^: Oxisols and Ultisols at all depths were LM; and Alfisols, Aridisols, Gelisols, Inceptisols, Mollisols, Spodosols, and Vertisols were HM. Andisols and Entisols were HM in the topsoil (0–30 cm) and LM in the subsoil (30–100 cm). We note that, in future studies, allophanic soils with large amounts of amorphous minerals (i.e., Andisols) could be treated as a third category due to their propensity to form stable Al- and Fe-complexes and accumulate SOC^14,32^; however, we did not observe a significant difference in their MOC as compared to HM soils in our synthesis. Furthermore, there are complexities, including the amount and type of metal oxides and hydroxides, that can make the distinction between low- and high-activity minerals difficult in certain soils^29,96^ (and can also complicate the comparison of soil clays and “reference” or synthetic clays^97^), the classification of LM and HM soils has been widely used and supported^23,28,29^ and emerges from our data globally (Fig. 1 and Supplementary Figs. 7 and 8). Indeed, the MOC patterns were consistent within our classifications and, thus, these categories were deemed sufficient for the scope of this global-scale study.

We retained the classification of vegetation type, and all details on management practices or lack thereof, reported in the original studies and assigned one of eight land cover types (Fig. 1b–c). Land cover types included boreal forest, temperate forest, tropical forest, grassland, savanna, shrubland, and cropland (Supplementary Fig. 14). To further compare managed and natural lands, we grouped sites into broad categories of forests, grasslands (including savannas), and croplands. We observed stark differences between natural or less-managed ecosystems (namely, forest and grassland) and more intensively managed (crop) ecosystems (Fig. 2). We calculated the %C saturation of soils (as depicted in Supplementary Fig. 10) to compare ecosystems and management practices.

### Boundary line analyses

To estimate the mineralogical carbon capacity (MOC_max_) as a function of soil mineralogy, we conducted a boundary line analysis across the full range of clay and silt (CS) for each mineral category (LM and HM, see section “Defining and evaluating ecosystem categories”; Supplementary Fig. 26). Specifically, we explored the relationship of MOC (y-axis; g C kg^−1^ soil) as a function of CS (*x*-axis; %) and fit the 95th quantile for natural ecosystems of each mineral type to obtain maximum boundary-line slopes for HM and LM (Fig. 1; quantile sensitivity analysis in Supplementary Fig. 7). We chose the 95th quantile as a conservative approximation of this boundary line, given existing experimental uncertainty of individual observations (i.e., total % recovery of C from size fractionations ranged from ~80 to 120% among studies). We used MOC observations from clay and silt (<63 μm) size classes to derive MOC_max_ (Fig. 1) and note that our results were robust to the choice of particle size (e.g., <20 μm or <63 μm; Supplementary Fig. 5). The 63 μm silt size threshold chosen is also that used in global datasets and mapped products of soil clay and silt^4,98^. We also note that our estimates of MOC_max_ for HM and LM were robust across topsoils and subsoils. Both topsoils and subsoils approached MOC_max_ (Fig. 2b), and thus, a separate MOC_max_ for each depth was not theoretically or empirically warranted. While it is more difficult to reach MOC_max_ in subsoils due to lower carbon inputs, MOC_max_ is an intrinsic property of the soil mineralogy and constitutes a theoretical upper limit that is independent of depth; indeed, this depth-independence is important for capturing MOC stocks and ages in process-based soil carbon models^99,100^. Our estimates of MOC_max_ for HM and LM were also robust across vegetation types (Supplementary Fig. 8), which corroborates studies showing that microbial processing and reactive minerals act as effective filters that lessen chemical differences in organic matter inputs^59,101^. Theory and models further support this finding and suggest that, while litter quality may affect the approach of a given soil to its mineralogical capacity, the value of MOC_max_ is independent of vegetation type^28,102^. Regressions for both mineral types were fit with a forced intercept through the origin, since, by definition, no clay- and silt-associated organic carbon (i.e., MOC) can exist without clay and silt minerals present. While some size fractionation studies report MOC in sandy soils with very low amounts of clay and silt minerals^92^, further exploration is needed to understand the relative contribution of fine POC and DOC in such soils^16^.

### Statistical modeling and predictive relationships

Due to potential nonlinear relationships between MOC and environmental variables, we used an ensemble machine learning method—namely, Random Forest (RF)—to identify key predictors and their effects on MOC. This method has been shown to reduce over-fitting to a training dataset compared to other machine learning methods, and does not suffer from multi-collinearity (i.e., the linear dependence among predictor variables), as do multiple regression analyses^103^. We used the RandomForest package in R for RF analyses^103,104^. We used an ensemble of 300 independently trained RF models (using a 75–25% train-test split; 400 decision trees each) with bootstrapped sampling to robustly assess model performance^105^—mean absolute error (MAE) and mean-squared error (MSE) to quantify the model error and *R*^2^ to estimate the proportion of variance in MOC explained by the model (Supplementary Fig. 11). Our aim was to: (i) elucidate key variables by assessing their relative contribution to the MSE of test-set predictions when removed or permuted, (ii) calculate the total variance explained by selected predictors, and (iii) predict the response of MOC to a given variable while controlling for all other variables.

RF is often used to rank the importance of each variable in a regression through permutations of the data^103^, as implemented herein. After preliminary investigation to select the most important variables, while also considering variables that were measured in sufficient studies to retain the greatest number of complete observations, we identified CS, MAT, MAP, and POC as key continuous predictors and vegetation (land cover) and mineral type as key categorical predictors (Supplementary Fig. 11; *R*^2^ = 0.60 ± 0.06). Specifically, variables were ranked from most to least important, where the most important variables showed the highest increase in MSE of test set predictions when permuted in the model.

We then investigated the underlying relationship of MOC to key variables (Fig. 3 and Supplementary Fig. 12). RF allowed us to explore the effect of each individual variable on MOC, by integrating the predicted response over the contribution of all other variables (i.e., partial dependence plot)^106^. That is, for a variable *x*_*s*_ and complement set of all other variables *x*_*C*_, the general model function is $f\left({x}_{s},{x}_{C}\right)$ and depends on all input variables. The partial dependence function of *x*_*s*_, denoted ${\hat{f}}_{{x}_{s}}\left({x}_{s}\right)$, then describes the marginal effect of *x*_*s*_ on the prediction, and is estimated as:

3 $${\hat{f}}_{{x}_{s}}\left({x}_{s}\right)=\frac{1}{n}\mathop{\sum }\limits_{i=1}^{n}\,f\left({x}_{s},{{x}_{C}}^{(i)}\right)$$

This was calculated for each predictor variable over the full range of the other predictor variables (Supplementary Fig. 12), and also over relevant subsets of the other predictor variables to explore conditional partial dependencies and visualize two-way variable interactions (e.g., Fig. 3). For the case of MAT and POC, we explored the temperature sensitivity of MOC (i.e., the partial dependence relationship with MAT) conditional on low (L), medium (M), and high (H) values of POC that corresponded to different saturation regimes—unsaturated, medium, and saturated (Fig. 3a). The breakpoints for these three regimes were selected as the median and third quartile of the POC distribution (namely, 4 and 10 g C kg^−1^ soil, respectively), which also correspond to approximate saturation thresholds in the partial dependence plot of POC (Fig. 3a).

Our approach permits us to disentangle the role of each controlling variable and two-way interactions on the response variable of interest (here MOC) and uncovers emergent (linear, monotonic, or nonlinear) relationships, providing unique insights for MOC response to future climate and management interventions (e.g., increasing POC or CS). For comparison, we also repeated the above analysis with a generalized linear model (GLM) and find qualitatively similar, though linearized, trends between MOC and the individual environmental predictors.

### Global mineral-associated carbon predictions and uncertainty

Following the exploration of underlying climatic and edaphic properties, we trained a RF model on our observational synthesis to predict MOC globally. Specifically, we trained the RF model to predict MOC from MAT, MAP, CS, SOC, vegetation type, and mineral type (Supplementary Fig. 26). We note that a global map of POC values did not exist to be used as an explanatory variable, as was used in the attribution analyses (Supplementary Fig. 11); rather, global POC values can be derived as a product of this study.

The Scikit-learn Library in the Python environment was used for global predictions and Matplotlib Basemap for global mapping. We again used an ensemble of 300 independently trained RF models (using a 75–25% train-test split; 400 decision trees each) with bootstrapped sampling to rigorously assess model performance^105^. For these RF models that included SOC, a comparison of predicted and observed MOC on the independent test datasets across the ensemble yielded an *R*^2^ = 0.79 ± 0.05 (mean ± s.d.; Supplementary Fig. 13). We note that, while the predictability of SOC is often lower across large global datasets^4,107^, the predictability of MOC is generally high given readily measured SOC, climate, and edaphic variables (e.g., see refs. ^11,92^ on a regional scale). Indeed, the *R*^2^ was consistently high across the ensemble cross-validation (Supplementary Fig. 13b). We also performed a spatially buffered leave-one-out cross-validation^108^ to ensure that spatial auto-correlation did not significantly compromise the RF model. Namely, for each leave-one-out test data point, all data within a given buffer radius (0–150 km) were excluded from the training set; i.e., only data points outside the buffer zone were used to train each RF model. *R*^2^ values did not vary with buffer radius (regression slope not statistically different from zero; *p* = 0.81) and were in agreement with the ensemble cross-validation approach (Supplementary Fig. 13c). Furthermore, we also used a hold-out (or block) cross-validation^109^ on the basis of soil order to assess extrapolation performance and confirm the robustness of our RF estimates across soil types—i.e., all soil profiles from a given soil order were completely withheld from the training dataset and then used to independently test the resulting RF model. Model performance was consistently high across the out-of-sample test datasets (*R*^2^ = 0.75 ± 0.11; mean ± s.d. across soil orders); only Andisols (which span ~1% of the ice-free land surface) were more difficult to predict when completely withheld from the training dataset (*R*^2^ = 0.40), and we encourage more measurements in these soils (see section “Defining and evaluating ecosystem categories”). Hold-out cross-validation on the basis of individual studies also confirmed the robustness of our RF estimates to additional data (e.g., our results were insensitive to adding the independent LUCAS dataset^92^; Supplementary Fig. 2). We rigorously demonstrate the RF model performance and provide uncertainty ranges for the 90% prediction intervals, derived from the 5th and 95th quantiles (Supplementary Fig. 13a).

The climatic and edaphic driver variables used for the global predictions were all re-gridded to 0.5° × 0.5° resolution (Supplementary Fig. 14). Mean annual temperature (MAT) was estimated from the CRU data set (version 3.10)^110^, and mean annual precipitation (MAP) from the GPCC dataset^111^, both as 30-year annual averages. Clay and silt (CS) content was obtained from the Harmonized World Soil Database (HWSD)^98^. Soil organic carbon (SOC) was obtained from both the HWSD and SoilGrids^4,98^, and also as the average of the two gridded data products^112^ (Supplementary Fig. 19 and Supplementary Table 2). Land cover (vegetation type) was obtained from the MODIS MCD12C1 product^113^, and the 16 categories were combined into 10 for consistency with those reported in the observations (Fig. 1b, c and Supplementary Fig. 14 for details). The primary mineral type was calculated from global estimates of mineral composition^31^ (Supplementary Fig. 14; see section “Estimating the global soil mineralogical carbon capacity”). We focused our analysis on mineral soils, and thus excluded grid cells containing a majority of organic soils (i.e., >50% Histosols and Gelisols)^114,115^. Tundra and deserts were also excluded, due to data limitations in these regions (Supplementary Figs. 2 and 14). We used two depth intervals for SOC, CS, and primary mineral type, namely topsoil (0–30 cm) and subsoil (30–100 cm).

The predictive RF model (*R*^2^ = 0.79 ± 0.05; Supplementary Fig. 13), trained on the observational data, was then used to predict MOC globally for each depth interval to 1 m (Supplementary Fig. 15; Supplementary Tables 1 and 2 summarize results for all SOC data products). We include MOC uncertainty ranges for the 90% prediction intervals (5th and 95th quantiles) globally (Supplementary Fig. 15; Supplementary Table 2). Interestingly, we note that the distributions of MOC and SOC (and %C saturation) from our globally distributed observational dataset most closely agreed with SOC and predicted MOC (and %C saturation) from the averaged data product (Supplementary Figs. 18 and 19; Supplementary Table 2), whereas the distribution of SOC (and %C saturation) from SoilGrids was higher and HWSD was substantially lower (Supplementary Fig. 19; Supplementary Table 2). We focus here on reporting results using the averaged SOC data product, as a conservative estimate of MOC stocks given the uncertainty and range between soil carbon data products, and note that more accurate estimates and consensus on current soil carbon stocks are critical for improved predictions^116^.

Leveraging our global MOC predictions, we calculated the proportion of SOC that is mineral-associated globally (Supplementary Fig. 16). Our global estimates of MOC/SOC ratios agree with those observed in our observational synthesis and confirm the dominant contribution of MOC to soil carbon at the global scale (Supplementary Figs. 1 and 16). Furthermore, our predictions suggest that high-latitudes have a lower fraction of MOC/SOC globally, and thus a higher fraction of particulate organic carbon (POC), but data limitations of mineral-associated carbon measurements at high-latitudes warrant further exploration of these patterns.

### Estimating the global soil mineralogical carbon capacity

To estimate the spatially explicit mineralogical carbon capacity (MOC_max_) globally, we used global data products of clay and silt content (CS), soil order and mineral composition, and bulk density (BD), together with our derived relationship for MOC_max_ (Eq. 1; Fig. 1). Global maps of % clay, % silt, and BD were obtained from the HWSD^98^. A map of soil mineral composition^31^ was used to generate a corresponding map of primary mineral type (Supplementary Fig. 14; see section “Defining and evaluating ecosystem categories”). Our focus here was on mineral soils, and thus, we excluded organic soils (grid cells with >50% Histosols and Gelisols) from the estimation of global MOC storage potential. Tundra and deserts were also excluded due to data limitations (Supplementary Figs. 2 and 14). We calculated CS content and applied our MOC_max_ relationship (Fig. 1; slope ± 90% confidence intervals) for each mineral type (HM and LM) globally to obtain MOC_max_ (gC kg^−1^ soil) at 0.5° × 0.5° resolution. For a global total (in Pg C; 1 Pg =  10^15^ g), we used BD to calculate MOC_max_ stocks (in kg C m^−2^ soil) and then summed over all grid cells containing mineral soils (Supplementary Tables 1, 2).

The mineralogical capacity MOC_max_ (Supplementary Fig. 17) was then compared to predicted MOC stocks (Supplementary Fig. 15) to calculate the mineralogical C deficit (by subtracting MOC from MOC_max_) and %C saturation (by dividing MOC/MOC_max_) globally (Fig. 4; Supplementary Figs. 18–20). We note that this limit is based on mineralogy alone, and many lands may be further limited by climate. Thus, in addition to the C deficit relative to the mineralogical capacity (MOC_max_), we also estimated the C deficit relative to an environmental limit calculated using the natural land average %C saturation (i.e., average MOC/MOC_max_) observed in our MOC synthesis at each depth (Fig. 2; i.e., 51 and 19% for topsoil and subsoil, respectively). These results are summarized in Supplementary Table 1. We note that our estimates of C deficit and %C saturation using SoilGrids are the most conservative for potential accrual, given their higher current SOC (and consequently MOC) stocks (Supplementary Table 2). However, our estimates using the averaged soil carbon data product agree with the distributions of MOC stocks and %C saturation in our observational synthesis (Supplementary Fig. 19; Supplementary Table 2), and provide a robust estimate that incorporates the range of current soil carbon stocks.

Our spatially explicit, depth-resolved global estimates highlight regions where larger C deficits (lower %C saturation) can be targeted for soil restoration and C sequestration efforts, especially on managed lands (Fig. 4c; Supplementary Figs. 21 and 22). While environmental conditions may make carbon accumulation more difficult in some regions than in others (Supplementary Fig. 12), this estimate constrains MOC_max_ for a given soil mineralogy and provides a starting point for areas to focus on management (Supplementary Discussion). Furthermore, while deep soils have the largest C deficit, surface soils in lands already dominated by management may be the most cost-effective areas on which to focus.

### Observational synthesis and analysis of carbon accrual

Here we aimed to quantify the ability of topsoils ranging in mineralogical carbon saturation to further accrue carbon. We hypothesized that soils further from their mineralogical capacity (i.e., lower MOC saturation) would accrue carbon faster than soils closer to their capacity. To this end, we performed an observational synthesis from manipulation and chronosequence studies that included initial and final carbon stocks or concentrations to calculate these stocks, bulk density, experimental duration, and edaphic properties; namely, clay and silt content, texture class or soil series to estimate these contents and soil classification or other information that would allow us to assign the soil to a high- or low-activity mineral type.

For each entry (a pair of initial and final carbon stocks), we calculated the maximum mineralogical capacity (MOC_max_) using the data-derived relationships (Fig. 1) with an amount (% clay + silt; CS) and type (high- or low-activity; HM or LM, respectively) of mineral in each soil layer and used respective bulk densities to calculate the layer MOC_max_ stocks (t C ha^−1^) which were then summed across the considered depth to obtain the profile MOC_max_ stock (t C  ha^−1^). In cases where initial and final soil profiles differed in clay and silt content, their average was used. However, if the difference resulted in these soil profiles falling into different texture classes, they were excluded from further analysis. Furthermore, to calculate initial and final stocks, we considered initial and final profiles that were taken at equal depths, which ranged between 10 and 40 cm across studies. Where necessary, initial and final SOC stocks were standardized to equal depths using the maximum depth of the shallower profile as a cutoff ranging between 30 and 39 cm (*n* = 5 profiles).

We calculated the mineralogical %C saturation as the initial MOC stock divided by the initial MOC_max_ stock. Because MOC was not measured in all accrual studies, we used the measured SOC and CS values to calculate the corresponding MOC using our random forest model trained on the MOC synthesis data with analogous SOC and CS predictors (*R*^2^ = 0.70 ± 0.06; using Scikit-Learn in Python, see section “Global mineral-associated carbon predictions and uncertainty”).

Carbon accrual (t C ha^−1^ y^−1^) was calculated as the difference between final and initial C stock divided by the time period between the repeated samplings (*n* = 32), the duration of the experiment in the case of a paired plot experimental design (*n* = 30), the age difference between two sites of a chronosequence (*n* = 24), the time span at which a regression across multiple samplings was used to obtain an average rate for a chronosequence (*n* = 10), or the time of soil formation after emergence of a SOC-free parent material (*n* = 7).

Accrual studies spanned crop, pasture, grassland, and forest ecosystems across different climates and soil types (Supplementary Table 4; Supplementary Fig. 23). We focused on studies in which carbon inputs were manipulated or land-use was altered in a way that carbon accrual could be expected. These included improved agriculture (cover cropping, no till, crop-livestock rotation, deep ploughing, grazing reduction), land use change (conversion of cropland to grassland or forest), doubled litter or wood input from the DIRT (Detrital Input and Removal Experiment) network, and natural revegetation and mine reclamation (formerly degraded lands or newly exposed land surfaces as a result of mining, landsliding, alluviation, or marine terrace uplift, where soil C accrual can be expected as a consequence of natural or human-accelerated revegetation and soil formation). We note that most studies did not explicitly quantify C inputs over time, especially those where changes in C inputs stemmed from downstream impacts on vegetation, and we strongly urge the reporting of C input estimates in future studies. After the initial compilation, profiles were excluded in which the studied soil depth was lower than 15 cm (*n* = 2) and in which organic amendments were applied annually, in an initial one-time application higher than 20 t ha^−1^ without a control plot or at unknown intensity (*n* = 11).

Our accrual synthesis included 103 observations from 34 studies, with depths ranging between 15 and 40 cm (mean  = 29 cm). The synthesis included soils with both high-activity minerals (*n* = 84), especially Entisols (*n* = 41) and Alfisols (*n* = 20), and low-activity minerals (*n* = 19), belonging to Oxisols (*n* = 10) and Ultisols (*n* = 9). There was a greater representation of temperate climates, highlighting the need for additional studies in poorly represented regions including tropical and boreal climates in future work. However, globally, our study suggests that the greatest potential for accrual (i.e., lowest %C saturation) often occurs in these temperate regions (Fig. 4b) and was thus a focus of our work.

While most soils in the C accrual synthesis fell below 100% C saturation, we note that MOC_max_ was derived as a conservative estimate from the 95th quantile of our MOC synthesis (Fig. 1; see section “Boundary line analyses”) and therefore, by definition, soils can fall near or above this maximum. Indeed, in our MOC synthesis, ~200% C saturation values were observed for soils with low values of MOC and MOC_max_. Of the 103 accrual measurements, one sandy soil (i.e., low MOC and MOC_max_) displayed seemingly high %C saturation, yet still within expected ranges (Fig. 4d). Furthermore, this soil was categorized as an Andisol, which could be treated as a third category in future studies (see section “Defining and evaluating ecosystem categories”), due to its propensity to form stable Al- and Fe-complexes and accumulate carbon^14^. Nevertheless, removing this individual measurement, or even all Andisols (*n* = 6) in the C accrual synthesis, did not change the fitted model.

Furthermore, we note that the reclaimed mine soils in our dataset constituted two major categories of abandonment and regeneration—those with and without topsoil application (Supplementary Fig. 23b). Mine reclamation that employs the application of topsoil (usually a 30 cm layer, originally excavated before mining and stored in stockpiles; *n* = 31) is, in terms of management procedures, comparable to the conversion of cropland (disturbed by tillage and/or erosion) to grassland or forest. Reclamation can involve some mechanical preparation of soil, application of fertilizer or other organic amendments, and planting vegetation. This reapplied topsoil is sampled as a baseline and subsequent sampling in the following years is used to monitor C accrual. In reclamation without topsoil application (*n* = 13), vegetation either spontaneously establishes itself or is planted directly into the parent material. Such conditions represent early stages of soil formation and are parallel to the restoration of soils heavily impacted by erosion that has led to the complete loss of topsoil with only the infertile parent material left behind. While mine soils are well-described systems suitable for the study of soil management, included in previous syntheses of soil carbon dynamics after land-use change^117,118^, the fitted model and observed negative trend in C accrual with %C saturation were robust to their exclusion (Supplementary Fig. 23c).

Statistical analyses were conducted using the ‘nls’ package in R to fit a nonlinear asymptotic relationship between carbon accrual rates and the mineralogical percent saturation of the respective soil profiles. Specifically, we used a three-parameter self-starting asymptotic regression model (SSAsymp) on the 103 rate measurements (*R*^2^ =  0.18; Fig. 4d). The same approach was also used to fit the 10th and 90th quantiles (Fig. 4d). There was substantial variability in observed accrual rates for a given %C saturation, due to differences in climate and land use across sites (Supplementary Fig. 23). However, most of this variability appeared in soils furthest from C saturation, where environmental and management factors also play a role in observed accrual rates. In soils closest to saturation, this variability was far lower as carbon accrual rates tended towards zero, illustrating the importance of the mineralogical limit. Our focus here was on constraining an average empirical rate of carbon accrual as a function of %C saturation and investigating whether soils furthest from C saturation achieve higher carbon accrual rates. We also note that the variability in C accrual rates was better explained by %C saturation (*R*^2^ = 0.18) than by initial SOC (*R*^2^ = 0.04-0.10; Supplementary Fig. 24), further demonstrating the relevance of the mineralogical limit.

We used the nonlinear asymptotic regression to provide a spatially explicit global estimate of potential C accrual rates in topsoils dominated by management (Supplementary Fig. 22). While such accrual rates would be difficult to achieve everywhere, our spatially explicit estimates of potential C accrual rates highlight priority regions for future soil restoration and C sequestration initiatives and provide an independent estimate of global soil C sequestration potential.

## Supplementary information

Supplementary Info

## Data Availability

The globally gridded maps of mineral-associated carbon and mineralogical carbon capacity derived in this study are freely available and archived at Zenodo (10.5281/zenodo.6539765). The observational syntheses (mineral-associated carbon and accrual measurements) in support of these findings are detailed in Supplementary Tables 3 and 4, and archived at Zenodo (10.5281/zenodo.5987415).

## Code availability

All code relating to this study is available from the corresponding author upon request, and is archived at Zenodo (10.5281/zenodo.6539765).
