# Supplementary material for: Global stocks and capacity of mineral-associated soil organic carbon
Source: Nat Commun. 2022 Jul 1;13:3797. doi: 10.1038/s41467-022-31540-9 (PMC9249731; doi:10.1038/s41467-022-31540-9)
Supplement: Supplementary file 1 — Supplementary Info [file 41467_2022_31540_MOESM1_ESM.pdf]

Supplementary Materials for

Global stocks and capacity of mineral-associated soil organic carbon

Katerina Georgiou, Robert B. Jackson, Olga Vindušková, Rose Z. Abramoff, Anders Ahlström, Wenting Feng, Jennifer W. Harden, Adam F. A. Pellegrini, H. Wayne Polley, J. L. Soong, William J. Riley, Margaret S. Torn

Correspondence to: georgiou1@llnl.gov

This file includes:

Supplementary Discussion ………………………………………………………………… 2
Supplementary Figures 1 to 26 ……………………………………………………………… 4
Supplementary Tables 1 to 4 ……………………………………………………………… 30
Supplementary References ………………………………………………………………… 42

Supplementary Discussion

Soil organic carbon (SOC) is incredibly complex and heterogeneous[1,2], and therefore, effectively managing SOC stocks to address short- and long-term climate mitigation targets requires knowledge of key underlying components of SOC and their response to perturbations[3–6]. Fractionation methods separating SOC into mineral-associated (MOC) and particulate (POC) components have been widely proposed as an appropriate level of detail and a first step towards understanding broader-scale patterns[5,6]. MOC has been shown to have distinct properties (e.g., temperature sensitivity) compared to POC[6,7] and, therefore, knowing the distribution of soil carbon between these two pools has important implications for understanding the potential vulnerability of soils to future disturbance (e.g., warming)[8–10]. Since MOC and POC form, persist, and function in distinct ways, separating them is an important step towards designing effective mitigation pathways that have the potential to improve both soil carbon sequestration and functioning[4–6,11–14]. Ultimately, sequestration strategies should target MOC and/or POC accumulation taking into account ecosystem properties such as mineralogical saturation level (see Fig. 4), nutrient availability, and microbial community composition, as well as economic considerations and soil fertility goals[6,11,15–17].

In the present study, we focused on the limits and controls of MOC; i.e., the intrinsic ability of soils to store carbon through the formation of mineral-organic associations. There are clear theoretical considerations (e.g., leveraging sorption kinetics; Supplementary Fig. 6) to suggest that the potential for associations with mineral surfaces is effectively finite and therefore a saturation capacity for MOC can be calculated[6,18–21] (i.e., MOC_{max}, as presented herein). In contrast, POC (and SOC as a consequence) does not have this same expectation of a saturation capacity (Supplementary Fig. 4; also proposed conceptually by Lavallee et al.[6] and Castellano et al.[19], and using model-predicted values in Cotrufo et al.[5]). Furthermore, mineral-organic associations can limit microbial access to otherwise decomposable substrates[22,23] and, consequently, many studies have suggested that increasing MOC may be a key to lasting carbon sequestration in soils[6].

While MOC is generally very old throughout the soil profile, and significantly older than POC at the same depth[6,24–26], there can be a dynamic component of this pool that is susceptible to short-

term disturbances[27–32]. At equilibrium, this dynamic component may desorb and resorb without being decomposed, depending on microbial energy limitations and spatial separation[2,23,33–35]. In such a case, even this dynamic component can be old. Indeed, process-based models have shown that microbial energy limitations are a key factor in driving emergent carbon ages[35,36], which is supported by the observed differences between carbon ages in topsoil and subsoil horizons. Following a perturbation, however, MOC will ultimately tend towards a new equilibrium with the mineral matrix given the existing environmental conditions.

Indeed, carbon tracer studies have shown that even old carbon pools (e.g., at depth and/or mineral-associated) can undergo changes over timescales on the order of years to decades and should not be overlooked in the context of carbon and nitrogen balances. For example, Balesdent et al.[37] showed that over two decades, subsoil horizons contained >20% of the recently accumulated carbon within the top meter of soil, and many others have shown the potential for MOC accumulation over years to decades[27–29,38]. While the vulnerability of newly-accumulated MOC or POC to future perturbations remains an important and open question (further highlighting the need for studies that explicitly separate their responses to global change factors[6]), sustained management strategies and carbon monitoring should be used to promote and preserve increases in soil carbon over time and focus efforts on degraded lands that are furthest from their mineralogical limits[4,39]. To this end, a better understanding of the distribution, accrual, and limits of soil carbon pools across depths globally, as presented herein for MOC, is critical for informing mitigation strategies and locations[4–6].

Furthermore, given the importance of MOC and POC[6,40], recent work has called for their study across spatial scales[5,41] and their explicit incorporation in process-based soil carbon models[42–44]. However, current process-based models at continental to global-scales show a large spread in the amount of carbon contained in a mineral-associated, protected form (e.g., see Wieder et al.[45]). These discrepancies have been identified as important targets for benchmarking and refining process-based soil carbon models[43,44,46], and our spatially-explicit global estimates are a key advance towards these efforts.

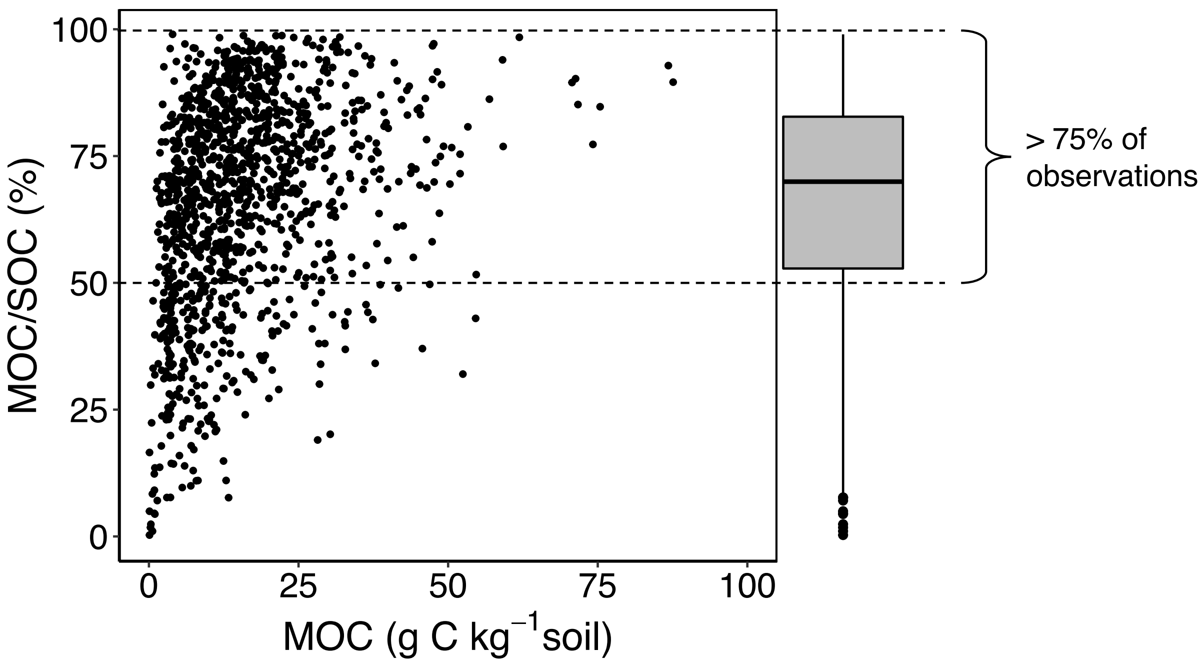

Supplementary Figure 1 | Mineral-associated organic carbon accounts for the majority of total soil organic carbon in our observational synthesis. Ratio of mineral-associated organic carbon (MOC) to total soil organic carbon (SOC) as a function of MOC across all biomes and climates. The box plot shows the marginal distribution of MOC/SOC and indicates the median (horizontal line), 1st and 3rd quartiles (box), and 1.5 × interquartile range (whiskers). The mean MOC/SOC is 69.2% across sites, and the 1st and 3rd quartiles are 55% and 86.3%, respectively. Higher MOC generally results in higher MOC/SOC and, in > 75% of the observations, more than 50% of SOC is MOC.

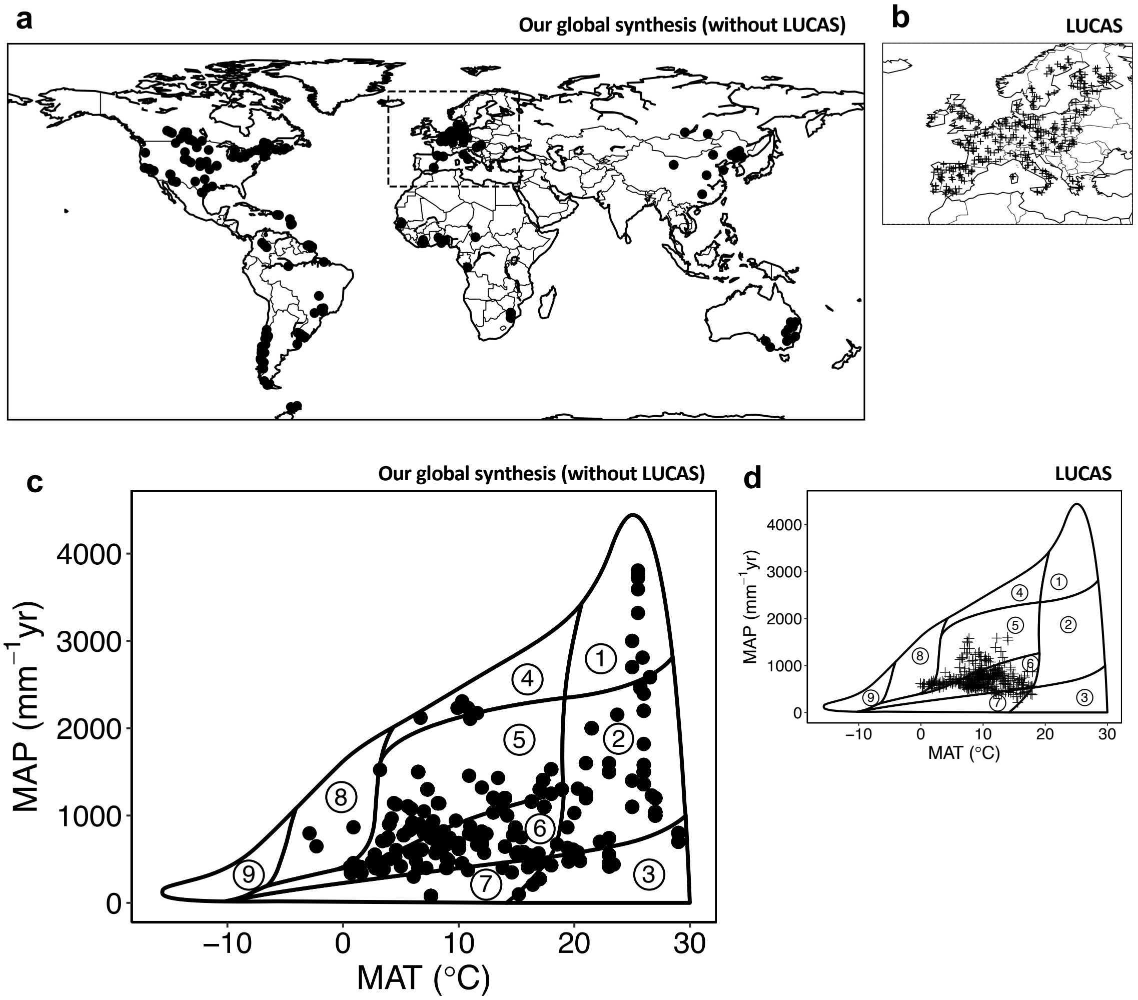

Supplementary Figure 2 | Location and climate of mineral-associated organic carbon measurements in our observational synthesis. a,b, Global distribution of 1,144 soil profiles spanning biomes and climate zones. **c,d,** Climate distribution of the soil profiles with wide ranges in mean annual temperature (MAT; °C) and mean annual precipitation (MAP; mm yr^{-1}). Soil profile measurements were synthesized from literature studies **(a,c)** (Supplementary Table 3) and the European LUCAS database[5,8] **(b,d)**. Model results and global estimates were robust to the addition of the LUCAS database (see Methods; Supplementary Fig. 11-15). Black polygons depict Whittaker's biomes[47] according to MAT and MAP values, following: (1) tropical rainforest; (2) tropical seasonal rainforest/savanna; (3) subtropical desert; (4) temperate rainforest; (5) temperate seasonal forest; (6) woodland/shrubland; (7) temperate grassland/desert; (8) boreal forest; and (9) tundra.

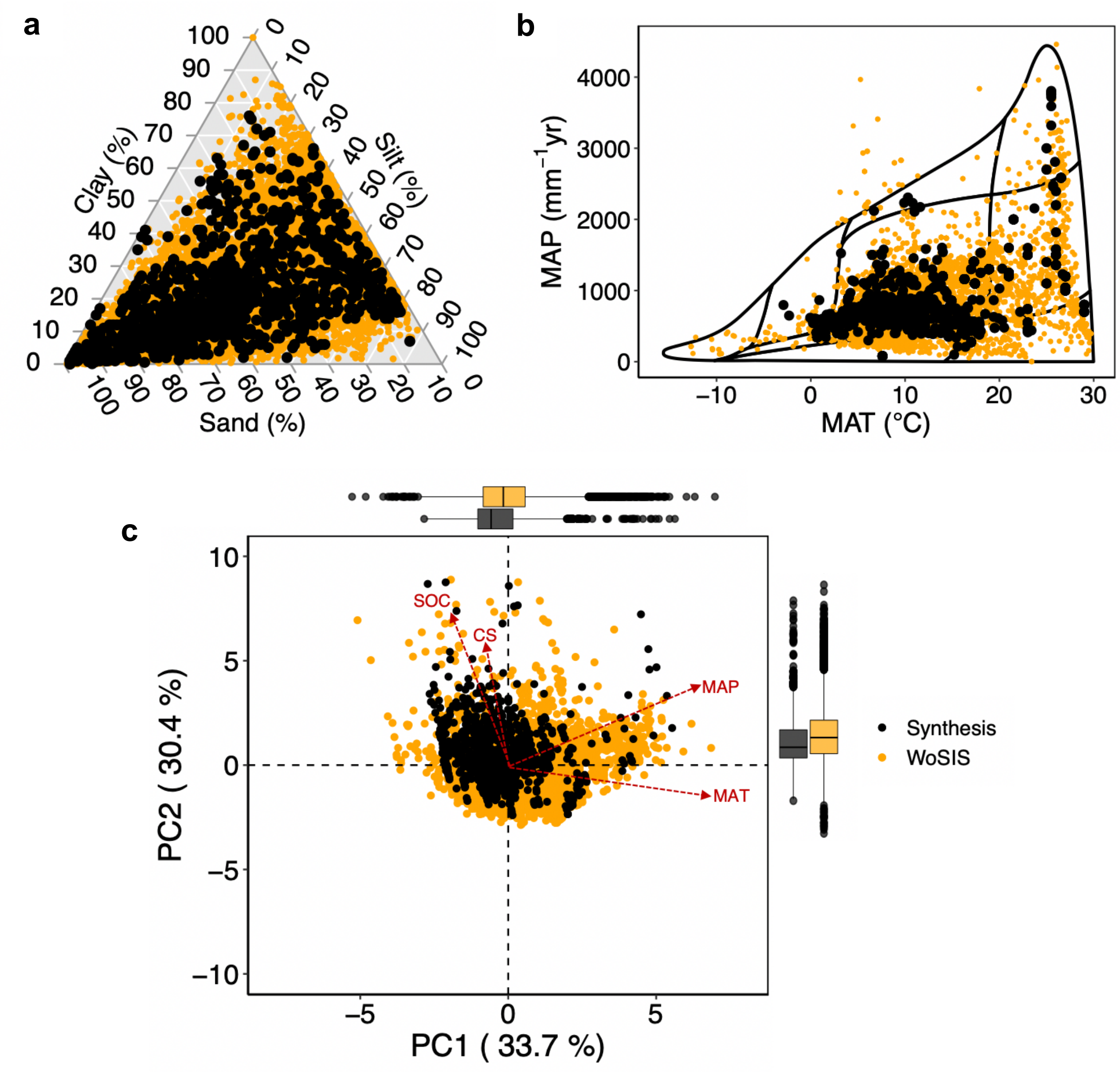

Supplementary Figure 3 | Representativeness of our global observational synthesis. Edaphic, climate, and multi-dimensional covariate space of the 1,144 study profiles (labeled 'Synthesis') compared to 10,000 randomly-sampled WoSIS (World Soil Information Service[48]) profiles. **a,** Soil texture triangle depicting the distribution of clay, silt, and sand among the global synthesis and WoSIS profiles. **b,** Mean annual temperature (MAT) and mean annual precipitation (MAP) distribution of the global profiles. Black polygons depict Whittaker's biomes[47]. **c,** Principal component analysis (PCA) depicting the multi-dimensional covariate space, where the principal components (PC1 and PC2) explain 64.1% of the variance and are composed of soil organic carbon (SOC), clay and silt (CS) content, MAT and MAP. Box plots show the corresponding marginal distributions and indicate the medians (horizontal lines), 1st and 3rd quartiles (boxes), and 1.5 × interquartile range (whiskers).

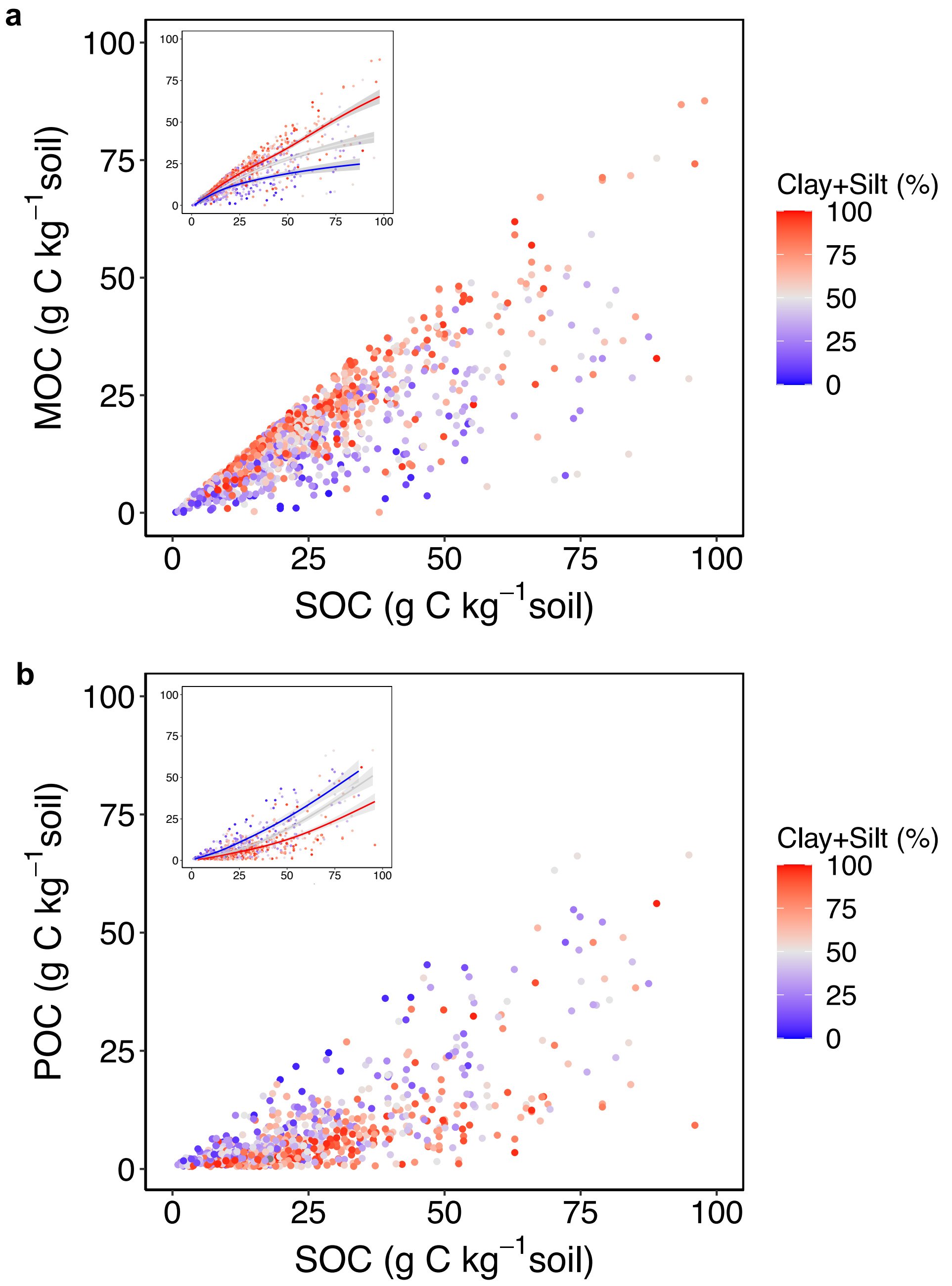

Supplementary Figure 4 | Mineral-associated and particulate organic carbon in our observational synthesis. a, Mineral-associated organic carbon (MOC) and **b,** particulate organic carbon (POC) as functions of total soil organic carbon (SOC) and clay + silt (CS) content. MOC and POC were measured and reported independently. MOC begins to saturate at high SOC, but the saturation level varies with mineral content and composition (Fig. 1). Insets depict best fit generalized additive models for three subsets of CS: namely, CS < 30% in blue, 30% < CS < 60% in grey, and CS > 60% in red.

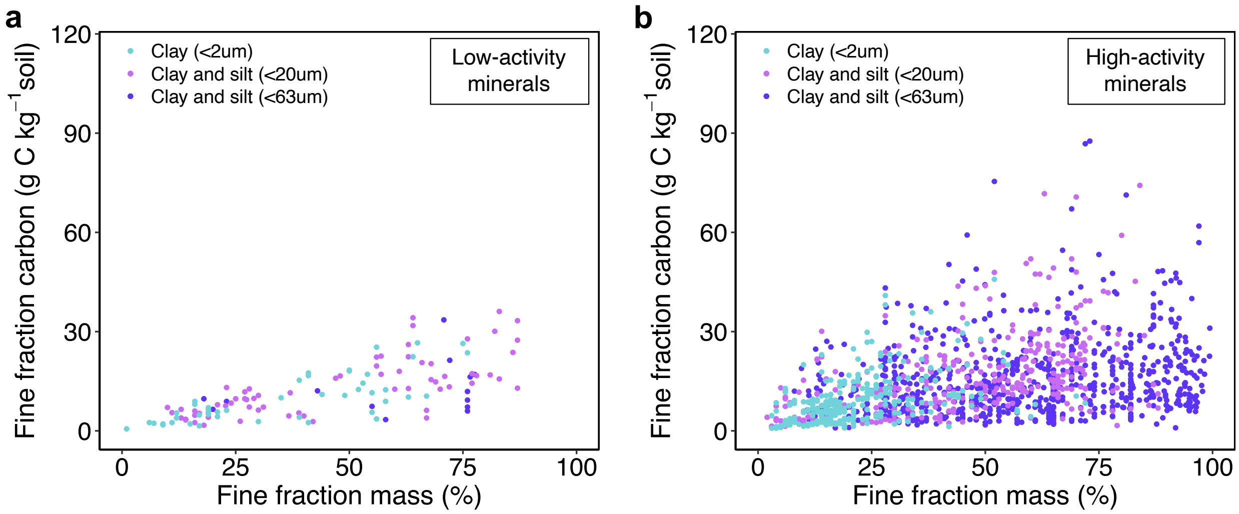

Supplementary Figure 5 | Mineral-associated carbon in clay and silt fractions. Mineral-associated (fine-fraction) carbon in **a,** low-activity and **b,** high-activity mineral soils across our observational synthesis, as a function of mineral (fine fraction) content by mass (i.e., clay or clay + silt percent). The maximum mineral-associated carbon capacity is robust to the choice of particle size thresholds.

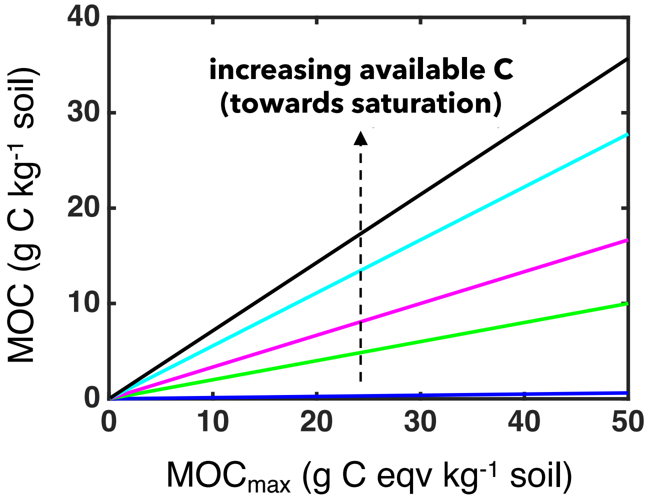

$$MOC = MOC_{max} \frac{K_{eq} \cdot C}{1 + K_{eq} \cdot C}$$

Supplementary Figure 6 | Illustration of predicted mineral-associated organic carbon across different concentrations of non-mineral-associated carbon for a given mineralogical capacity. Model-derived predictions (using the Langmuir sorption equation shown) of achieved mineral-associated carbon (MOC) as a function of the total mineralogical capacity (MOC_{max}) across increasing levels of non-mineral-associated 'available' carbon (denoted *C*). As the available C increases, MOC tends towards saturation, which would fall on the 1:1 line when MOC is equal to MOC_{max}. Here the equilibrium constant K_{eq} (i.e., the ratio of adsorption to desorption rate constants, k_{ads}/k_{des}) is set to 25 and C varies from 0.0005 to 0.1 kg C m^{-3}, corresponding to typical dissolved organic carbon concentrations.

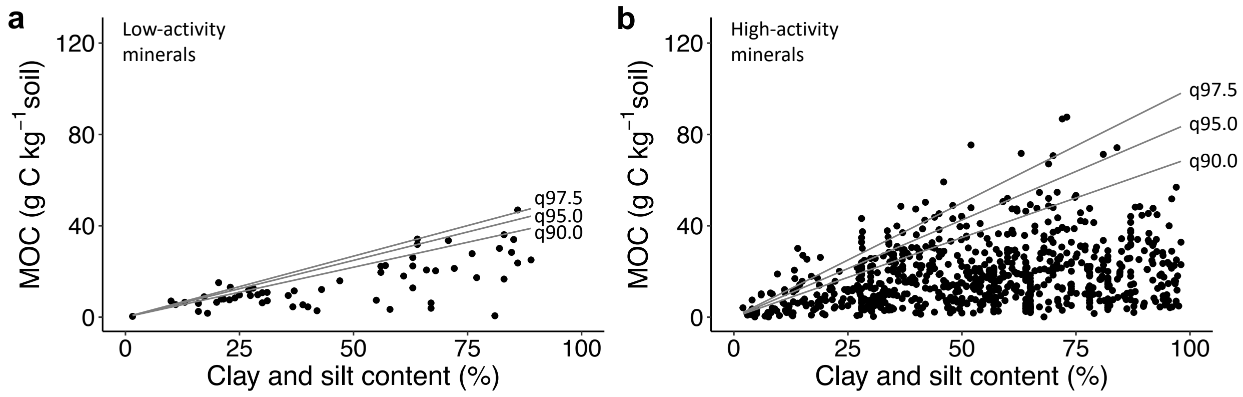

Supplementary Figure 7 | Sensitivity analysis of maximum mineral-associated carbon quantiles in high- and low-activity mineral soils. Mineral-associated organic carbon (MOC; g C kg^{-1} soil) as a function of clay and silt content (CS; %) in **a,** low- and **b,** high-activity mineral soils (HM and LM, respectively). The slopes denote 90th, 95th, and 97.5th quantile regressions for each mineral type (abbreviated q90.0, q95.0, q97.5, respectively) and illustrate the sensitivity of MOC_{max} to the selected quantile. The 95th quantile was selected as a conservative estimate of the MOC_{max} boundary line given exiting experimental uncertainty of individual observations and percent fractionation recovery across studies (see Methods; Fig. 1). Furthermore, the 90% confidence intervals of the 95th quantiles (shaded in Fig. 1) cover the spread seen across the 90th and 97.5th quantiles (q90 and q97.5 slopes = 0.43 and 0.51 for LM, 0.76 and 0.98 for HM, respectively), and provide a conservative estimate of MOC_{max} and its uncertainty for each mineral type.

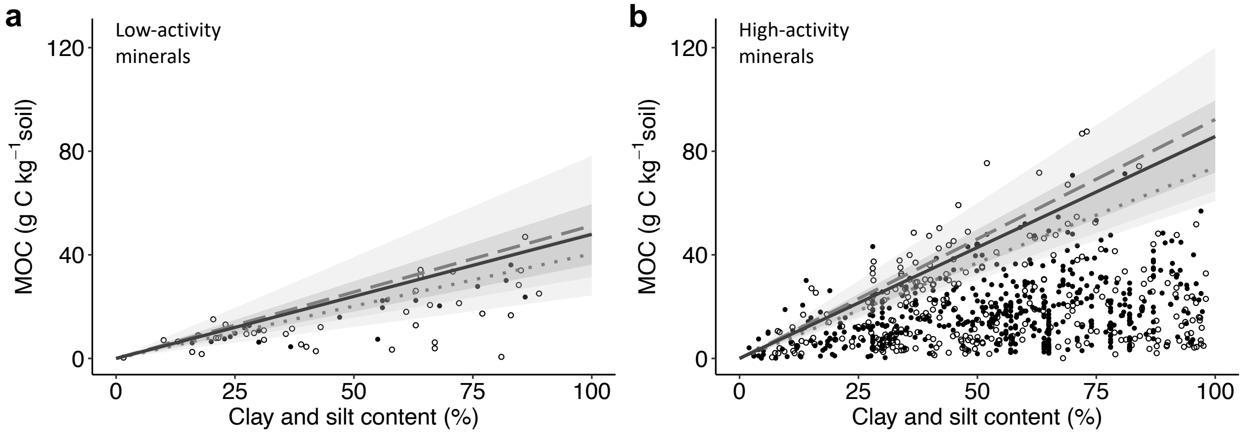

Supplementary Figure 8 | Maximum mineral-associated carbon in high- and low-activity minerals by vegetation type. Mineral-associated organic carbon (MOC; g C kg^{-1} soil) as a function of clay and silt content (CS; %) in **a,** low- and **b,** high-activity mineral soils (HM and LM, respectively). The lines depict MOC_{max} boundaries, calculated as 95th quantiles with shaded 90% confidence intervals (see Methods). For each mineral type, quantiles are given for grasslands (closed circles; dotted lines) and forests (open circles; dashed lines), which are compared to the combined estimates based solely on mineralogy and irrespective of vegetation type (solid lines; as in Fig. 1). The slopes for grasslands and forests were not significantly different within each mineral type (0.44 ± 0.04 and 0.51 ± 0.10 for LM, respectively; 0.74 ± 0.07 and 0.92 ± 0.15 for HM), and were consistent with the mineralogy-driven value irrespective of vegetation type (solid lines and shading; slopes = 0.48 ± 0.06 for LM and 0.86 ± 0.09 for HM). While the slopes for grasslands trended lower than those for forests, this was not statistically significant ($p > 0.1$) and future work is needed to explore the role of litter quality on the chemical composition of downstream microbial residues and the mineralogical carbon capacity.

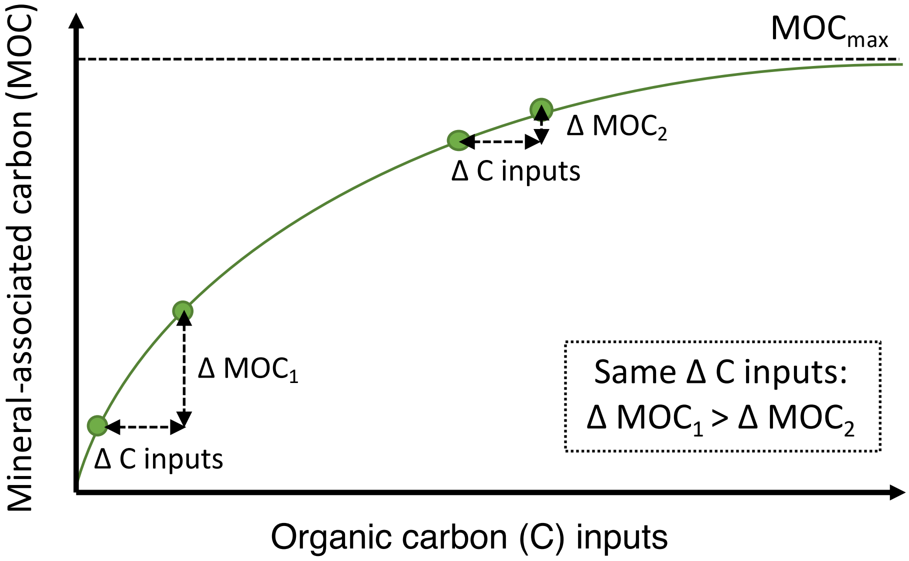

Supplementary Figure 9 | Greater return on investment in soils at lower carbon saturation. For the same increase (or decrease) in C inputs (or suitable proxy for C available for sorption; e.g., given microbial feedbacks and limitations, as well as the quality and location of inputs[6,23,33]), more mineral-associated carbon can be gained (or lost) further from carbon saturation[4,39,49] (i.e., further from MOC_{max}). This suggests that the largest potential for sequestering soil carbon is in degraded lands, but also that these lands may be most vulnerable to further change.

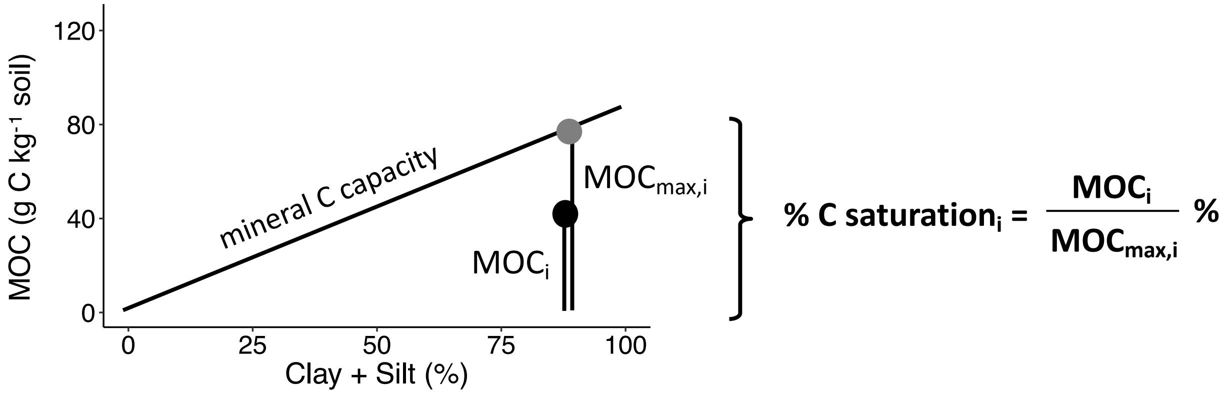

Supplementary Figure 10 | Schematic illustrating the calculation of percent carbon saturation. The percent carbon saturation is defined as the proximity of the observed mineral-associated organic carbon (MOC) at each site i to the maximum mineralogical capacity (MOC_{max}) at that site.

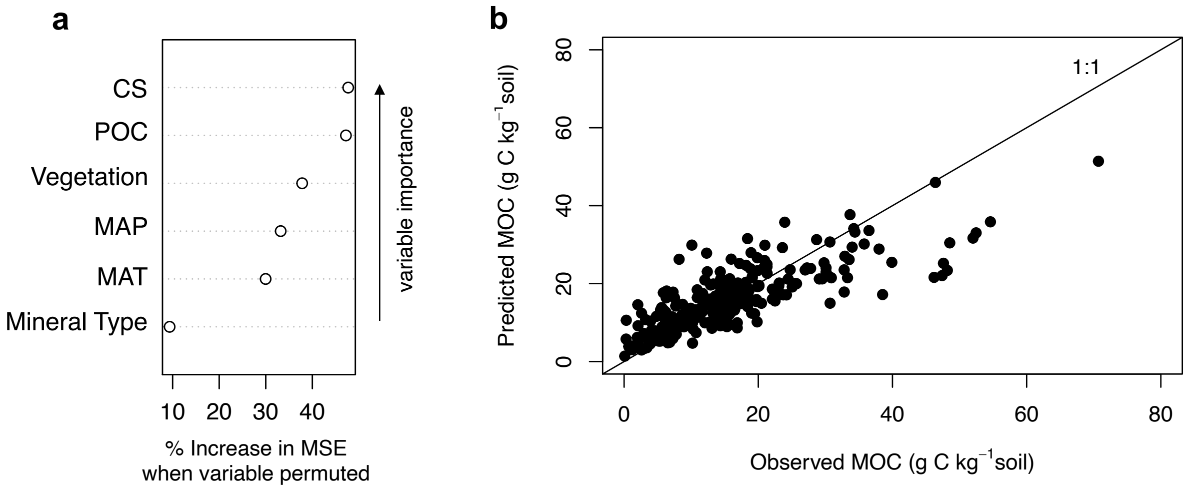

Supplementary Figure 11 | Variable importance and performance of the machine learning random forest model trained on the observational synthesis data. a, Variable importance plot with key covariates: percent clay and silt (CS), particulate organic carbon (POC), mean annual temperature (MAT), mean annual precipitation (MAP) as continuous variables, and vegetation and mineral type as categorical variables. The data were randomly sampled to create independent training and test datasets (75% and 25% split, respectively). Variable importance is ranked by the percent increase in mean square error (MSE) when each individual variable is permuted. **b,** Model-predicted versus observed MOC for the test dataset, where accurate predictions fall on the 1:1 line. An ensemble of random forest models (n = 300 models; 400 decision trees each) with bootstrapped sampling was used for a robust assessment of model accuracy and predictive power – in comparing predicted and observed MOC for the independent test datasets across the ensemble, mean absolute error (MAE) = 4.8 ± 0.3 g C kg^{-1} soil and R^2 = 0.60 ± 0.06 (mean ± s.d.).

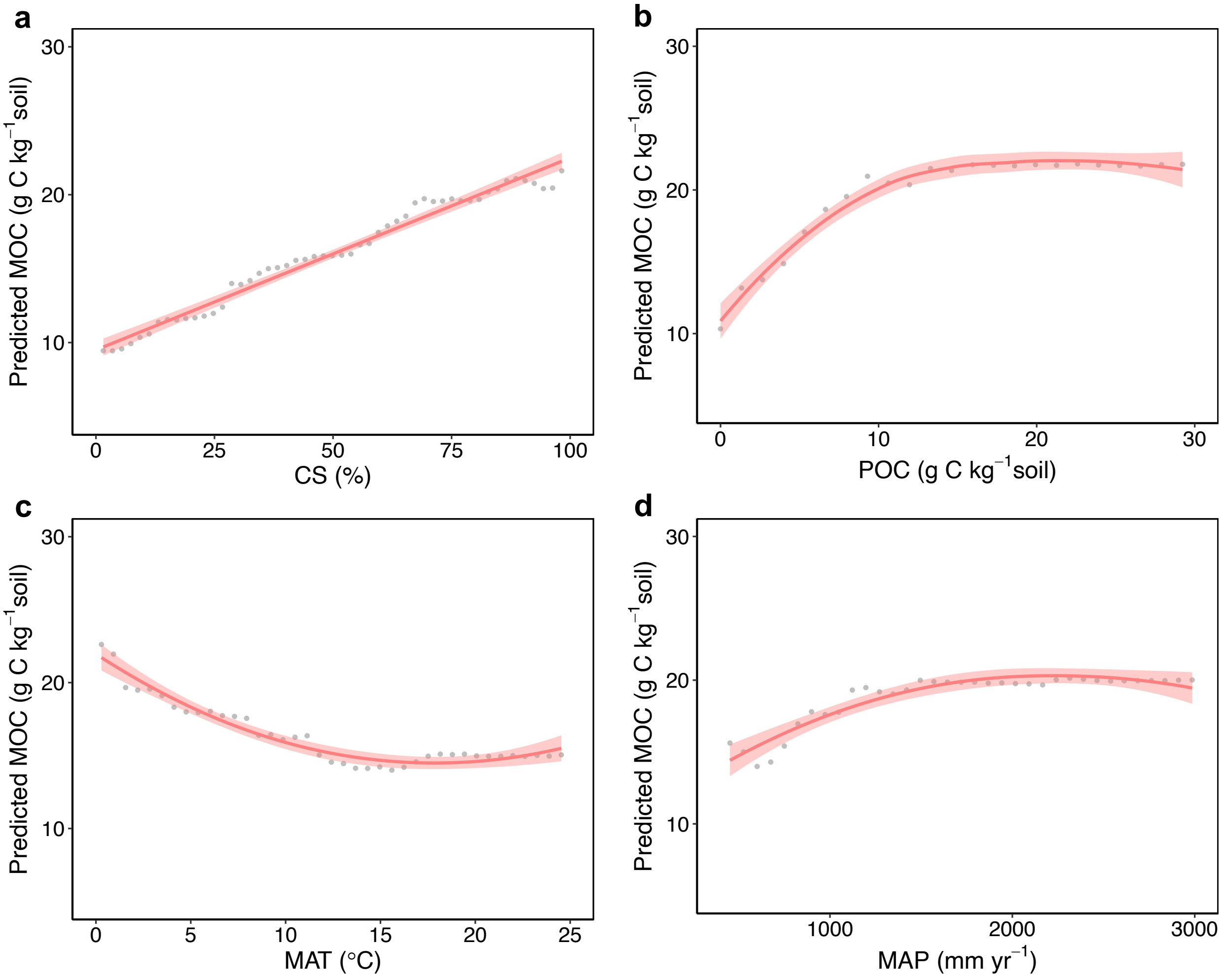

Supplementary Figure 12 | Data-driven predictive relationships of mineral-associated organic carbon as a function of individual controlling factors. A random forest model was used to disentangle the significance and emergent relationships of individual covariates ($R^2 = 0.60 \pm 0.06$; mean ± s.d.). Partial dependence relationships are shown, describing the marginal effect of each individual variable on the predicted MOC response. Predicted mineral-associated carbon (MOC) as a function of **a,** clay and silt content (CS), **b,** particulate organic C (POC), **c,** mean annual temperature (MAT), and **d,** mean annual precipitation (MAP). For each plot, the points show random-forest model predictions; best fit lines show 99% confidence intervals on the trend.

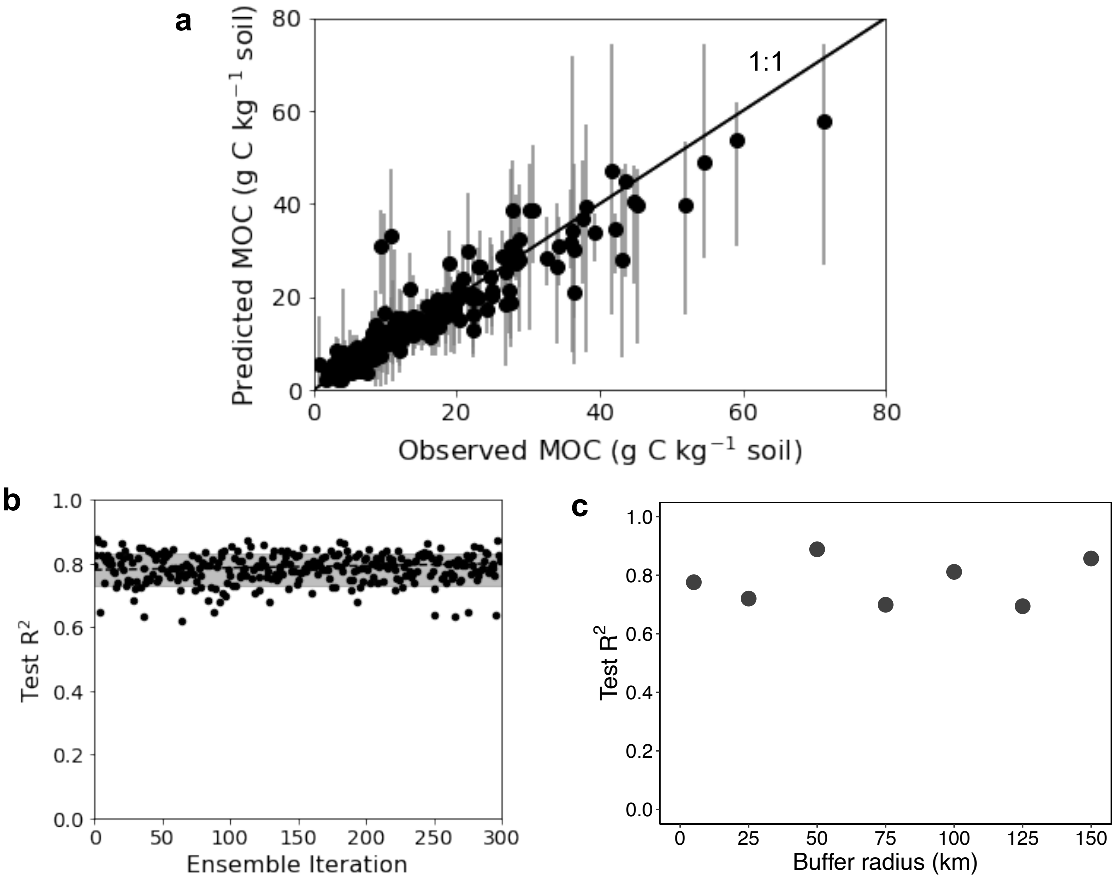

Supplementary Figure 13 | Performance of the random forest model trained on the observational synthesis data for global predictions. a, Model-predicted versus observed MOC for the test dataset, where accurate predictions fall on the 1:1 line. Error bars show 5th and 95th quantiles, denoting 90% prediction intervals. Covariates used in the random forest (RF) model included clay and silt, mean annual temperature and precipitation, soil organic carbon, and vegetation and mineral type. **b,** An ensemble of 300 RF models with bootstrapped sampling[50] (75%-25% train-test split each) was used to rigorously assess predictive performance. Dashed black line and grey shading depict the ensemble mean and standard deviation ($R^2 = 0.79 \pm 0.05$; mean ± s.d.). **c,** Spatially-buffered leave-one-out cross-validation[51,52] was also performed to ensure that spatial auto-correlation did not significantly compromise the predictive performance of the RF model. For each leave-one-out test data point (n = 200 randomly-sampled points for each buffer radius, for a total of 1,400 RF models), all data within a given buffer radius (0 to 150km) were excluded from the training set; i.e., only data points outside the buffer zone were used to train each RF model. R^2 values did not vary with buffer radius (regression slope not statistically different from zero; $p = 0.81$) and were in agreement with the ensemble cross-validation approach.

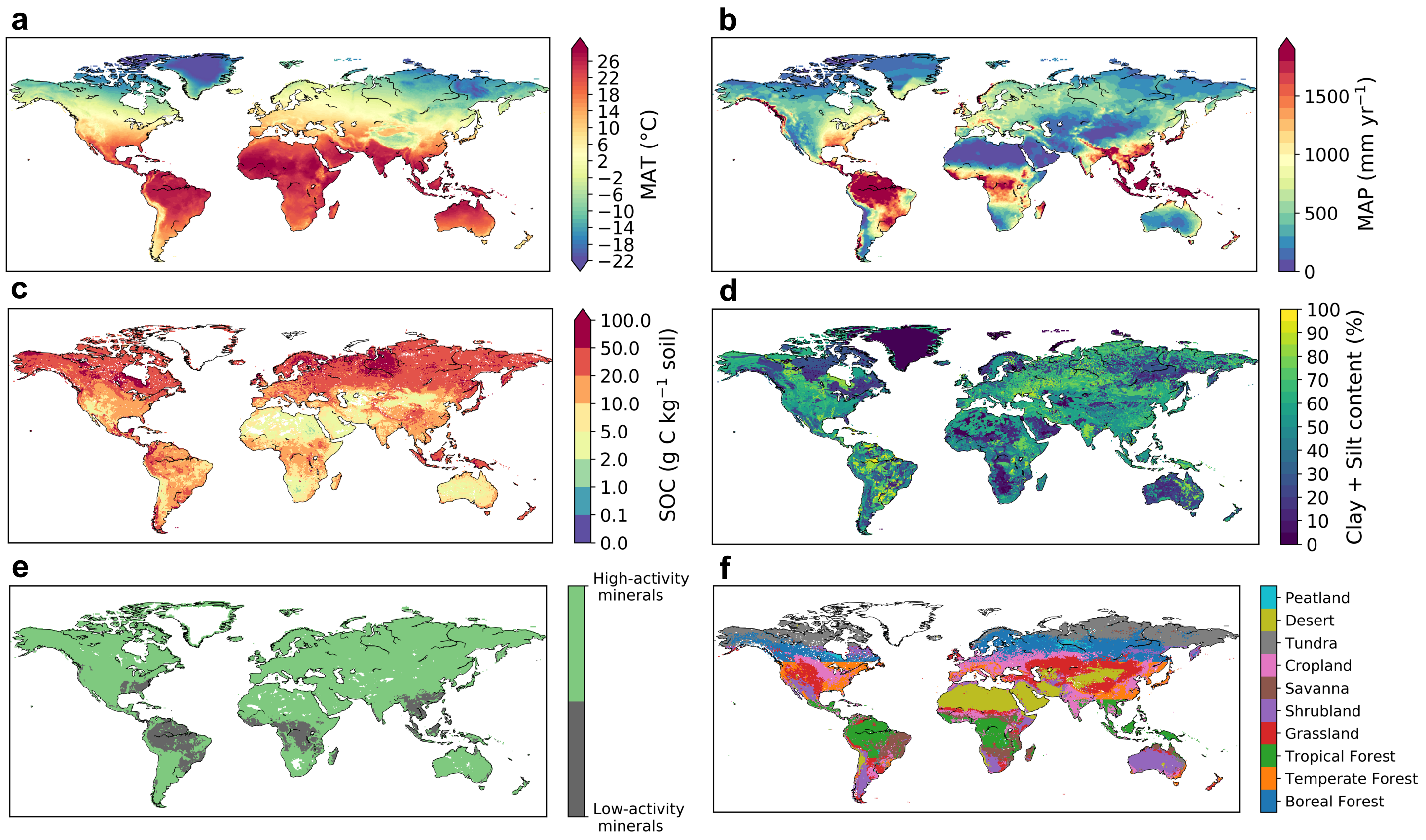

Supplementary Figure 14 | Covariates used in the machine-learning random-forest model trained on the observational synthesis data and used to make global predictions. a, Mean annual temperature (MAT) and **b,** mean annual precipitation (MAP). **c,** Soil organic carbon (SOC) derived as the mean of two global data products (HWSD and SoilGrids; see Methods). Global SOC stocks are summarized in Supplementary Tables 1 and 2. **d,** Clay and silt (CS) content and **e,** primary mineral type. SOC, CS, and mineral type are shown here for topsoil (< 30 cm). All maps are at 0.5° resolution. **f,** Land cover type modified from the MODIS MCD12C1 product[53]. The 16 MODIS IGBP land cover types were combined into 10 types to match the biome types in the observational synthesis (following ref. [54]). Namely, all forests and woody savannas were categorized as boreal (> 50°N), temperate (> 23° and < 50°N and S), or tropical (< 23°N and S) forest; open and closed shrublands were combined as shrubland (< 60°N and S) or tundra (> 60°N); and the rest were unchanged. The observational synthesis included MOC measurements from boreal forest (n = 41 soil profiles), temperate forest (n = 141), tropical forest (n = 29), shrubland (n = 31), grassland (n = 305), and savanna (n = 38), as well as croplands (see Methods; Supplementary Table 3). Peatlands, deserts, and tundra were not included in the analysis due to insufficient sample sizes and the focus on non-permafrost, non-desert mineral soils.

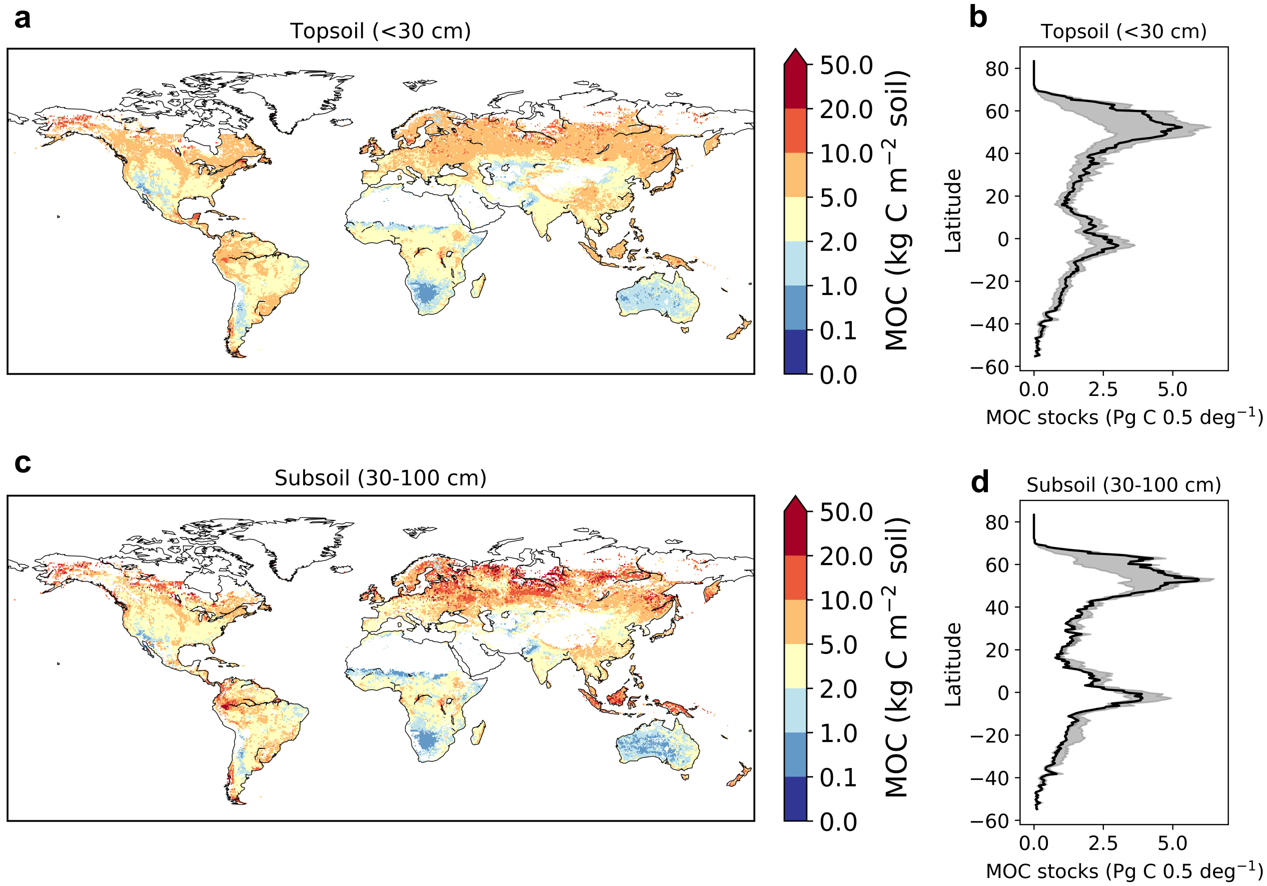

Supplementary Figure 15 | Global mineral-associated carbon predictions and uncertainty. **a,c,** Current global mineral-associated carbon (MOC) at 0.5° resolution in the topsoil (< 30 cm) and subsoil (30-100 cm), respectively, excluding tundra, peatlands, and deserts. Values were predicted using a data-driven random forest model (R^2 = 0.79 ± 0.05; mean ± s.d.) with global climate, edaphic properties, and land cover. **b,d,** Latitudinal MOC stocks (Pg C 0.5 deg^{-1} latitude) in the topsoil and subsoil, respectively. Black lines represent the latitudinal stocks summed by 0.5° latitudinal bands. Grey shading represents the 5th and 95th quantiles, denoting the 90% prediction intervals from the random forest model. Global stocks and uncertainties are summarized in Supplementary Tables 1 and 2.

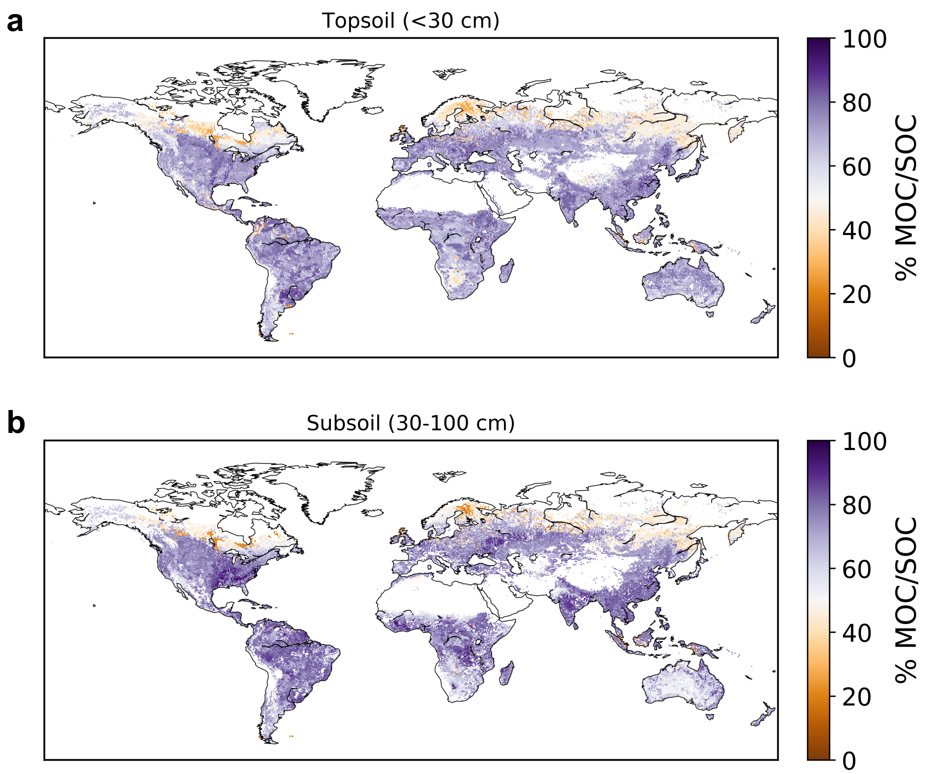

Supplementary Figure 16 | Proportion soil organic carbon that is mineral-associated globally. Percentage of mineral-associated organic carbon (MOC) in soil organic carbon (SOC) in the **a,** topsoil (< 30 cm) and **b,** subsoil (30-100 cm), excluding tundra, peatland, and deserts. The remaining carbon that is not mineral-associated constitutes particulate organic carbon (POC). MOC/SOC ratios averaged 0.66 ± 0.13 (mean ± s.d.) and 0.70 ± 0.17 in topsoils and subsoils, respectively, and 0.69 ± 0.15 across depths globally. MOC/SOC ratios in tundra and peatlands (not shown) averaged 0.58 ± 0.15 and 0.34 ± 0.26 across depths, respectively, but additional measurements are needed to verify estimates in these ecosystems. Proportionally more MOC is stored in subsoils, and proportionally less MOC (more POC) is stored at high-latitudes.

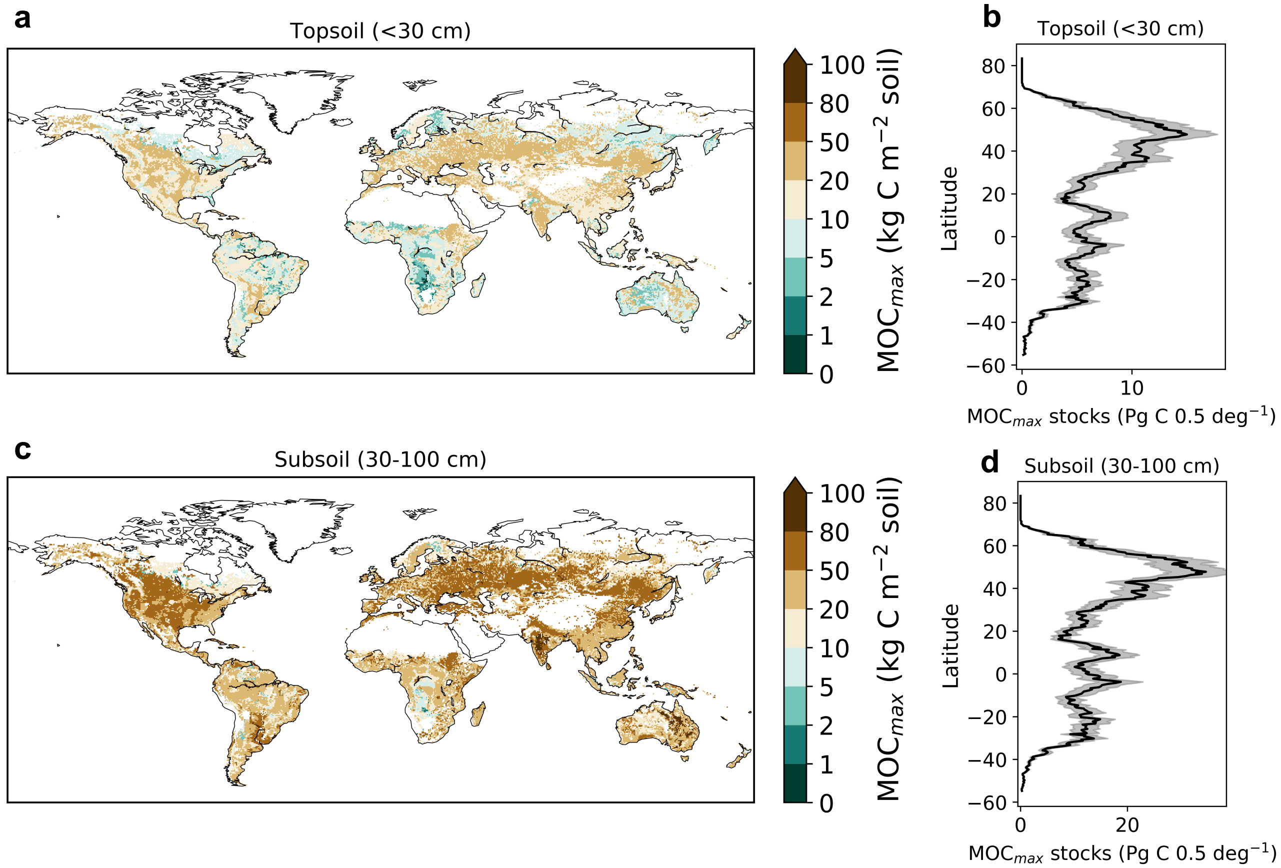

Supplementary Figure 17 | Global soil mineralogical carbon capacity and uncertainty intervals. a,c, Mineralogical carbon capacity (MOC_{max}; kg C m^{-2} soil) at 0.5° resolution in the topsoil (< 30 cm) and subsoil (30-100 cm), respectively, excluding tundra, peatlands, and deserts. MOC_{max} is calculated from the amount and type of soil minerals globally (Fig. 1), and constitutes an upper mineralogical potential. **b,d,** Latitudinal MOC_{max} stocks (Pg C 0.5 deg^{-1} latitude) in the topsoil and subsoil, respectively. Black lines represent the latitudinal stocks summed by 0.5° latitudinal bands. Grey shading depicts 90% confidence intervals of the mineralogical capacity slopes of high- and low- activity minerals. Global stocks and uncertainties are summarized in Supplementary Table 1.

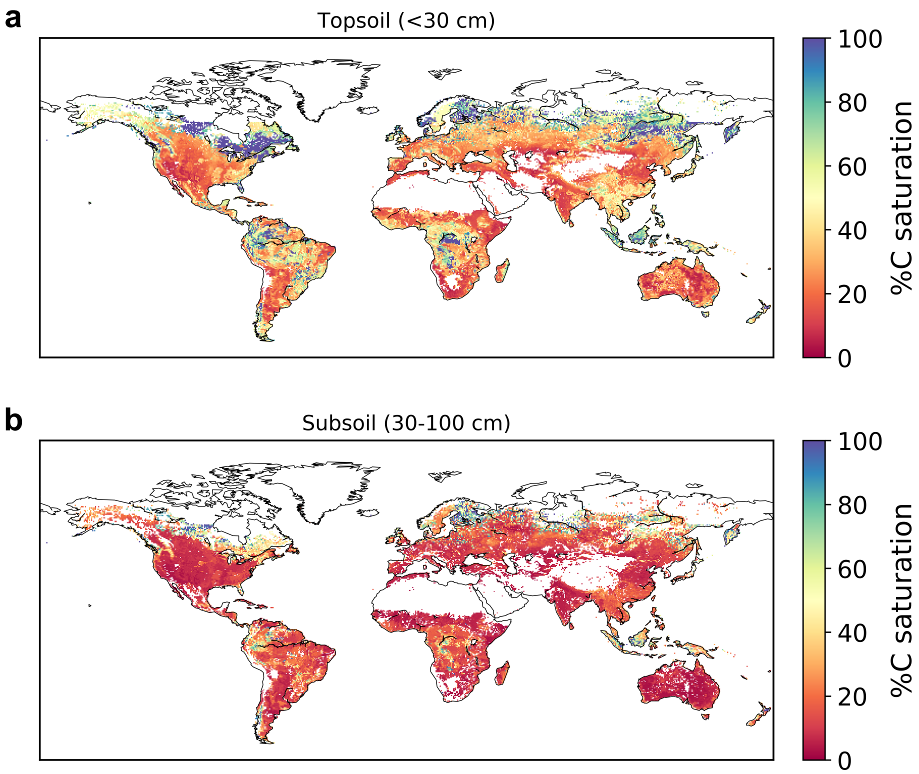

Supplementary Figure 18 | Percent mineralogical carbon saturation globally. Percent mineralogical carbon saturation (%C saturation) calculated as the current mineral-associated organic carbon (MOC) divided by the mineralogical carbon capacity (MOC_{max}) in the **a,** topsoil (< 30 cm) and **b,** subsoil (30-100 cm), excluding tundra, peatlands, and deserts. %C saturation averaged 27% (16, 38) across depths, and 42% (5 to 95% range: 24, 61) and 21% (14, 30) in topsoils and subsoils, respectively. %C saturation in tundra and peatlands (not shown) averaged 45% (18, 62) and 63% (23, 97) across depths, respectively; topsoils and subsoils averaged 62% (23, 91) and 38% (17, 51) in tundra and 73% (25, 109) and 60% (23, 95) in peatlands, but additional measurements are needed to verify estimates in these ecosystems. Global topsoil and subsoil averages for all datasets are summarized in Supplementary Table 2.

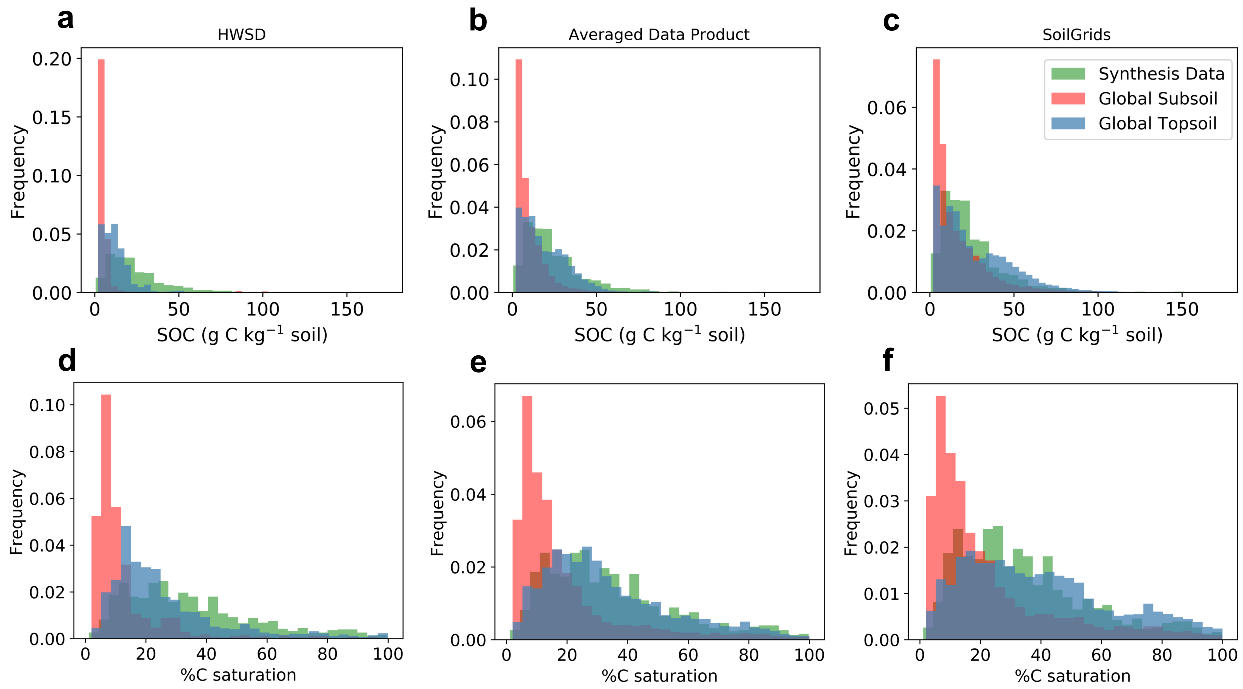

Supplementary Figure 19 | Distribution of soil carbon and percent mineralogical carbon saturation across data products. a-c, Soil organic carbon (SOC) from HWSD (**a**), SoilGrids (**c**), and the averaged data product derived from the two (**b**; see Methods), in topsoils and subsoils, compared to the distribution of SOC from the observational synthesis data (all depths). **d-f,** Percent mineralogical carbon saturation (%C saturation) from the global predictions using HWSD (**d**), SoilGrids (**f**), and the averaged data product derived from the two (**e**), compared to the synthesis data (all depths). Global topsoil and subsoil carbon stocks and %C saturation are summarized for all three data products in Supplementary Table 2.

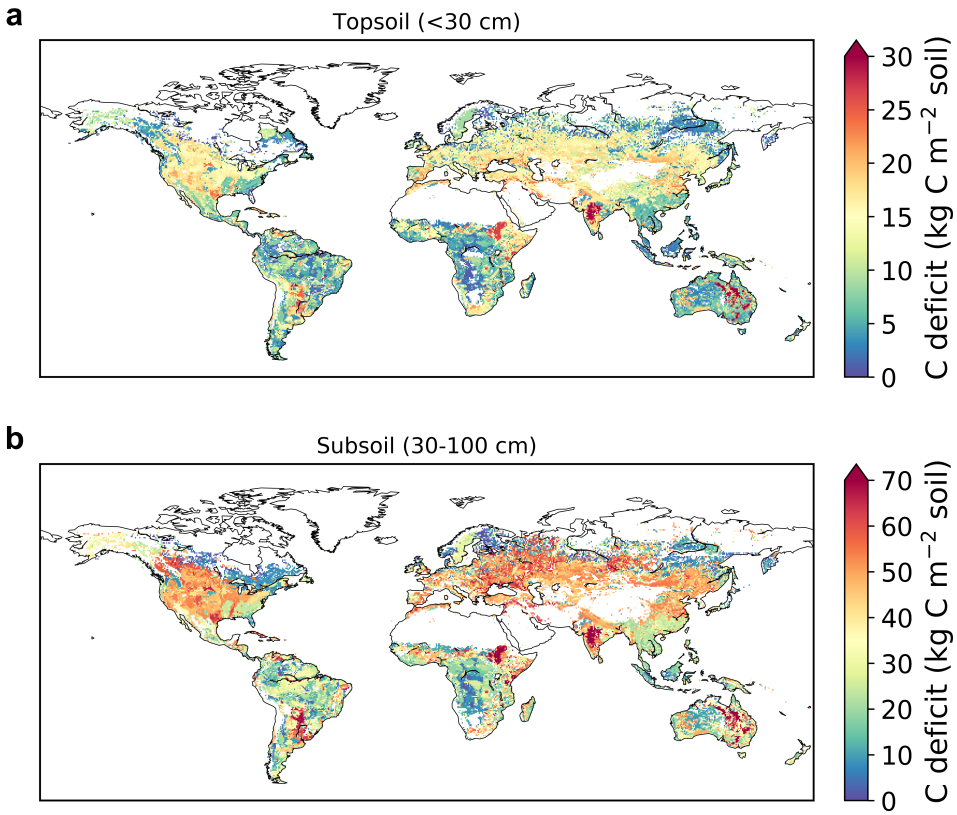

Supplementary Figure 20 | Global soil mineralogical carbon deficit across the soil profile. **a,b,** Soil C deficit (kg C m^{-2} soil) at 0.5° resolution in the topsoil (< 30 cm) and subsoil (30-100 cm), respectively, excluding tundra, peatlands, and deserts. C deficit is calculated as the difference between the mineralogical C capacity (MOC_{max}) and current mineral-associated organic C stocks (MOC). High C deficit signifies a low %C saturation and a potential for further C accrual. Global C deficits are summarized in Supplementary Table 1.

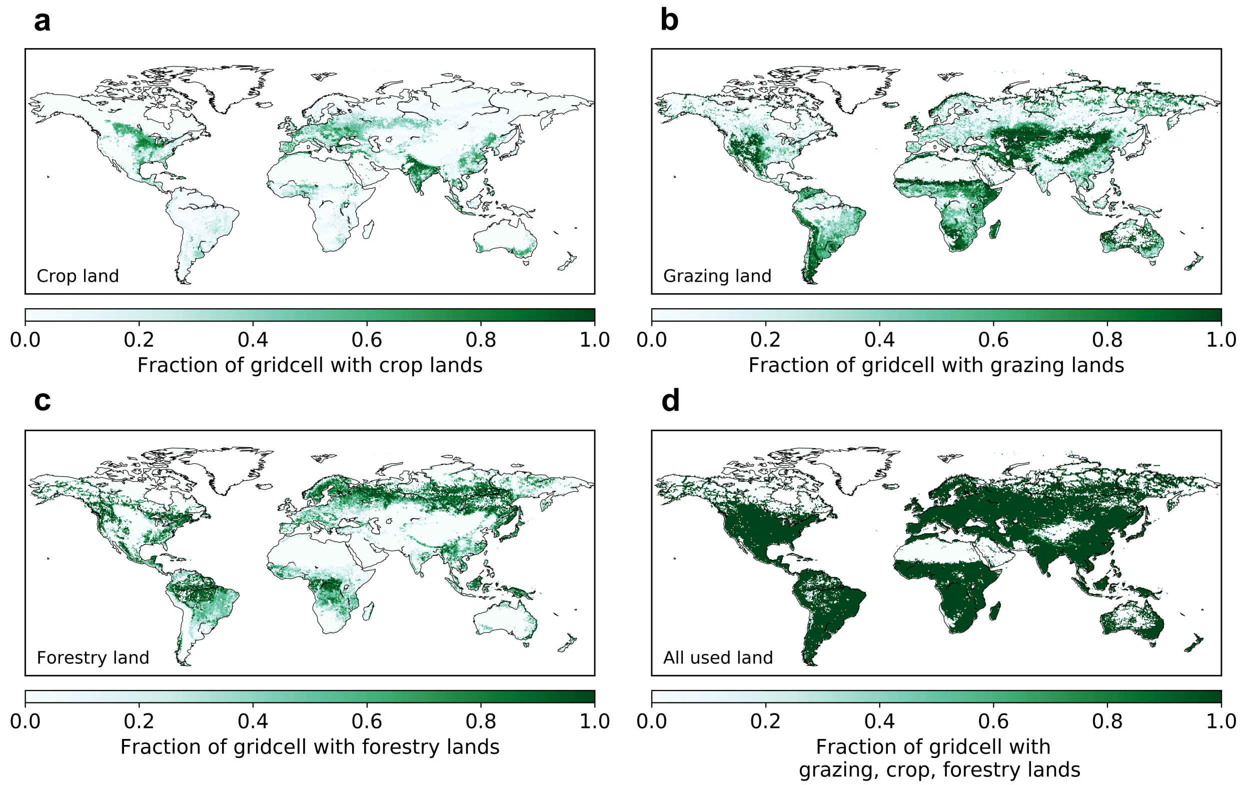

Supplementary Figure 21 | Global extent of lands that are dominated by management. Fraction of the land surface that is occupied by human land use through **a,** agricultural crops, **b,** grazing, and **c,** forestry. **d,** Combined used lands (crop, grazing, and forestry) showing regions dominated by management (i.e., fraction equal to 1 shown in dark green) and unused lands (shown in white)[55,56].

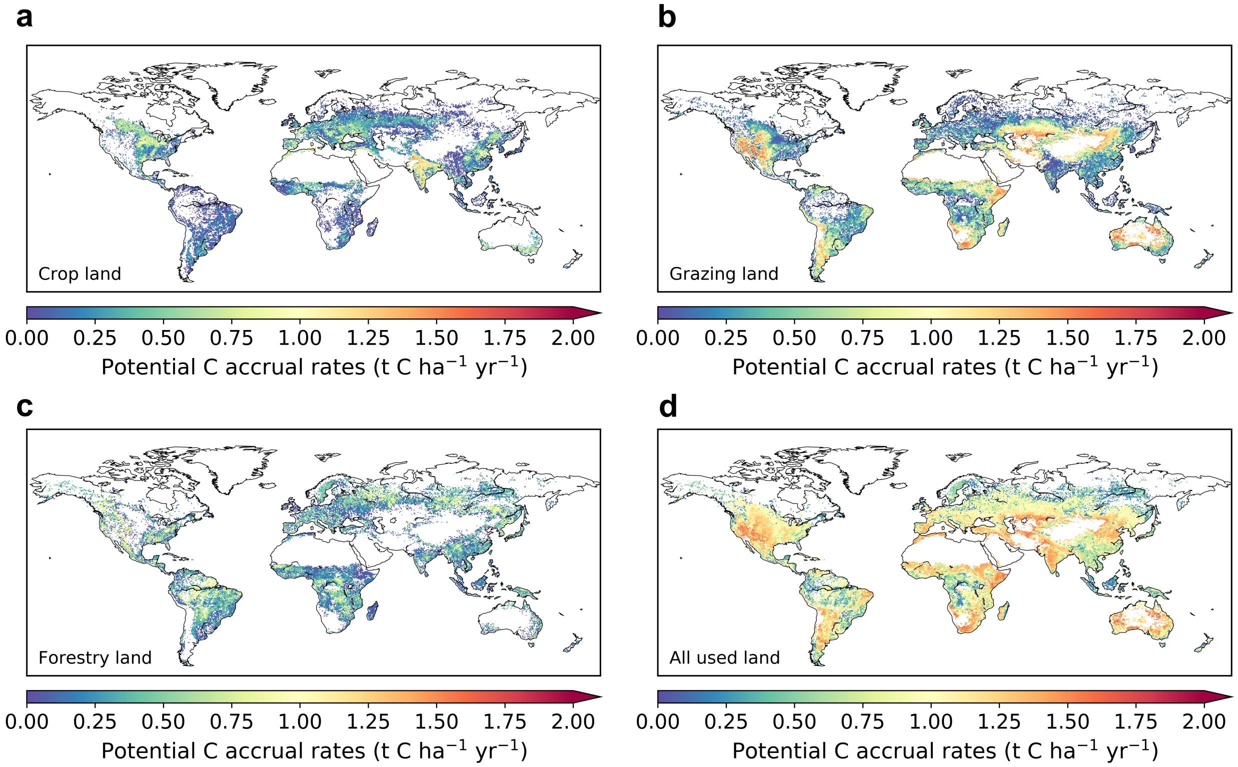

Supplementary Figure 22 | Potential carbon accrual rates globally. Potential C accrual rates (t C ha^{-1} yr^{-1}) in topsoils across **a,** crops, **b,** grazing, **c,** forestry, and **d,** combined used lands, based on the C accrual rates observed from our synthesis (Fig. 4d) and the current mineralogical C saturation globally (Fig 4c, using the averaged SOC data product; see Methods). Peatlands, tundra, and deserts are excluded. Globally, potential accrual rates total 1.4 Pg C yr^{-1} for crop land, 3.7 Pg C yr^{-1} for grazing land, and 2.1 Pg C yr^{-1} for forestry land (see Methods; 1.2 to 1.6 Pg C yr^{-1}, 3.4 to 4.3 Pg C yr^{-1}, and 1.7 to 2.9 Pg C yr^{-1} for crops, grazing, and forestry using SoilGrids and HWSD, respectively). These values are potential rates given current conditions, but would decrease over time as mineralogical C saturation was approached following Fig. 4d.

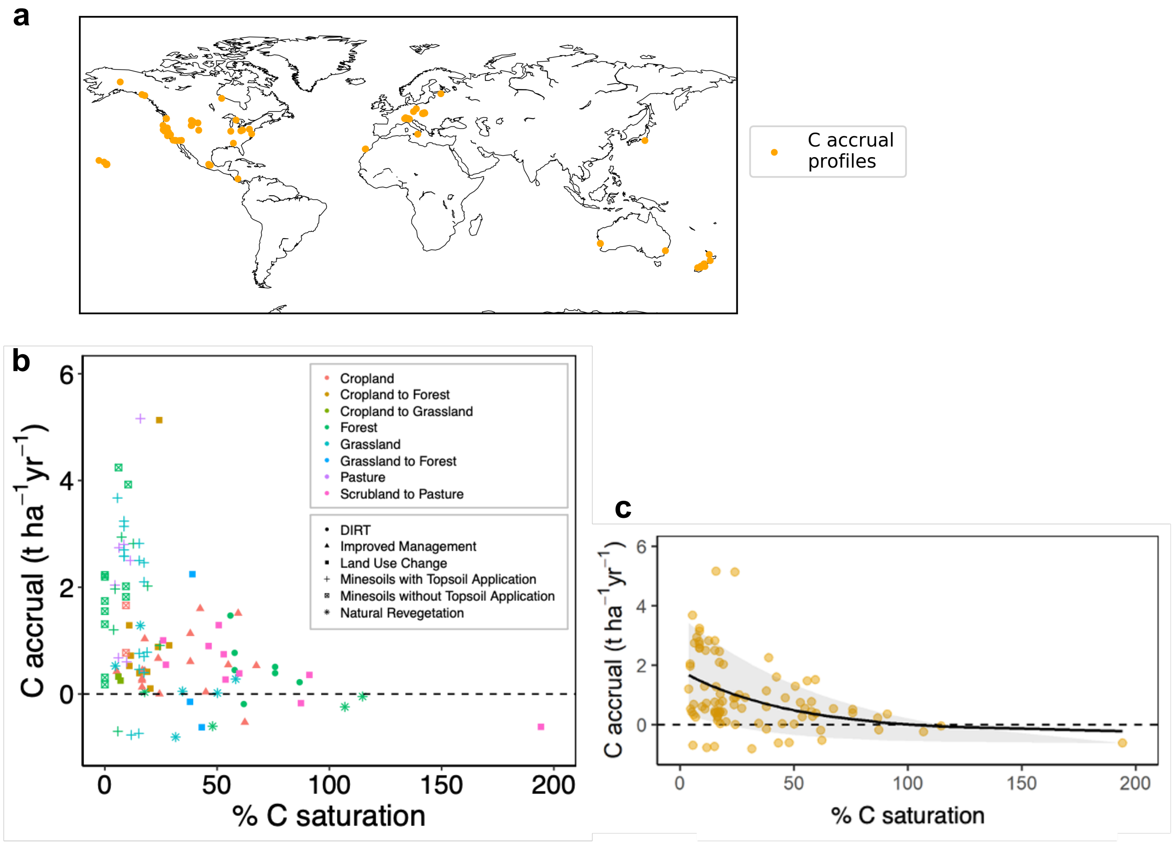

Supplementary Figure 23 | Carbon accrual rates by study type and soil order across globally-distributed sites varying in mineralogical carbon saturation. **a,** Location of C accrual studies. **b,** C accrual rates in topsoils (mean depth = 29 cm) following management interventions spanning a range of durations (n = 103; Supplementary Table 4). Studies included improved management (cover cropping, no till, crop-livestock rotation, deep ploughing, reduced grazing), land use change (cropland to grassland or forest), DIRT (Detrital Input and Removal Experiment with doubled litter or wood), and natural revegetation and minesoil reclamation (formerly degraded or newly exposed land surfaces as a result of mining, landsliding, alluviation, or marine terrace uplift, where soil C accrual can be expected following natural or human-accelerated revegetation and soil formation). While minesoils are well-described systems suitable for the study of soil carbon dynamics after land-use change[57,58], the observed negative trend in C accrual with %C saturation was robust to their exclusion. **c,** Carbon accrual in topsoil as a function of %C saturation across sites excluding mine soils without topsoil application (n = 90). Exclusion of all mine soils also shows a negative trend (linear fit, p = 0.056; not significantly different than the case with mine soils included). Nonlinear fit depicts asymptotic regression model with shaded areas representing 10th and 90th quantiles.

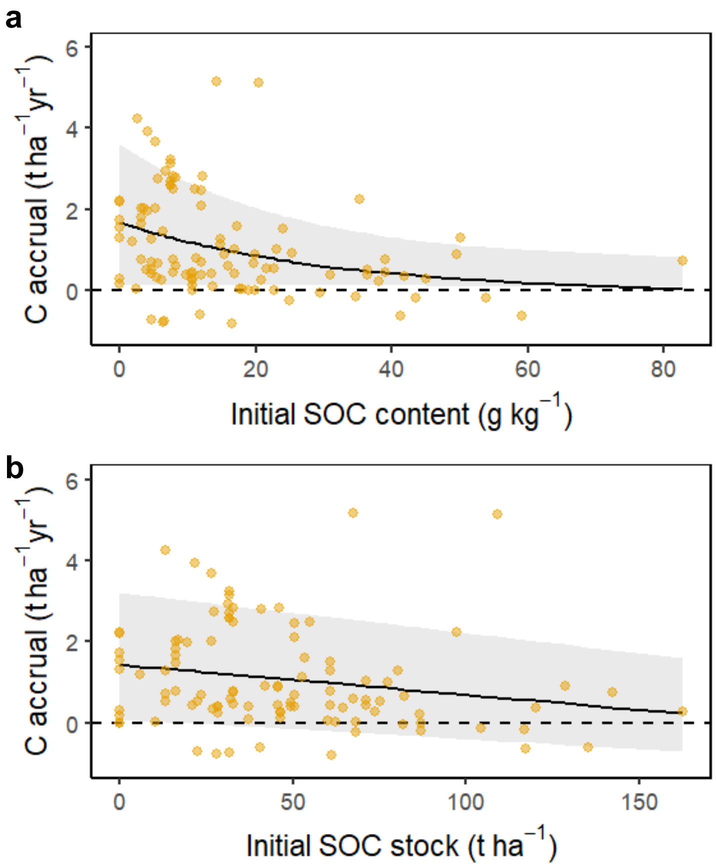

Supplementary Figure 24 | Carbon accrual rates as a function of initial soil organic carbon across globally-distributed sites. a,b, C accrual rates in topsoils (mean depth = 29 cm) following management interventions (n = 103; Supplementary Table 4) shown as a function of initial soil organic carbon (SOC) content and stock, respectively. Fits depict non-linear asymptotic regression (**a,** $R^2 = 0.10$) and linear regression (**b,** $R^2 = 0.041$) models with shaded areas representing 10th and 90th quantiles.

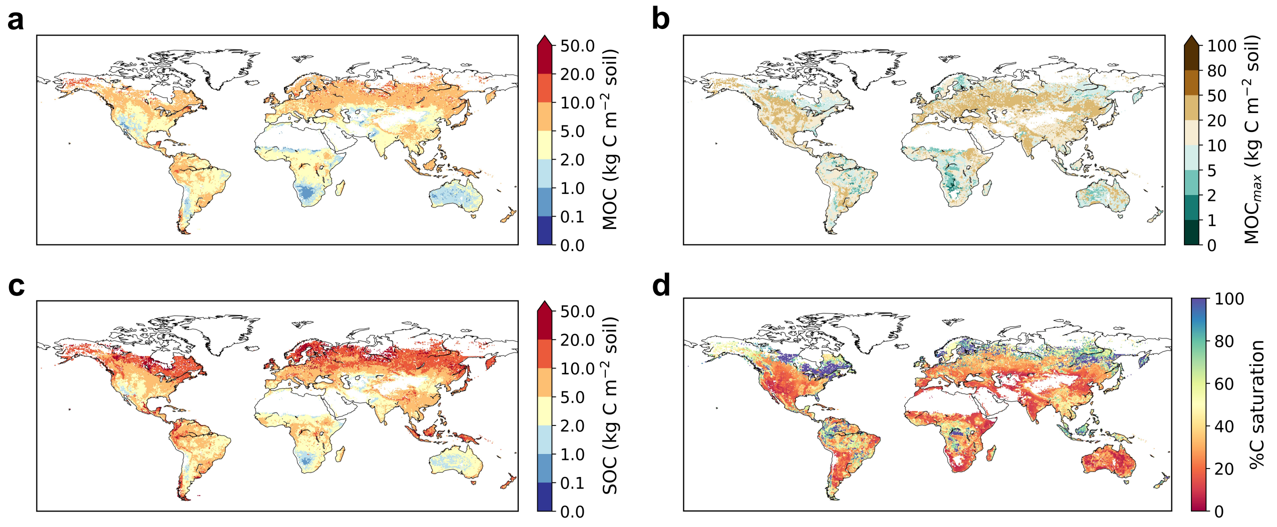

Supplementary Figure 25 | Summary of spatially-resolved soil carbon stocks and capacity. Global distribution of **a,** mineral-associated organic carbon (MOC; reproduced from Supplementary Fig. 15a), **b,** mineralogical carbon-storage capacity (MOC_{max}; reproduced from Supplementary Fig. 17a), **c,** total soil organic carbon (SOC; following Supplementary Fig. 14c), and **d,** mineralogical %C saturation (reproduced from Supplementary Fig. 18a). All quantities are shown for topsoils (< 30 cm) globally, excluding tundra, peatlands, and deserts. A schematic summarizing the derivation of each quantity is given in Supplementary Fig. 26, and further details are provided in the respective Methods sections.

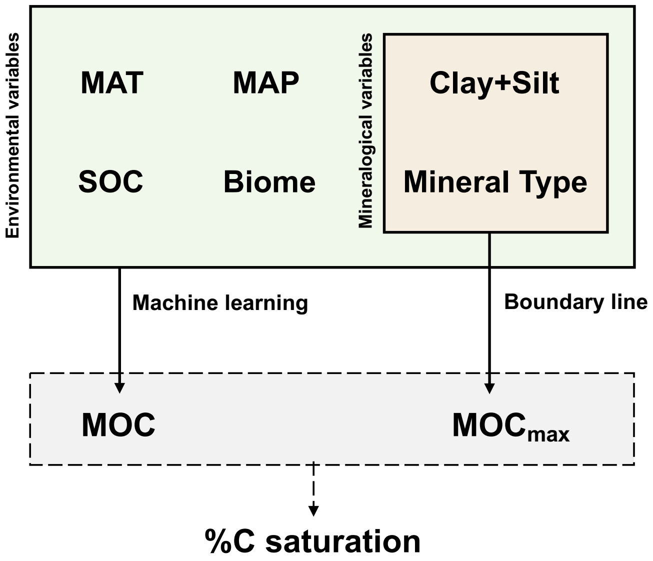

Supplementary Figure 26 | Overview schematic for deriving the spatially-resolved global estimates of mineral-associated organic carbon stocks and mineralogical carbon-storage capacity. Mineral-associated organic carbon (MOC) stocks are an emergent property that depends on climate and vegetation type, as well as the soil mineralogy, and were calculated using a machine-learning approach (see Methods; Fig. 4 and Supplementary Figs. 13-14). In contrast, the mineralogical carbon-storage capacity (MOC_{max}) is a property of the soil mineralogy, and was calculated as a function of the amount and type of soil minerals (see Methods; Fig. 1 and Supplementary Figs. 6-8 for robustness across vegetation types). %C saturation was calculated as MOC/MOC_{max} (as shown in Supplementary Fig. 10).

Supplementary Table 1 | Global carbon stocks summarized by broad land-use categories. Mineral-associated organic carbon (MOC), soil organic carbon (SOC), mineralogical carbon capacity (MOC_{max}), and MOC deficit (to the mineralogical capacity and natural land average environmental limit) totaled over given land-use categories and globally.

	Depth	MOC (Pg C)*	SOC (Pg C)*	MOC_{max} (Pg C)+	MOC deficit relative to MOC_{max} (Pg C)#	MOC deficit relative to natural land average C saturation (Pg C)#
Cropland	topsoil	65 (48, 78)	96	250 ± 24	184 (148, 225)	62 (37, 92)
	subsoil	68 (64, 82)	98	579 ± 56	509 (439, 569)	42 (17, 57)
	total	133 (112, 160)	194	829 ± 80	693 (587, 794)	104 (54, 149)
Grazing	topsoil	143 (100, 172)	214	585 ± 56	440 (356, 539)	155 (97, 226)
	subsoil	137 (120, 172)	201	1237 ± 120	1098 (943, 1234)	98 (40, 137)
	total	280 (220, 344)	415	1822 ± 176	1538 (1299, 1773)	253 (137, 363)
Forestry	topsoil	173 (113, 207)	277	432 ± 44	258 (181, 362)	47 (-9, 129)
	subsoil	183 (147, 208)	289	981 ± 101	796 (670, 933)	3 (-41, 59)
	total	356 (260, 415)	566	1413 ± 145	1054 (851, 1295)	50 (-50, 188)
Global excl. tundra	topsoil	448 (296, 536)	700	1443 ± 141	990 (762, 1281)	286 (127, 509)
	subsoil	451 (372, 538)	701	3153 ± 312	2690 (2293, 3078)	147 (1, 284)
	total	899 (668, 1074)	1401	4596 ± 453	3680 (3055, 4359)	433 (128, 793)
Global incl. tundra	topsoil	570 (335, 679)	892	1857 ± 180	1281 (996, 1684)	380 (182, 696)
	subsoil	595 (424, 698)	933	3915 ± 382	3302 (2823, 3835)	163 (-10, 388)
	total	1165 (760, 1380)	1825	5772 ± 562	4583 (3819, 5519)	543 (172, 1084)

*MOC is given with 5th, 95th quantiles depicting the 90% prediction intervals from the random forest model. SOC is derived as the mean of two global data products (HWSD and SoilGrids; Supplementary Table 2). +MOC_{max} is given with the 90% confidence intervals associated with the mineralogical capacity slopes (Fig. 1). #The MOC deficit at each depth is calculated as the difference between MOC and the mineralogical capacity, MOC_{max}.

$$\text{MOC deficit} = MOC_{max} - MOC$$

The MOC deficit relative to natural land average is the difference between MOC and the environmentally-limited value calculated using the average %C saturation (i.e., average MOC/MOC_{max}, denoted by an overbar below) observed in the MOC synthesis for natural lands at each depth (Fig. 2; i.e., 51 and 19% for topsoil and subsoil, respectively).

$$\text{MOC deficit relative to natural lands} = \overline{\left(\frac{MOC}{MOC_{max}}\right)}_{\substack{natural\\ lands\ in\\ synthesis}} MOC_{max} - MOC$$

We note that this is an average across our MOC synthesis and not all lands may be able to achieve this given current conditions. Parentheses give the 5th, 95th quantiles. Topsoil includes 0-30 cm and subsoil includes 30-100cm. All stock estimates exclude grid cells with >50% organic (Histosol and Gelisol) soils. Global totals are given excluding and including tundra, but tundra is excluded in all other figures and tables. Used lands (cropland, grazing, and forestry) denote grid cells dominated by each respective type of management.

Supplementary Table 2 | Mineral-associated carbon stocks across global soil carbon data products. Mineral-associated organic carbon (MOC), soil organic carbon (SOC), and percent carbon saturation (%C sat; relative to MOC_{max}) excluding tundra, peatlands, and deserts for each global data product.

	HWSD*			SoilGrids+			Averaged data product#		
Depth	MOC (Pg C)	SOC (Pg C)	%C sat.	MOC (Pg C)+	SOC (Pg C)+	%C sat.	MOC (Pg C)	SOC (Pg C)	%C sat.
topsoil	335 (221, 404)	536	31 (18, 41)	533 (330, 677)	867	51 (26, 75)	448 (296, 536)	700	42 (24, 61)
subsoil	313 (241, 430)	507	14 (10, 22)	578 (400, 725)	898	27 (15, 39)	451 (372, 538)	701	21 (14, 30)
total	648 (462, 834)	1043	18 (12, 27)	1111 (730, 1402)	1765	34 (18, 49)	899 (668, 1074)	1401	27 (16, 38)

*SOC stocks from the Harmonized World Soil Database (HWSD) and corresponding MOC stocks with 5th, 95th quantiles depicting the 90% prediction intervals from the random forest model. All stock estimates exclude grid cells with >50% organic (Histosol and Gelisol) soils and grid cells dominated by tundra, peatland, or desert. % C saturation (abbreviated %C sat.) is calculated as MOC divided by MOC_{max} at each grid cell and averaged globally. MOC/SOC ratios averaged 0.68 ± 0.14 (mean ± s.d.) and 0.73 ± 0.21 in topsoils and subsoils, respectively, and 0.70 ± 0.14 across depths globally. +SOC stocks from the SoilGrids data product and corresponding MOC stocks and %C saturation with 5th, 95th quantiles. MOC/SOC ratios averaged 0.65 ± 0.14 and 0.70 ± 0.15 in topsoils and subsoils, respectively, and 0.67 ± 0.13 across depths globally. #SOC stocks derived as the mean of the two global data products (HWSD and SoilGrids) and corresponding MOC stocks and % C saturation with 5th, 95th quantiles. MOC/SOC ratios averaged 0.66 ± 0.13 and 0.70 ± 0.17 in topsoils and subsoils, respectively, and 0.69 ± 0.15 across depths globally. %C saturation in the MOC synthesis averaged 43 ± 2% (± 95% CI on the mean) and 19 ± 3% C in surface and subsurface soils, respectively, and 40 ± 2% across all depths. This most closely matches the global C saturation estimates from the averaged data product, and we focus on this product in all other figures and tables, as a conservative estimate of MOC stocks given the uncertainty and range between SOC data products.

Supplementary Table 3 | Locations, vegetation type, and climate variables of mineral-associated carbon studies. Mean annual temperature (MAT) and mean annual precipitation (MAP) for each study. All studies listed contained measured mineral-associated organic carbon.

Study	Latitude	Longitude	Vegetation	MAT (°C)	MAP (mm yr^{-1})
Amelung 1998 [59]	52.92 N	105.80 W	Grass	0.9	456
	52.19 N	106.17 W	Grass	1.6	343
	50.17 N	107.50 W	Grass	3.2	380
	45.35 N	95.55 W	Grass	6.1	565
	46.50 N	100.54 W	Grass	5	419
	48.33 N	109.41 W	Grass	6.1	300
	44.50 N	105.51 W	Grass	7.2	400
	41.22 N	104.63 W	Grass	8.9	400
	41.43 N	105.70 W	Grass	9	400
	40.10 N	103.13 W	Grass	10.8	375
	40.26 N	99.22 W	Grass	11.6	666
	38.53 N	99.20 W	Grass	12.2	573
	40.48 N	96.42 W	Grass	10.9	792
	39.11 N	96.35 W	Grass	12.4	791
	37.20 N	95.16 W	Grass	14.2	1000
	32.15 N	101.28 W	Grass	17.1	466
	33.06 N	97.21 W	Grass	19.4	865
	29.42 N	96.33 W	Grass	20	1030
	30.05 N	94.06 W	Grass	20.3	1308
	27.45 N	98.04 W	Grass	22.2	700
	27.57 N	98.54 W	Grass	23.4	440
Anderson 1981 [60]	50.00 N	103.50 W	Crop	3	450
Angers 1993 [61]			Crop		
Angers 1991 [62]			Crop		
Balabane 2004 [63]	49.00 N	2.00 E	Crop	10	640
Balesdent 1998 [31]	43.00 N	0.50 E	Forest	13	1200
	43.00 N	0.50 E	Crop	13	1200
Barthes 2008 [64]	4.00 S	13.30 E	Savanna	25	1100
	4.00 S	13.30 E	Crop	25	1100
	4.10 S	13.30 E	Savanna	25	1400
	4.10 S	13.30 E	Crop	25	1400
	6.24 N	2.20 E	Crop	27	1200
	16.00 S	49.30 W	Savanna	23	1500
	16.00 S	49.30 W	Crop	23	1500
	21.22 S	48.03 W	Crop	23	1600
	21.36 S	48.22 W	Crop	23	1500
	21.12 S	47.35 W	Crop	23	1500
	23.23 S	51.11 W	Forest	21	1600
	23.23 S	51.11 W	Crop	21	1600
Bates 1960 [65]	7.40 N	4.00 E	Forest	26.7	1230
Besnard 2001 [66]	49.00 N	4.00 E	Crop	10	620

Bonde 1992 [67]	22.70 S	47.50 W	Forest	21	1250
	22.70 S	47.50 W	Crop	21	1250
Caravaca 1999 [68]	38.00 N	1.00 W	Crop	17	275
	38.00 N	1.00 W	Forest	17	275
Carter 1997, 2003 [69,70]	45.30 N	73.60 W	Crop	6	920
	47.20 N	70.00 W	Crop	4.2	967
	48.50 N	72.40 W	Crop	0.9	866
	45.50 N	64.10 W	Crop	5.6	1107
	45.60 N	67.40 W	Crop	4.4	1143
	42.10 N	82.40 W	Crop	8.7	819
	46.40 N	71.10 W	Crop	4.6	1127
	45.30 N	75.40 W	Crop	5.9	846
	46.20 N	63.10 W	Crop	5.9	1077
	42.50 N	80.30 W	Crop	7.8	935
Catroux 1987 [71]					
Chan 1997, 2001 [72,73]	31.50 S	147.00 E	Crop	18	431
	35.00 S	147.50 E	Crop	16	550
	34.00 S	148.70 E	Crop	15	564
	30.00 S	148.00 E	Crop	19.5	474
	33.00 S	151.00 E	Crop	17	1300
Christensen 1985 [74]	55.00 N	9.00 E	Crop	7.7	790
	55.00 N	9.00 E	Crop	7.9	740
	55.00 N	9.00 E	Crop	8.4	700
	55.00 N	9.00 E	Crop	8.1	700
Christensen 1986 [75]	56.00 N	9.00 E	Crop	8	750
	55.00 N	9.00 E	Crop	8	750
Christensen 1987 [76]	56.00 N	9.00 E	Crop	8	750
	55.00 N	10.00 E	Crop	8	750
Dalal 1986a-c, 1987a-b [77–81]	27.00 S	151.00 E	Grass	18.5	670
	27.00 S	151.00 E	Forest	19.4	630
	27.00 S	151.00 E	Forest	18.5	670
	29.00 S	150.00 E	Forest	19.9	610
	29.00 S	149.00 E	Forest	20.5	480
	27.00 S	149.00 E	Forest	20.3	580
Doetterl 2015 [41]	30.20 S	71.25 W	Savanna	15.2	98
	31.81 S	71.50 W	Savanna	16.4	208
	32.88 S	70.52 W	Savanna	14.6	349
	34.00 S	71.14 W	Savanna	16.6	450
	33.48 S	70.83 W	Savanna	13.8	401
	33.34 S	71.61 W	Savanna	15.1	580
	33.97 S	71.88 W	Grass	16.9	568
	36.46 S	71.70 W	Grass	12	1321
	37.25 S	73.27 W	Grass	13.4	1431
	38.77 S	73.39 W	Grass	13	1205
	39.86 S	72.11 W	Grass	11	2108
	39.93 S	73.41 W	Grass	11.6	2174
	40.90 S	73.15 W	Grass	10.9	1456
	42.42 S	73.82 W	Grass	9.9	2233

	43.06 S	73.62 W	Grass	10.8	2232
	43.96 S	72.40 W	Grass	10.3	2308
	45.49 S	72.81 W	Grass	6.7	2120
	45.79 S	72.91 W	Grass	3.2	1524
	46.57 S	72.61 W	Grass	7	1048
	51.81 S	72.16 W	Grass	6.5	394
	53.43 S	70.99 W	Grass	6.2	620
	53.31 S	70.36 W	Grass	6.3	483
	62.16 S	58.47 W	Grass	-2.9	797
	62.65 S	61.08 W	Grass	-2.3	648
Elustondo 1990 [82]	45.50 N	73.00 W	Crop	4	900
	46.00 N	72.50 W	Crop	4	900
	46.00 N	73.50 W	Crop	4	900
	46.00 N	72.60 W	Crop	4	900
	46.00 N	72.00 W	Crop	4	900
	45.80 N	73.00 W	Crop	4	900
	48.00 N	71.00 W	Crop	4	900
	45.50 N	73.00 W	Grass	4	900
	46.00 N	72.50 W	Grass	4	900
	46.00 N	73.50 W	Grass	4	900
	46.00 N	72.60 W	Grass	4	900
	46.00 N	72.00 W	Grass	4	900
	45.80 N	73.00 W	Grass	4	900
	48.00 N	71.00 W	Grass	4	900
Feller 1991, 1996, 1997 [83–85]	14.50 N	14.50 W	Grass	29	700
	14.50 N	14.50 W	Crop	29	700
	14.50 N	14.50 W	Savanna	29	700
	14.50 N	14.50 W	Savanna	29	800
	14.50 N	14.50 W	Crop	29	800
	7.50 N	5.50 W	Forest	26	1360
	7.50 N	5.50 W	Savanna	26	1360
	7.50 N	5.50 W	Crop	26	1360
	8.50 N	1.00 E	Forest	27	1040
	8.50 N	1.00 E	Savanna	27	1040
	8.50 N	1.00 E	Crop	27	1040
	16.30 N	61.50 W	Grass	25	3000
	16.30 N	61.50 W	Crop	25	3000
	14.00 N	61.00 W	Grass	25	2700
	14.00 N	61.00 W	Crop	25	2700
	14.50 N	61.00 W	Forest	26	1820
	14.50 N	61.00 W	Savanna	27	1200
	14.50 N	61.00 W	Grass	27	1200
	14.50 N	61.00 W	Crop	27	1200
	3.50 S	62.00 W	Forest	21	1200
	3.50 S	62.00 W	Crop	21	1200
	3.50 S	62.00 W	Grass	18	1530
	3.50 S	62.00 W	Crop	18	1530
Feng 2014 [86]	43.50 N	124.80 E	Crop	4.5	525

	34.78 N	113.67 E	Crop	14	645
	26.75 N	111.87 E	Crop	18	1250
	36.90 N	120.70 E	Crop	11.2	779
	52.82 N	104.60 E	Crop	0.6	410
	52.10 N	114.20 E	Crop	0.7	373
Guggenberger 1994 [87]	43.53 N	11.48 E	Forest	6.5	1500
	43.53 N	11.48 E	Grass	6.5	1500
	43.53 N	11.48 E	Crop	6.5	1500
Guggenberger 1995 [88]	4.37 N	71.19 W	Savanna	26	2200
	5.37 N	72.19 W	Grass	26	2200
	6.37 N	73.19 W	Grass	26	2200
Guibert 1999 [89]	8.70 N	16.60 E	Crop	27	1000
	6.20 N	6.00 W	Crop	26	1500
Hassink 1997 [90,91]	53.00 N	6.00 E	Grass	8	750
	51.20 N	5.60 E	Grass	8	750
	52.40 N	6.20 E	Grass	8	750
	52.30 N	6.40 E	Grass	8	750
	53.00 N	6.60 E	Grass	8	750
	52.00 N	5.60 E	Grass	8	750
	52.20 N	6.50 E	Grass	8	750
	53.20 N	7.00 E	Grass	8	750
	52.50 N	5.50 E	Grass	8	750
	53.30 N	6.30 E	Grass	8	750
	53.30 N	6.50 E	Grass	8	750
	51.80 N	5.30 E	Grass	8	750
	51.20 N	5.60 E	Crop	8	750
	53.00 N	6.60 E	Crop	8	750
	53.20 N	6.60 E	Crop	8	750
ISRAD [92]	25.20 S to 45.57 N	122.50 W to 31.55 E	Forest, Grass, Shrub, Savanna, Crop	5.7 to 26.5	410 to 2585
Jandl 2004 [93]	51.40 N	11.80 E	Crop	9	500
Kahle 2002, 2003 [94,95]	51.50 N	11.97 E	Grass	9.1	484
	51.50 N	11.97 E	Crop	9.1	484
Kölbl 2006 [96]	48.50 N	11.50 E	Crop	7.4	830
Larre-Larrouy 2003 [97]	14.25 N	60.53 W	Crop	26	1580
Leinweber 1992 [98]	54.00 N	12.00 E	Crop	8.5	590
Li 2009 [99]	39.14 N	99.84 E	Grass	7.6	79
	39.14 N	99.84 E	Crop	7.6	79
Liang 2009 [100]	45.00 N	127.50 E	Grass	2.75	550
	45.00 N	127.50 E	Crop	2.75	550
LUCAS [5,8]	36.68 N to 66.51 N	9.25 W to 30.57 E	Forest, Grass, Shrub, Crop	-0.1 to 18	213 to 1603
Matus 2008 [101]			Forest		

			Crop		
			Crop	15.9	512
			Crop	13.8	1087
			Crop	14.7	776
			Crop	15.4	750
McKeague 1971 [102]	52.50 N	111.00 W	Grass	1.5	410
	53.00 N	112.00 W	Grass	1.5	410
	49.20 N	98.40 W	Grass	2.5	470
	53.50 N	113.00 W	Forest	1.5	410
	43.00 N	80.30 W	Forest	7.7	900
	43.50 N	80.20 W	Forest	7	850
	43.50 N	80.20 W	Forest	6.6	900
	43.50 N	80.20 W	Grass	7	850
	44.00 N	78.50 W	Grass	7	800
	52.00 N	106.00 W	Grass	3.5	385
	49.70 N	103.90 W	Grass	3.5	385
	49.20 N	102.20 W	Grass	2.8	400
Nacro 1996 [103]	6.13 N	5.20 W	Savanna	27	1200
Pellegrini 2021 & unpub. [104]	36.61 N	118.72 W	Forest	5.2	775
Pineiro 2009 [105]	32.00 S	57.08 W	Grass	17.3	1406
	31.50 S	58.17 W	Grass	18.9	1300
	33.52 S	55.33 W	Grass	16.3	1161
	33.19 S	56.58 W	Grass	17.4	1099
	36.30 S	58.30 W	Grass	14.9	861
Schmidt 2002 [106]	50.90 N	8.00 E	Forest	8	570
Schöning 2005a-b [107,108]	41.52 N	13.38 E	Forest	11	875
	48.40 N	7.05 E	Forest	9.2	744
	55.29 N	11.38 E	Forest	8.1	602
	51.20 N	10.22 E	Forest	7.6	747
Schöning 2006 [109]	48.40 N	7.05 E	Forest	9.2	744
	51.20 N	10.22 E	Forest	7.6	747
	50.56 N	13.29 E	Forest	8.3	624
Schulten 1993 [110]	45.20 N	74.40 W	Crop	6	860
Shang 1997 [111]			Forest		
			Crop		
Shrestha 2007 [112]			Crop		
			Forest		
Sleutel 2006 [113]	51.10 N	3.30 E	Crop	10	680
	51.00 N	3.00 E	Crop	10	680
	51.00 N	3.10 E	Crop	10	680
	51.00 N	2.70 E	Crop	10	680
	50.80 N	3.30 E	Crop	10	680
Sleutel 2006 [114]	47.32 N	19.00 E	Crop	10.3	453
	46.46 N	17.00 E	Crop	10	623
Solomon 2000 [115]			Forest		
			Crop		
Soong 2020 & unpub. [116]	5.38 N	53.76 W	Forest	25.7	2460
	5.23 N	54.10 W	Forest	25.7	2480

	4.57 N	52.01 W	Forest	25.5	3806
	5.35 N	53.49 W	Forest	26.0	2395
	4.27 N	52.26 W	Forest	25.5	3591
	4.23 N	52.43 W	Forest	25.5	3320
	5.31 N	52.97 W	Forest	25.9	2809
	4.37 N	52.20 W	Forest	25.5	3758
	4.41 N	52.23 W	Forest	25.5	3765
	4.60 N	52.10 W	Forest	25.5	3719
	4.63 N	52.26 W	Forest	25.5	3789
Steffens 2009 [117]	43.38 N	116.42 E	Grass	0.7	343
Stemmer 1999 [118]			Crop		
Thuriès 2000 [119]	42.60 N	3.00 E	Crop	15.6	581
Tiessen 1983 [120]			Grass		
			Crop		
Turchenek 1979 [121]	35.00 S	138.60 E	Grass	16.5	540
	35.00 S	138.60 E	Crop	16.5	540
	37.50 S	140.40 E	Crop	16.5	540
	52.80 N	104.60 W	Crop	0.6	410
Young 1979 [122]			Grass		
			Forest		
			Crop		
Zhang 1988 [123]			Grass		
			Crop		
Zhao 2006 [124]	42.57 N	128.57 E	Crop	6.1	570
	43.21 N	124.19 E	Crop	5.8	577
	43.21 N	125.37 E	Crop	4.6	603
	42.03 N	126.10 E	Crop	3.9	747
	42.27 N	126.48 E	Crop	3.7	720
	44.01 N	126.51 E	Crop	3.4	709
	44.52 N	126.30 E	Crop	4	500
Zinn 2007 [125]			Forest		

Supplementary Table 4 | Accrual rates and mineralogical carbon saturation of carbon accrual studies. Soil order and vegetation type given for each site.

Study	Soil Order	Vegetation type	Clay + Silt (%)	C saturation (%)	Duration (years)	Carbon accrual rate (t C ha^{-1} yr^{-1})
Akala 2001 [126]	Entisols	Pasture	90	5	5	2.0
	Entisols	Pasture	90	6	5	2.7
	Entisols	Pasture	90	8	5	2.8
	Entisols	Pasture	90	11	5	2.5
	Entisols	Pasture	90	16	5	5.2
	Entisols	Forest	90	6	4	-0.7
	Entisols	Forest	90	5	6	2.0
	Entisols	Forest	90	7	5	2.9
	Entisols	Forest	90	13	6	2.8
Alcántara 2016 [127]	Alfisols	Cropland	95	5	48	0.4
Babujia 2010 [128]	Oxisols	Cropland	79	38	20	1.1
Black 2009 [129]	Inceptisols/ Mollisols	Grassland to Forest	73	39	9	2.2
	Inceptisols/ Mollisols	Grassland to Forest	77	43	21	-0.6
	Inceptisols/ Mollisols	Grassland to Forest	83	38	15	-0.1
Boddey 2010 [130]	Oxisols	Cropland	83	38	15	0.6
Bowden 2014 [131]	Alfisols	Forest	25	87	19	0.2
Brown 1990 [132]	Ultisols	Cropland to Forest	95	11	22.5	0.5
	Ultisols	Cropland to Forest	89	12	42.5	0.7
	Ultisols	Cropland to Forest	95	11	10	1.3
	Entisols	Cropland to Forest	84	29	35	0.9
	Entisols	Cropland to Forest	84	24	50	0.9
Carvalho 2010 [133]	Oxisols	Cropland	83	42	5	1.6
	Oxisols	Cropland	81	59	8	1.5
Chatterjee 2009 [134]	Alfisols	Grassland	61	6	8	3.7
	Entisols	Grassland	75	19	21	0.8

	Ultisols	Forest	66	19	11	2.0
Dick 1998 [135]	Alfisols	Cropland	88	18	30	0.4
	Alfisols	Cropland	88	24	30	0.7
	Alfisols	Cropland	88	18	30	1.0
Fekete unpub. [136]	Inceptisols	Forest	58	58	10	0.8
	Inceptisols	Forest	58	58	10	0.4
Freixo 2002 [137]	Oxisols	Cropland	80	45	11	0.0
Ganjegunte 2009 [138]	Aridisols	Grassland	31	15	5	-0.7
	Aridisols	Grassland	30	12	6	-0.8
	Aridisols	Pasture	68	6	13	0.7
	Aridisols	Pasture	73	10	12	0.6
Harden unpub., Doetterl 2018 [92,139]	Entisols	Grassland	78	16	21	1.3
	Alfisols	Grassland	70	5	21	0.5
Jantalia 2007 [140]	Oxisols	Cropland	61	67	16	0.5
Jenkinson 1971 [141]	Alfisols	Cropland to Forest	50	15	23	0.4
	Alfisols	Cropland to Forest	50	16	60	0.4
	Alfisols	Cropland to Forest	47	19	21	0.4
	Alfisols	Cropland to Forest	47	20	61	0.1
Keskin 2009 [142]	Entisols	Forest	41	0	17	2.2
	Entisols	Forest	33	0	17	1.3
Lajtha 2014 [143]	Inceptisols	Forest	35	62	20	-0.2
Lawrence et al. unpub.	Mollisols	Grassland	31	50	10	0.0
	Mollisols	Grassland	56	32	10	-0.8
	Mollisols	Grassland	46	35	10	0.1
	Inceptisols	Grassland	55	58	12	0.3
Lilienfein 2003, Sollins 1983 [144,145]	Entisols	Forest	14	18	22	0.0
	Entisols	Forest	15	48	22	-0.6

	Entisols	Forest	14	107	22	-0.2
	Entisols	Forest	18	115	22	0.0
Lorenz 2007 [146]	Entisols	Forest	56	24	17	0.9
	Entisols	Forest	56	4	30	1.2
Marchão 2009 [147]	Oxisols	Cropland	69	55	13	0.5
	Oxisols	Cropland	69	62	13	-0.5
Mazzoncini 2011 [148]	Entisols	Cropland	56	17	15	0.1
	Entisols	Cropland	56	17	15	0.3
	Entisols	Cropland	56	17	15	0.3
	Entisols	Cropland	56	17	15	0.4
Nadelhoffer unpub. [136]	Spodosols	Forest	7	56	5	1.5
Nii-Annang 2009 [149]	Entisols	Forest	25	9	9	1.8
	Entisols	Forest	25	9	9	2.0
	Entisols	Forest/ Cropland	25	9	9	1.7
	Entisols	Cropland	25	9	9	0.8
Pierson 2021[136,150]	Andisols/ Inceptisols	Forest	43	76	20	0.4
	Andisols/ Inceptisols	Forest	43	76	20	0.5
Quinkenstein 2011 [151]	Entisols	Forest	35	6	2	4.2
	Entisols	Forest	38	10	10	3.9
Raiesi 2012 [152]	Alfisols	Cropland to Grassland	86	6	20	0.3
	Alfisols	Cropland to Grassland	86	7	20	0.3
Reintam et al. 2002 [153]	Entisols	Forest	36	0	29	0.2
	Entisols	Forest	36	0	31	0.3
	Entisols	Forest	36	0	31	1.7
	Entisols	Forest	36	0	34	1.6
Roscoe 2003 [154]	Oxisols	Cropland	90	24	10	0.0
Schipper 2011 [155]	Andisols	Scrubland to Pasture	35	60	25	0.4

	Andisols	Scrubland to Pasture	93	51	17	1.3
	Andisols	Scrubland to Pasture	35	87	65	-0.2
	Andisols	Scrubland to Pasture	56	53	27	0.7
	Andisols	Scrubland to Pasture	56	46	18	0.9
	Ultisols	Scrubland to Pasture	68	54	28	0.3
	Alfisols	Scrubland to Pasture	79	27	43	0.5
	Alfisols	Scrubland to Pasture	79	26	19	1.0
	Oxisols	Scrubland to Pasture	90	91	44	0.4
Sever 2009 [156]	Entisols	Forest	45	0	17	2.2
Shrestha 2009 [157]	Entisols	Grassland	62	9	5	2.6
	Ultisols	Grassland	73	15	5	0.8
	Ultisols	Grassland	73	15	5	2.8
	Ultisols	Grassland	73	15	5	0.5
	Ultisols	Grassland	73	15	5	2.5
	Entisols	Grassland	62	9	5	2.6
	Entisols	Grassland	62	9	5	3.1
	Entisols	Grassland	62	9	5	2.7
	Entisols	Grassland	62	9	5	3.2
	Alfisols	Grassland	48	17	5	0.7
	Alfisols	Grassland	48	17	5	2.5
	Alfisols	Grassland	48	17	5	0.4
	Alfisols	Grassland	48	17	5	2.1
Williams-Linera 1983 [158]	Alfisols	Cropland to Forest	76	24	7	5.1

Supplementary References:

1. Schmidt, M. W. I. *et al.* Persistence of soil organic matter as an ecosystem property. *Nature* **478**, 49–56 (2011).

2. Lehmann, J. *et al.* Persistence of soil organic carbon caused by functional complexity. *Nat. Geosci.* **13**, 529–534 (2020).

3. Amelung, W. *et al.* Towards a global-scale soil climate mitigation strategy. *Nat. Commun.* **11**, 1–10 (2020).

4. Chenu, C. *et al.* Increasing organic stocks in agricultural soils: Knowledge gaps and potential innovations. *Soil Tillage Res.* **188**, 41–52 (2019).

5. Cotrufo, M. F., Ranalli, M. G., Haddix, M. L., Six, J. & Lugato, E. Soil carbon storage informed by particulate and mineral-associated organic matter. *Nat. Geosci.* **12**, 989–994 (2019).

6. Lavallee, J. M., Soong, J. L. & Cotrufo, M. F. Conceptualizing soil organic matter into particulate and mineral-associated forms to address global change in the 21st century. *Glob. Chang. Biol.* **26**, 261–273 (2020).

7. Benbi, D. K., Boparai, A. K. & Brar, K. Decomposition of particulate organic matter is more sensitive to temperature than the mineral associated organic matter. *Soil Biol. Biochem.* **70**, 183–192 (2014).

8. Lugato, E., Lavallee, J. M., Haddix, M. L., Panagos, P. & Cotrufo, M. F. Different climate sensitivity of particulate and mineral-associated soil organic matter. *Nat. Geosci.* **14**, 295–300 (2021).

9. Viscarra Rossel, R. A. *et al.* Continental-scale soil carbon composition and vulnerability modulated by regional environmental controls. *Nat. Geosci.* **12**, 547–552 (2019).

10. Plaza, C. *et al.* Direct observation of permafrost degradation and rapid soil carbon loss in tundra. *Nat. Geosci.* **12**, 627–631 (2019).

11. Janzen, H. H. The soil carbon dilemma: Shall we hoard it or use it? *Soil Biol. Biochem.* **38**, 419–424 (2006).

12. Bossio, D. A. *et al.* The role of soil carbon in natural climate solutions. *Nat. Sustain.* **3**, 391–398 (2020).

13. Lehmann, J., Bossio, D. A., Kögel-Knabner, I. & Rillig, M. C. The concept and future prospects of soil health. *Nat. Rev. Earth Environ.* **1**, 544–553 (2020).

14. Bradford, M. A. *et al.* Soil carbon science for policy and practice. *Nat. Sustain.* **2**, 1070–1072 (2019).

15. Spohn, M. Increasing the organic carbon stocks in mineral soils sequesters large amounts of phosphorus. *Glob. Chang. Biol.* **26**, 4169–4177 (2020).

16. Smith, P. Soil carbon sequestration and biochar as negative emission technologies. *Glob. Chang.*

Biol. **22**, 1315–1324 (2016).

17. Davies, C. A., Robertson, A. D. & McNamara, N. P. The importance of nitrogen for net carbon sequestration when considering natural climate solutions. *Glob. Chang. Biol.* 218–219 (2020). doi:10.1111/gcb.15381

18. Stewart, C. E., Paustian, K., Conant, R. T., Plante, A. F. & Six, J. Soil carbon saturation: Concept, evidence and evaluation. *Biogeochemistry* **86**, 19–31 (2007).

19. Castellano, M. J., Mueller, K. E., Olk, D. C., Sawyer, J. E. & Six, J. Integrating plant litter quality, soil organic matter stabilization, and the carbon saturation concept. *Glob. Chang. Biol.* **21**, 3200–3209 (2015).

20. Six, J., Conant, R. T., Paul, E. A. & Paustian, K. Stabilization mechanisms of soil organic matter: Implications for C-saturation of soils. *Plant Soil* **241**, 155–176 (2002).

21. Gulde, S., Chung, H., Amelung, W., Chang, C. & Six, J. Soil Carbon Saturation Controls Labile and Stable Carbon Pool Dynamics. *Soil Sci. Soc. Am. J.* **72**, 605 (2008).

22. Hemingway, J. D. *et al.* Mineral protection regulates long-term global preservation of natural organic carbon. *Nature* **570**, 228–231 (2019).

23. Cotrufo, M. F., Wallenstein, M. D., Boot, C. M., Denef, K. & Paul, E. The Microbial Efficiency-Matrix Stabilization (MEMS) framework integrates plant litter decomposition with soil organic matter stabilization: Do labile plant inputs form stable soil organic matter? *Glob. Chang. Biol.* **19**, 988–995 (2013).

24. Kögel-Knabner, I. *et al.* Organo-mineral associations in temperate soils: Integrating biology, mineralogy, and organic matter chemistry. *J. Plant Nutr. Soil Sci.* **171**, 61–82 (2008).

25. Poeplau, C. *et al.* Isolating organic carbon fractions with varying turnover rates in temperate agricultural soils – A comprehensive method comparison. *Soil Biol. Biochem.* **125**, 10–26 (2018).

26. Kleber, M. *et al.* Mineral-organic associations: formation, properties, and relevance in soil environments. *Adv. Agron.* **130**, 1–140 (2015).

27. Villarino, S. H., Pinto, P., Jackson, R. B. & Piñeiro, G. Plant rhizodeposition: A key factor for soil organic matter formation in stable fractions. *Sci. Adv.* **7**, 1–14 (2021).

28. Sokol, N. W. & Bradford, M. A. Microbial formation of stable soil carbon is more efficient from belowground than aboveground input. *Nat. Geosci.* **12**, 46–53 (2019).

29. Kallenbach, C. M., Frey, S. D. & Grandy, A. S. Direct evidence for microbial-derived soil organic matter formation and its ecophysiological controls. *Nat. Commun.* **7**, 1–10 (2016).

30. Soong, J. L. *et al.* Five years of whole-soil warming led to loss of subsoil carbon stocks and increased CO2 efflux. *Sci. Adv.* 1–9 (2021).

31. Balesdent, J., Besnard, E., Arrouays, D. & Chenu, C. The dynamics of carbon in particle-size fractions of soil in a forest-cultivation sequence. *Plant Soil* **201**, 49–57 (1998).

32. Balesdent, J. The significance of organic separates to carbon dynamics and its modelling in some

cultivated soils. *Eur. J. Soil Sci.* **47**, 485–493 (1996).

33. Sokol, N. W., Sanderman, J. & Bradford, M. A. Pathways of mineral-associated soil organic matter formation: Integrating the role of plant carbon source, chemistry, and point of entry. *Glob. Chang. Biol.* **25**, 12–24 (2019).

34. Fontaine, S. *et al.* Stability of organic carbon in deep soil layers controlled by fresh carbon supply. *Nature* **450**, 277–280 (2007).

35. Ahrens, B., Braakhekke, M. C., Guggenberger, G., Schrumpf, M. & Reichstein, M. Contribution of sorption, DOC transport and microbial interactions to the ^{14}C age of a soil organic carbon profile: Insights from a calibrated process model. *Soil Biol. Biochem.* **88**, 390–402 (2015).

36. Ahrens, B. *et al.* Combination of energy limitation and sorption capacity explains 14C depth gradients. *Soil Biol. Biochem.* **148**, (2020).

37. Balesdent, J. *et al.* Renouvellement du carbone profond des sols cultivés: une estimation par compilation de données isotopiques. *Biotechnol. Agron. Soc. Environ.* (2017).

38. Roper, M. M. *et al.* Allocation into soil organic matter fractions of 14C captured via photosynthesis by two perennial grass pastures. *Soil Res.* **51**, 748–759 (2013).

39. West, T. O. & Six, J. Considering the influence of sequestration duration and carbon saturation on estimates of soil carbon capacity. *Clim. Change* **80**, 25–41 (2007).

40. Chenu, C. Soil phases: The organic solid phase. *Soils Basic Concepts Futur. Challenges* **9780521851**, 45–56 (2006).

41. Doetterl, S. *et al.* Soil carbon storage controlled by interactions between geochemistry and climate. *Nat. Geosci.* **8**, 780–783 (2015).

42. Robertson, A. D. *et al.* Unifying soil organic matter formation and persistence frameworks: The MEMS model. *Biogeosciences* **16**, 1225–1248 (2019).

43. Sulman, B. N. *et al.* Multiple models and experiments underscore large uncertainty in soil carbon dynamics. *Biogeochemistry* **141**, 109–123 (2018).

44. Abramoff, R. *et al.* The Millennial model: in search of measurable pools and transformations for modeling soil carbon in the new century. *Biogeochemistry* **137**, 51–71 (2018).

45. Wieder, W. R., Sulman, B. N., Hartman, M. D., Koven, C. D. & Bradford, M. A. Arctic Soil Governs Whether Climate Change Drives Global Losses or Gains in Soil Carbon. *Geophys. Res. Lett.* **46**, 14486–14495 (2019).

46. Wieder, W. R. *et al.* Carbon cycle confidence and uncertainty: Exploring variation among soil biogeochemical models. *Glob. Chang. Biol.* **24**, 1563–1579 (2018).

47. Whittaker, R. *Communities and ecosystems.* (MacMillan, New York, 1975).

48. Batjes, N. H. *et al.* WoSIS: Providing standardised soil profile data for the world. *Earth Syst. Sci. Data* **9**, 1–14 (2017).

49. Stewart, C. E., Paustian, K., Conant, R. T., Plante, A. F. & Six, J. Soil carbon saturation: Implications for measurable carbon pool dynamics in long-term incubations. *Soil Biol. Biochem.* **41**, 357–366 (2009).

50. Kirchner, J. W. *et al.* Streamflow response to forest management. *Nature* **578**, E12–E15 (2020).

51. Ploton, P. *et al.* Spatial validation reveals poor predictive performance of large-scale ecological mapping models. *Nat. Commun.* **11**, 1–11 (2020).

52. Ma, H. *et al.* The global distribution and environmental drivers of aboveground versus belowground plant biomass. *Nat. Ecol. Evol.* (2021). doi:10.1038/s41559-021-01485-1

53. Friedl, M. A. *et al.* MODIS Collection 5 global land cover: Algorithm refinements and characterization of new datasets. *Remote Sens. Environ.* **114**, 168–182 (2010).

54. Shi, Z. *et al.* The age distribution of global soil carbon inferred from radiocarbon measurements. *Nat. Geosci.* **13**, 555–559 (2020).

55. Harden, J. W. *et al.* Networking our science to characterize the state, vulnerabilities, and management opportunities of soil organic matter. *Glob. Chang. Biol.* **24**, e705–e718 (2018).

56. Erb, K. H. *et al.* A comprehensive global 5 min resolution land-use data set for the year 2000 consistent with national census data. *J. Land Use Sci.* **2**, 191–224 (2007).

57. Post, W. M. & Kwon, K. C. Soil carbon sequestration and land-use change: Processes and potential. *Glob. Chang. Biol.* **6**, 317–327 (2000).

58. Vesterdal, L., Clarke, N., Sigurdsson, B. D. & Gundersen, P. Do tree species influence soil carbon stocks in temperate and boreal forests? *For. Ecol. Manage.* **309**, 4–18 (2013).

59. Amelung, W. *et al.* Carbon, nitrogen, and sulfur pools in particle-size fractions as influenced by climate. *Soil Sci. Soc. Am. J.* **62**, 172–181 (1998).

60. Anderson, D. W., Saggar, S., Bettany, J. R. & Stewart, J. W. B. Particle Size Fractions and Their Use in Studies of Soil Organic Matter: I. The Nature and Distribution of Forms of Carbon, Nitrogen, and Sulfur. *Soil Sci. Soc. Am. J.* **45**, 767 (1981).

61. Angers, D. A., N'dayegamiye, A. & Cote, D. Tillage-induced differences in organic matter of particle-size fractions and microbial biomass. *Soil Sci. Soc. Am. J.* **57**, 512–516 (1993).

62. Angers, D. A. & N'Dayegamiye, A. Effects of manure application on carbon, nitrogen, and carbohydrate contents of a silt loam and its particle-size fractions. *Biol. Fertil. Soils* **11**, 79–82 (1991).

63. Balabane, M. & Plante, A. F. Aggregation and carbon storage in silty soil using physical fractionation techniques. *Eur. J. Soil Sci.* **55**, 415–427 (2004).

64. Barthès, B. G. *et al.* Texture and sesquioxide effects on water-stable aggregates and organic matter in some tropical soils. *Geoderma* **143**, 14–25 (2008).

65. Bates, J. A. . Studies on a Nigerian Forest Soil: I. The distribution of organic matter in the profile and in various soil fractions. *J. Soil Sci.* **11**, 257–265 (1960).

66. Besnard, E., Chenu, C. & Robert, M. Influence of organic amendments on copper distribution among particle-size and density fractions in Champagne vineyard soils. *Environ. Pollut.* **112**, 329–337 (2001).

67. Bonde, T. A., Christensen, B. T. & Cerri, C. C. Dynamics of soil organic matter as reflected by natural 13C abundance in particle size fractions of forested and cultivated oxisols. *Soil Biol. Biochem.* **24**, 275–277 (1992).

68. Caravaca, F., Lax, A. & Albaladejo, J. Organic matter, nutrient contents and cation exchange capacity in fine fractions from semiarid calcareous soils. *Geoderma* **93**, 161–176 (1999).

69. Carter, M. R., Angers, D. A., Gregorich, E. G. & Bolinder, M. A. Characterizing organic matter retention for surface soils in eastern Canada using density and particle size fractions. *Can. J. Soil Sci.* **83**, 11–23 (2003).

70. Carter, M. R., Angers, D. A., Gregorich, E. G. & Bolinder, M. A. Organic carbon and nitrogen stocks and storage profiles in cool, humid soils of eastern Canada. *Can. J. Soil Sci.* **77**, 205–210 (1997).

71. Catroux, G. & Schnitzer, M. Chemical, Spectroscopic, and Biological Characteristics of the Organic Matter in Particle Size Fractions Separated from an Aquoll. *Soil Sci. Soc. Am. J.* **51**, 1200–1207 (1987).

72. Chan, K. Y. Soil particulate organic carbon under different land use and management. *Soil Use Manag.* **17**, 217–221 (2001).

73. Chan, K. Y. Consequences of changes in particulate organic carbon in vertisols under pasture and cropping. *Soil Sci. Soc. Am. J.* **61**, 1376–1382 (1997).

74. Christensen, B. T. Carbon and Nitrogen in Particle Size Fractions Isolated from Danish Arable Soils by Ultrasonic Dispersion and Gravity-Sedimentation. *Acta Agric. Scand.* **35**, 175–187 (1985).

75. Christensen, B. T. Straw incorporation and soil organic matter in macro-aggregates and particle size separates. *J. Soil Sci.* **37**, 125–135 (1986).

76. Christensen, B. T. Decomposability of organic matter in particle size fractions from field soils with straw incorporation. *Soil Biol. Biochem.* **19**, 429–435 (1987).

77. Dalal, R. C. & Mayer, R. J. Long-term trends in fertility of soils under continuous cultivation and cereal cropping in southern Queensland. I. Overall Changes in Soil Properties and Trends in Winter Cereal Yields. *Aust. J. Soil Res.* **24**, 265–279 (1986).

78. Dalal, R. C. & Mayer, R. J. Long-term trends in fertility of soils under continuous cultivation and cereal cropping in southern Queensland. III: Distribution and Kinetics of Soil Organic Carbon in Particle-size Fractions. *Aust. J. Soil Res.* **24**, 293–300 (1986).

79. Dalal, R. C. & Mayer, R. J. Long-term trends in fertility of soils under continuous cultivation and cereal cropping in southern Queensland. IV. Loss of Organic Carbon from Different Density Functions. *Aust. J. Soil Res.* **24**, 301–309 (1986).

80. Dalal, R. C. & Mayer, R. J. Long-term trends in fertility of soils under continuous cultivation and cereal cropping in southern Queensland. VI. Loss of Total Nitrogen from Different Particle-size and Density Fractions. *Aust. J. Soil Res.* **25**, 83–93 (1987).

81. Dalal, R. C. & Mayer, R. J. Long-term trends in fertility of soils under continuous cultivation and cereal cropping in southern Queensland. VII. Dynamics of Nitrogen Mineralization Potentials and Microbial Biomass. *Aust. J. Soil Res.* **25**, 461–472 (1987).

82. Elustondo, J., Angers, D. A., Laverdière, M. R. & N'Dayegamiye, A. Étude comparative de l'agrégation et de la matière organique associée aux fractions granulométriques de sept sols sous culture de maïs ou en prairie. *Can. J. Soil Sci.* **70**, 395–402 (1990).

83. Feller, C., Albrecht, A. & Tessier, D. *Aggregation and organic matter storage in kaolinitic and smectitic tropical soils*. *In: Structure and Organic Matter Storage in Agricultural Soils. Eds. M R Carter and B A Stewart.* (1996).

84. Feller, C. & Beare, M. H. Physical control of soil organic matter dynamics in the tropics. *Geoderma* **79**, 69–116 (1997).

85. Feller, C., Fritsch, E., Poss, R. & Valentin, C. Effet de la texture sur le stockage et la dynamique des matières organiques dans quelques sols ferrugineux et ferrallitiques (Afrique de l'Ouest, en particulier). *Cah. Orstom, sér. Pédol.* **26**, 25–36 (1991).

86. Feng, W., Plante, A. F., Aufdenkampe, A. K. & Six, J. Soil organic matter stability in organo-mineral complexes as a function of increasing C loading. *Soil Biol. Biochem.* **69**, 398–0405 (2014).

87. Guggenberger, G., Christensen, B. T. & Zech, W. Land-use effects on the composition of organic matter in particle-size separates of soil: I. Lignin and carbohydrate signature. *Eur. J. Soil Sci.* **45**, 449–458 (1994).

88. Guggenberger, G., Zech, W., Haumaier, L. & Christensen, B. T. Land-use effects on the composition of organic matter in particle-size separates of soils: II. CPMAS and solution 13C NMR analysis. *Eur. J. Soil Sci.* **46**, 147–158 (1995).

89. Guibert, H., Fallavier, P. & Roméro, J. J. Carbon content in soil particle size and consequence on cation exchange capacity of alfisols. *Commun. Soil Sci. Plant Anal.* **30**, 2521–2537 (1999).

90. Hassink, J., Whitmore, A. P. & Kubát, J. Size and density fractionation of soil organic matter and the physical capacity of soils to protect organic matter. *Dev. Crop Sci.* **25**, 245–255 (1997).

91. Hassink, J. The capacity of soils to preserve organic C and N by their association with clay and silt particles. *Plant Soil* **191**, 77–87 (1997).

92. Lawrence, C. R. *et al.* An open-source database for the synthesis of soil radiocarbon data: International Soil Radiocarbon Database (ISRaD) version 1.0. *Earth Syst. Sci. Data* **12**, 61–76 (2020).

93. Jandl, G., Leinweber, P., Schulten, H. R. & Eusterhues, K. The concentrations of fatty acids in organo-mineral particle-size fractions of a Chernozem. *Eur. J. Soil Sci.* **55**, 459–469 (2004).

94. Kahle, M., Kleber, M. & Jahn, R. Carbon storage in loess derived surface soils from Central Germany: Influence of mineral phase variables. *J. Plant Nutr. Soil Sci.* **165**, 141–149 (2002).

95. Kahle, M., Kleber, M., Torn, M. S. & Jahn, R. Carbon storage in coarse and fine clay fractions of illitic soils. *Soil Sci. Soc. Am. J.* **67**, 1732–1739 (2003).

96. Kölbl, A., von Lützow, M. & Kögel-Knabner, I. Decomposition and distribution of 15N labelled mustard litter (Sinapis alba) in physical soil fractions of a cropland with high- and low-yield field areas. *Soil Biol. Biochem.* **38**, 3292–3302 (2006).

97. Larré-Larrouy, M. C., Blanchart, E., Albrecht, A. & Feller, C. Carbon and monosaccharides of a tropical Vertisol under pasture and market-gardening: Distribution in secondary organomineral separates. *Geoderma* **117**, 63–79 (2003).

98. Leinweber, P. & Reuter, G. The influence of different fertilization practices on concentrations of organic carbon and total nitrogen in particle-size fractions during 34 years of a soil formation experiment in loamy marl. *Biol. Fertil. Soils* **13**, 119–124 (1992).

99. Li, X. G. *et al.* Changes in soil organic carbon, nutrients and aggregation after conversion of native desert soil into irrigated arable land. *Soil Tillage Res.* **104**, 263–269 (2009).

100. Liang, A. *et al.* Soil organic carbon changes in particle-size fractions following cultivation of Black soils in China. *Soil Tillage Res.* **105**, 21–26 (2009).

101. Matus, F., Christopher, C. & Maire, C. Effects of soil texture, carbon input rates, and litter quality on free organic matter and nitrogen mineralization in Chilean rain forest and agricultural soils. *Commun. Soil Sci. Plant Anal.* **39**, 187–201 (2008).

102. McKeague, J. A. Organic matter in particle-size and specific gravity fractions of some Ah horizons. *Can. J. Soil Sci.* 499–505 (1971).

103. Nacro, H. B., Benest, D. & Abbadie, L. Distribution of microbial activities and organic matter according to particle size in a humid savanna soil (Lamto, Cote d'Ivoire). *Soil Biol. Biochem.* **28**, 1687–1697 (1996).

104. Pellegrini, A. F. A. *et al.* Low-intensity frequent fires in coniferous forests transform soil organic matter in ways that may offset ecosystem carbon losses. *Glob. Chang. Biol.* 1–14 (2021). doi:10.1111/gcb.15648

105. Piñeiro, G., Paruelo, J. M., Jobbágy, E. G., Jackson, R. B. & Oesterheld, M. Grazing effects on belowground C and N stocks along a network of cattle exclosures in temperate and subtropical grasslands of South America. *Global Biogeochem. Cycles* **23**, (2009).

106. Schmidt, M. W. I. & Kögel-Knabner, I. Organic matter in particle-size fractions from A and B horizons of a Haplic Alisol. *Eur. J. Soil Sci.* **53**, 383–391 (2002).

107. Schöning, I., Morgenroth, G. & Kögel-Knabner, I. O/N-alkyl and alkyl C are stabilised in fine particle size fractions of forest soils. *Biogeochemistry* **73**, 475–497 (2005).

108. Schöning, I., Knicker, H. & Kögel-Knabner, I. Intimate association between O/N-alkyl carbon and iron oxides in clay fractions of forest soils. *Org. Geochem.* **36**, 1378–1390 (2005).

109. Schöning, I. & Kögel-Knabner, I. Chemical composition of young and old carbon pools throughout Cambisol and Luvisol profiles under forests. *Soil Biol. Biochem.* **38**, 2411–2424 (2006).

110. Schulten, H. R., Leinweber, P. & Sorge, C. Composition of organic matter in particle-size fractions of an agricultural soil. *J. Soil Sci.* **44**, 677–691 (1993).

111. Shang, C. & Tiessen, H. Organic matter lability in a tropical oxisol: Evidence from shifting cultivation, chemical oxidation, particle size, density, and magnetic fractionations. *Soil Sci.* **162**, 795–807 (1997).

112. Shrestha, B. M., Singh, B. R., Sitaula, B. K., Lal, R. & Bajracharya, R. M. Soil Aggregate- and Particle-Associated Organic Carbon under Different Land Uses in Nepal. *Soil Sci. Soc. Am. J.* **71**, 1194–1203 (2007).

113. Sleutel, S., De Neve, S., Singier, B. & Hofman, G. Organic C levels in intensively managed arable soils – long-term regional trends and characterization of fractions. *Soil Use Manag.* **22**, 188–196 (2006).

114. Sleutel, S., De Neve, S., Németh, T., Tóth, T. & Hofman, G. Effect of manure and fertilizer application on the distribution of organic carbon in different soil fractions in long-term field experiments. *Eur. J. Agron.* **25**, 280–288 (2006).

115. Solomon, D., Lehmann, J. & Zech, W. Land use effects on soil organic matter properties of chromic luvisols in semi-arid northern Tanzania: carbon, nitrogen, lignin and carbohydrates. *Agric. Ecosyst. Environ.* **78**, 203–213 (2000).

116. Soong, J. L. *et al.* Soil properties explain tree growth and mortality, but not biomass, across phosphorus-depleted tropical forests. *Sci. Rep.* **10**, 1–13 (2020).

117. Steffens, M., Kölbl, A. & Kögel-Knabner, I. Alteration of soil organic matter pools and aggregation in semi-arid steppe topsoils as driven by organic matter input. *Eur. J. Soil Sci.* **60**, 198–212 (2009).

118. Stemmer, M., Von Lützow, M., Kandeler, E., Pichlmayer, F. & Gerzabek, M. H. The effect of maize straw placement on mineralization of C and N in soil particle size fractions. *Eur. J. Soil Sci.* **50**, 73–85 (1999).

119. Thuriès, L., Larré-Larrouy, M. C. & Feller, C. Influences of organic fertilization and solarization in a greenhouse on particle-size fractions of a Mediterranean sandy soil. *Biol. Fertil. Soils* **32**, 449–457 (2000).

120. Tiessen, H. & Stewart, J. W. B. Particle-size Fractions and their Use in Studies of Soil Organic Matter: II. Cultivation Effects on Organic Matter Composition in Size Fractions. *Soil Sci. Soc. Am. J.* **47**, 509–514 (1983).

121. Turchenek, L. W. & Oades, J. M. Fractionation of organo-mineral complexes by sedimentation and density techniques. *Geoderma* **21**, 311–343 (1979).

122. Young, J. L. & Spycher, G. Water-Dispersible Soil Organic-Mineral Particles: I. Carbon and Nitrogen Distribution. *Soil Sci. Soc. Am. J.* **43**, 324–328 (1979).

123. Zhang, H., Thompson, M. L. & Sandor, J. A. Compositional Differences in Organic Matter among Cultivated and Uncultivated Argiudolls and Hapludalfs Derived from Loess. *Soil Sci. Soc. Am. J.* **52**, 216–222 (1988).

124. Zhao, L., Sun, Y., Zhang, X., Yang, X. & Drury, C. F. Soil organic carbon in clay and silt sized particles in Chinese mollisols: Relationship to the predicted capacity. *Geoderma* **132**, 315–323 (2006).

125. Zinn, Y. L., Lal, R., Bigham, J. M. & Resck, D. V. S. Edaphic Controls on Soil Organic Carbon Retention in the Brazilian Cerrado: Texture and Mineralogy. *Soil Sci. Soc. Am. J.* **71**, 1204–1214 (2007).

126. Akala, V. A. & Lal, R. Soil organic carbon pools and sequestration rates in reclaimed minesoils in Ohio. *J. Environ. Qual.* **30**, 2098–2104 (2001).

127. Alcántara, V., Don, A., Well, R. & Nieder, R. Deep ploughing increases agricultural soil organic matter stocks. *Glob. Chang. Biol.* **22**, 2939–2956 (2016).

128. Babujia, L. C., Hungria, M., Franchini, J. C. & Brookes, P. C. Microbial biomass and activity at various soil depths in a Brazilian oxisol after two decades of no-tillage and conventional tillage. *Soil Biol. Biochem.* **42**, 2174–2181 (2010).

129. Black, K. *et al.* Carbon stock and stock changes across a Sitka spruce chronosequence on surface-water gley soils. *Forestry* **82**, 255–272 (2009).

130. Boddey, R. M. *et al.* Carbon accumulation at depth in Ferralsols under zero-till subtropical agriculture. *Glob. Chang. Biol.* **16**, 784–795 (2010).

131. Bowden, R. D. *et al.* Litter Input Controls on Soil Carbon in a Temperate Deciduous Forest. *North Am. For. Soils* S66–S75 (2014). doi:10.2136/sssaj2013.09.0413nafsc

132. Brown, S. & Lugo, A. E. Effects of forest clearing and succession on the carbon and nitrogen content of soils in Puerto Rico and US Virgin Islands. *Plant Soil* **124**, 53–64 (1990).

133. Carvalho, J. L. N. *et al.* Impact of pasture, agriculture and crop-livestock systems on soil C stocks in Brazil. *Soil Tillage Res.* **110**, 175–186 (2010).

134. Chatterjee, A., Lal, R., Shrestha, R. K. & Ussiri, D. A. N. Soil carbon pools of reclaimed minesoils under grass and forest landuses. *L. Degrad. Dev.* **20**, 300–307 (2009).

135. Dick, W. A. *et al.* Impacts of agricultural management practices on C sequestration in forest-derived soils of the eastern Corn Belt. *Soil Tillage Res.* **47**, 235–244 (1998).

136. Wieder, W. R. *et al.* SoDaH: the SOils DAta Harmonization database, an open-source synthesis of soil data from research networks, version 1.0. *Earth Syst. Sci. Data Discuss.* 1–19 (2020). doi:10.5194/essd-2020-195

137. Freixo, A. A., Machado, P. L. O. D. A., Dos Santos, H. P., Silva, C. A. & Fadigas, F. D. S. Soil organic carbon and fractions of a Rhodic Ferralsol under the influence of tillage and crop rotation systems in southern Brazil. *Soil Tillage Res.* **64**, 221–230 (2002).

138. Ganjegunte, G. K., Wick, A. F., Stahl, P. D. & Vance, G. F. Accumulation and composition of total organic carbon in reclaimed coal mine lands. *L. Degrad. Dev.* **20**, 156–175 (2009).

139. Doetterl, S. *et al.* Links among warming, carbon and microbial dynamics mediated by soil mineral weathering. *Nat. Geosci.* **11**, 589–593 (2018).

140. Jantalia, C. P. *et al.* Tillage effect on C stocks of a clayey Oxisol under a soybean-based crop rotation in the Brazilian Cerrado region. *Soil Tillage Res.* **95**, 97–109 (2007).

141. Jenkinson, D. S. The Accumulation of Organic Matter in Soil Left Uncultivated. in *Rothamsted Experimental Station Report for 1970 Part 2* 113–137 (1971).

142. Keskin, T. & Makineci, E. Some soil properties on coal mine spoils reclaimed with black locust (Robinia pceudoacacia L.) and umbrella pine (Pinus pinea L.) in Agacli-Istanbul. *Environ. Monit. Assess.* **159**, 407–414 (2009).

143. Lajtha, K., Bowden, R. D. & Nadelhoffer, K. Litter and Root Manipulations Provide Insights into Soil Organic Matter Dynamics and Stability. *Soil Sci. Soc. Am. J.* **78**, S261–S269 (2014).

144. Lilienfein, J., Qualls, R. G., Uselman, S. M. & Bridgham, S. D. Soil formation and organic matter accretion in a young andesitic chronosequence at Mt. Shasta, California. *Geoderma* **116**, 249–264 (2003).

145. Sollins, P., Spycher, G. & Topik, C. Processes of soil organic-matter accretion at a mudflow chronosequence, Mt. Shasta, California. *Ecology* **64**, 1273–1282 (1983).

146. Lorenz, K. & Lal, R. Stabilization of organic carbon in chemically separated pools in reclaimed coal mine soils in Ohio. *Geoderma* **141**, 294–301 (2007).

147. Marchão, R. L. *et al.* Carbon and nitrogen stocks in a Brazilian clayey Oxisol: 13-year effects of integrated crop-livestock management systems. *Soil Tillage Res.* **103**, 442–450 (2009).

148. Mazzoncini, M., Sapkota, T. B., Bàrberi, P., Antichi, D. & Risaliti, R. Long-term effect of tillage, nitrogen fertilization and cover crops on soil organic carbon and total nitrogen content. *Soil Tillage Res.* **114**, 165–174 (2011).

149. Nii-Annang, S., Grunewald, H., Freese, D., Huttl, R. F. & Dilly, O. Microbial activity, organic C accumulation and C-13 abundance in soils under alley cropping systems after 9 years of recultivation of quaternary deposits. *Biol. Fertil. Soils* **45**, 531–538 (2009).

150. Pierson, D. *et al.* Mineral stabilization of soil carbon is suppressed by live roots, outweighing influences from litter quality or quantity. *Biogeochemistry* **9**, (2021).

151. Quinkenstein, A., Böhm, C., Matos, E. da S., Freese, D. & Hüttl, R. F. Assessing the Carbon Sequestration in Short Rotation Coppices of Robinia pseudoacacia L. on Marginal Sites in Northeast Germany. in *Carbon Sequestration Potential in Agroforestry Systems* (ed. Kuman, B. M.) 201–216 (Springer, 2011). doi:10.1007/978-94-007-1630-8_11

152. Raiesi, F. Soil properties and C dynamics in abandoned and cultivated farmlands in a semi-arid ecosystem. *Plant Soil* **351**, 161–175 (2012).

153. Reintam, L., Kaar, E. & Rooma, I. Development of soil organic matter under pine on quarry detritus of open-cast oil-shale mining. *For. Ecol. Manage.* **171**, 191–198 (2002).

154. Roscoe, R. & Buurman, P. Tillage effects on soil organic matter in density fractions of a Cerrado Oxisol. *Soil Tillage Res.* **70**, 107–119 (2003).

155. Schipper, L. A. & Sparling, G. P. Accumulation of soil organic C and change in C:N ratio after establishment of pastures on reverted scrubland in New Zealand. *Biogeochemistry* **104**, 49–58 (2011).

156. Sever, H. & Makineci, E. Soil organic carbon and nitrogen accumulation on coal mine spoils reclaimed with maritime pine (Pinus pinaster Aiton) in Agacli-Istanbul. *Environ. Monit. Assess.* **155**, 273–280 (2009).

157. Shrestha, R. K., Lal, R. & Jacinthe, P. A. Enhancing carbon and nitrogen sequestration in reclaimed soils through organic amendments and chiseling. *Soil Sci. Soc. Am. J.* **73**, 1004–1011 (2009).

158. Williams-Linera, G. Biomass and Nutrient Content in Two Successional Stages of Tropical Wet Forest in Uxpanapa, Mexico. *Biotropica* **15**, 275 (1983).
